# Emergent reactance induced by the deformation of a current-driven skyrmion lattice

**DOI:** 10.1038/s41467-026-69698-1

**Published:** 2026-02-19

**Authors:** Matthew T. Littlehales, Max T. Birch, Akiko Kikkawa, Yasujiro Taguchi, Diego Alba Venero, Peter D. Hatton, Naoto Nagaosa, Yoshinori Tokura, Tomoyuki Yokouchi

**Affiliations:** 1 Durham University, Department of Physics, Durham, UK; 2 ISIS Neutron and Muon Source, Rutherford Appleton Laboratory, Didcot, UK; 3 RIKEN Center for Emergent Matter Science (CEMS), Wako, Japan; 4 Fundamental Quantum Science Program (FQSP), TRIP Headquarters, RIKEN, Wako, Japan; 5 Department of Applied Physics, University of Tokyo, Tokyo, Japan; 6 Tokyo College, University of Tokyo, Tokyo, Japan; 7 Present Address: Physik-Department, Technische Universität München (TUM), Garching, Germany

**Keywords:** Topological defects, Spintronics, Magnetic properties and materials, Electronic properties and materials

## Abstract

Classical electromagnetism forms the foundation of modern technology. In condensed matter systems, the Berry phase acquired by conduction electrons acts as an emergent electromagnetic field, facilitating phenomena analogous to classical electromagnetism, such as the Lorentz force and electromagnetic induction, and paving the way for next-generation spintronics. Magnetic skyrmions, spin vortices with non-trivial topology, serve as a key platform for such devices. For example, non-trivial transport responses are recognised as being induced by the emergent Lorentz force and the emergent electromagnetic induction. Despite remarkable progress in skyrmion physics, emergent reactance, in which the phase of an applied AC current is modified by emergent electromagnetism, has not been thoroughly investigated. Here, we report emergent reactance in a micro-fabricated device of the prototypical skyrmion-hosting material, MnSi. Our findings reveal that the internal deformation degrees of freedom in skyrmions are an important factor for efficient generation of the emergent reactance.

## Abstract (web summary)

From lock-in transport measurements of a skyrmion lattice in MnSi, the authors find a delay in the voltage response of the Hall effect and resistivity, arguing that its deformation leads to inertial skyrmion lattice dynamics and emergent reactance.

## Introduction

As an electron traverses a non-collinear spin texture with its spin direction adiabatically aligned with the underlying spins, the wavefunction of the electron acquires an additional quantum mechanical phase, termed Berry’s phase^1^. The Berry phase acts as an effective electromagnetic field, also known as the emergent electromagnetic field, and is responsible for a wide variety of quantum electronic phenomena^2–5^.

A pivotal playground for emergent electromagnetic fields is offered by topological spin structures, known as skyrmions. A skyrmion is a particle-like spin texture characterised by a topological winding number *N*_Sk_ = − 1 and can form a hexagonal lattice (SkL) as an equilibrium state in non-centrosymmetric and frustrated helimagnets^6–8^. Because of their topologically non-trivial and non-coplanar magnetic structure, skyrmions generate an emergent magnetic field **b**_em_, which deflects electron trajectories similarly to the normal Hall effect, resulting in an additional Hall conductivity known as the topological Hall effect (THE)^9^.

**Fig. 1:**
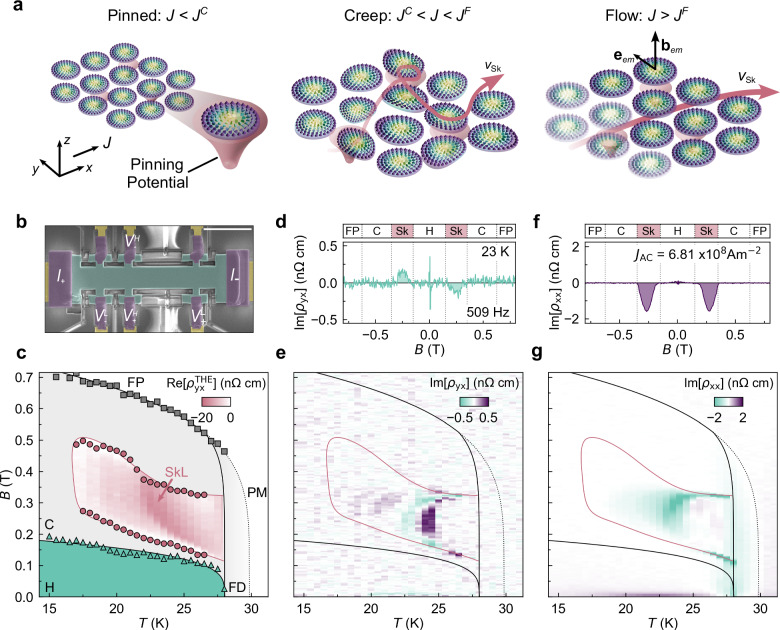
Emergent reactance in the SkL phase of MnSi. **a** Schematic of the dynamical phases of SkL with increasing current density. **b** Scanning electron micrograph of our micro-fabricated MnSi device (Scale bar = 10 *μ*m), labelled with current *I*_±_, longitudinal voltage ${V}_{\pm }^{{\rm{L}}}$, and Hall voltage ${V}_{\pm }^{{\rm{H}}}$ electrodes. **c** Magnetic phase diagram of MnSi device with helical (H), conical (C), skyrmion lattice (SkL), field polarised (FP), fluctuation disordered (FD), and paramagnetic (PM) phases determined from the Hall resistivity *ρ*_*yx*_. The SkL phase is overlaid with a colour map of ${\rho }_{yx}^{{\rm{THE}}}$. A detailed procedure for determining the phase boundaries and topological Hall resistivity is provided in Methods and Supplementary Note 2. **d**, **e** Magnetic field dependence at 23 K (**d**) and colour map on the magnetic field-temperature plane (**e**) of the transverse reactance Im[*ρ*_*yx*_]. **f**, **g** Magnetic field dependence at 23 K (**f**) and colour map on the magnetic field-temperature plane (**g**) of the longitudinal reactance Im[*ρ*_*xx*_]. The transverse and longitudinal reactance were measured with *J*_AC_ = 6.81 × 10^8^ A m^−2^ and frequency of *f* = 509 Hz. The feature at *B* = 0 T in (**d**) is a result of the extrinsic reactance from the measurement setup (see Supplementary Note 1).

An emergent electric field is associated with the dynamics of the SkL. When an electric current is applied to the SkL, spin-transfer torque induces its motion, leading to dynamic transitions or crossovers at finite temperature^10–12^. At low current densities, the SkL is trapped by pinning potentials. Once the current density *J* exceeds the pinning threshold *J*^C^, the SkL undergoes creep motion, where skyrmions hop between adjacent pinning sites accompanied by their internal deformation. Notably, this deformation manifests as an effective skyrmion mass, leading to inertial motion^12,13^. Finally, when *J* surpasses the flow threshold *J*^F^, the SkL flows freely through pinning centres, reducing its deformation and dynamically reordering due to the relative weakening of the pinning force^14,15^. Schematically, this is demonstrated in Fig. 1a. An emergent electric field can be generally described by

1 $${e}_{{\rm{em}},i}=\frac{h}{2\pi e}{\bf{n}}\cdot ({\partial }_{i}{\bf{n}}\times {\partial }_{t}{\bf{n}}),$$

where **n** is a unit vector parallel to the direction of spins^16–18^. Since Eq. (1) involves the time derivative of the spin direction, the current-driven dynamics of skyrmions can induce the emergent electric field. For instance, for the translational motion of the SkL with velocity **v**_Sk_, Eq. (1) reduces to **e**_em_ = − **v**_Sk_ × **b**_em_. This emergent electric field opposes the THE, which is experimentally observed as a reduction in the topological Hall resistivity^12,16^. In particular, in the clean limit of the flow regime, the SkL may catch up to the conduction electron velocity, leading to a complete cancellation of the topological Hall resistivity by the emergent electric field due to emergent Galilean relativity^12,16,19^.

Another recently discovered platform for exploring emergent electric fields is the current-induced deformation of non-collinear spin textures such as domain walls and helices. A non-collinear spin texture driven by an electric current undergoes spatial and temporal deformation. This deformation can be described by spin-tilting and phason modes, whose excitation notably induces an out-of-phase emergent electric field relative to the input AC current, equivalent to a reactance^20,21^. Hereafter, we refer to the out-of-phase response arising from the emergent electric field as “emergent reactance”. This emergent reactance contrasts with the previously reported emergent electric field induced by the translational motion of the SkL, since the latter induces an in-phase Hall emergent electric field. In particular, the modulation of the input current phase plays a crucial role in modern AC circuits, which is traditionally governed by capacitors and inductors operating based on classical electromagnetism. Importantly, the magnitude of the emergent reactance is inversely proportional to the cross-sectional area of the device. Thus, the emergent reactance provides a novel working principle based on emergent electromagnetism for nanoscale electric devices, expanding the potential for applications^20^. So far, emergent reactance has been demonstrated in a limited number of helimagnets and domain wall systems^22–24^, while it has been intensively investigated theoretically^25–30^. However, no strategy has been established to obtain a sufficiently large emergent reactance for practical applications.

In this work, we report the observation of emergent reactance generated by the deformation of the SkL under creep motion. We observe longitudinal and transverse reactance signals as the skyrmion lattice undergoes creep motion, in which the skyrmions deform while moving. The transverse reactance is attributed to the emergent electric field associated with the inertial translational motion arising from the skyrmion's effective mass. In contrast, the longitudinal reactance results from the emergent electric fields generated by the phason and spin-tilting modes excited by their deformation. Notably, since the threshold current density of the SkL is lower than that of helices and ferromagnetic domain walls^31,32^, our findings offer an advantage for potential applications.

## Results

For this study, we chose the prototypical skyrmion host MnSi^6,9,16,33^. We fabricated a microscale thin-plate of MnSi by focused-ion beam (FIB), and mounted it onto a CaF_2_ substrate (Fig. 1b), to apply high current densities and enhance the signal-to-noise ratio. The resistance and reactance are measured using standard lock-in techniques (see ‘Methods’ for details). In Fig. 1c, we show the magnetic phase diagram of our device together with a colour map of the topological Hall resistivity in the SkL phase. In addition to the enhancement of two-dimensionality, the compressive strain induced by the differential thermal expansion between the CaF_2_ substrate and the MnSi thin plate stabilises the skyrmion phase over an extended temperature region compared with bulk samples^34^. First, in Fig. 1d, f, we present the magnetic field dependence of the transverse and longitudinal reactances (Im[*ρ*_*yx*_] and Im[*ρ*_*xx*_]) measured at 23 K using a high AC current density *J*_AC_ = 6.81 × 10^8^ A m^−2^ at 509 Hz (see ‘Methods’ and Supplementary Note 1 for analysis of Im[*ρ*_*yx*_] and Im[*ρ*_*xx*_]). Clearly, a non-zero signal is observed only within the SkL phase. Furthermore, *B* − *T* colour maps presented in Fig. 1e and g also highlight nonzero reactance signals only within the SkL phase.

### Dynamical phases of skyrmion lattice in MnSi

**Fig. 2:**
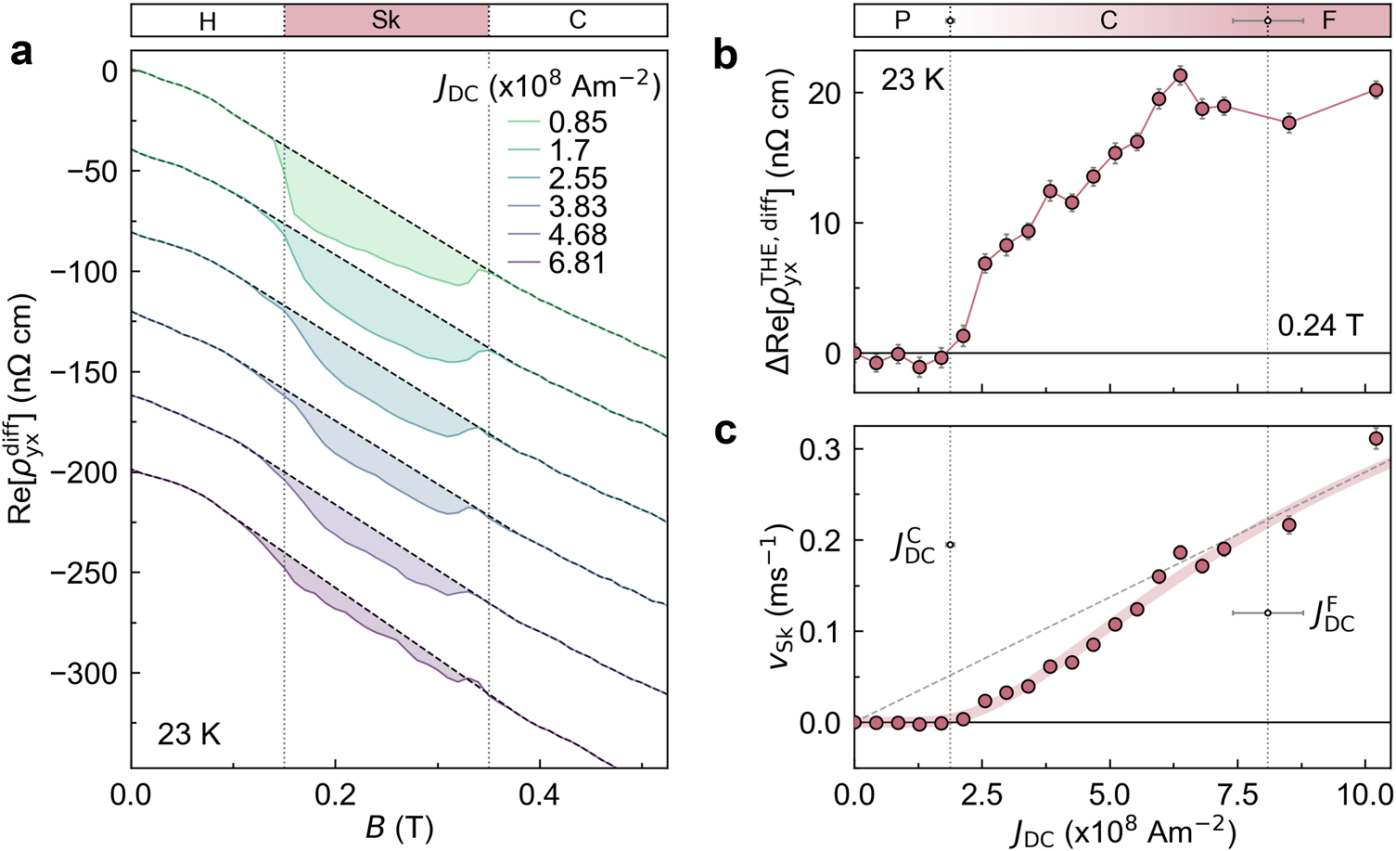
Emergent electrodynamics of the SkL motion driven by DC current. **a** Magnetic field dependence of the differential Hall resistivity ${\rm{Re}}[{\rho }_{yx}^{{\rm{diff}}}]$ for various DC current bias *J*_DC_. The dashed lines denote a spline fit to the regions outside of the SkL phase (see Supplementary Note 2). **b** Change in the topological Hall resistivity $\Delta {\rm{Re}}[{\rho }_{yx}^{{\rm{THE}},{\rm{diff}}}]={\rm{Re}}[{\rho }_{yx}^{{\rm{THE}},{\rm{diff}}}]({J}_{{\rm{DC}}})$ −  ${\rm{Re}}[{\rho }_{yx}^{{\rm{THE}},{\rm{diff}}}]({J}_{{\rm{DC}}}=0\,{{\rm{A\; m}}}^{-2})$ in the SkL phase at 0.24 T. **c**$\Delta {\rm{Re}}[{\rho }_{yx}^{{\rm{THE}},{\rm{diff}}}]$ converted into skyrmion velocity *v*_Sk_ using Eq. (2). We plot a red line as a guide to the eye. The dashed line corresponds to 0.75*v*_*e*_ (see also ‘Methods’ and Supplementary Note 3). The dotted lines in (**b**) and (**c**) indicate the crossover points of pinned-creep and creep-flow, with errors indicated in the top subplot of **b**. For the procedure of error estimation, see ‘Methods’. These measurements were performed by locking into a small oscillation of *J*_AC_ = 1.74 × 10^7^ A m^−2^ at a frequency of *f* = 509 Hz, modulated on a DC bias, *J*_DC_. Error bars in (**b**) and (**c**) correspond to the standard deviation of the signal estimated in the field polarised regime. Where not visible, error bars are smaller than the data points.

To elucidate the mechanism responsible for the longitudinal and transverse reactance signals, we first determine the dynamical phases of the SkL from the current-density dependence of the topological Hall resistivity. In Fig. 2a, we present the magnetic field dependence of the differential Hall resistivity ${\rm{Re}}[{\rho }_{yx}^{{\rm{diff}}}]$ for selected DC bias current *J*_DC_. Here, the DC bias current drives the SkL, and a superimposed small AC current is used to measure ${\rm{Re}}[{\rho }_{yx}^{{\rm{diff}}}]$ (see ‘Methods’ for details). As *J*_DC_ increases, the topological Hall resistivity (the shaded regions) decreases. This is further highlighted in Fig. 2b, where the *J*_DC_ dependence of the change in the topological Hall resistivity $\Delta {\rm{Re}}[{\rho }_{yx}^{{\rm{THE,diff}}}]$ is plotted for the SkL phase, and displays non-monotonic behaviour closely resembling that observed in bulk MnSi^16^ (see Supplementary Note 2 for estimation of $\Delta {\rm{Re}}[{\rho }_{yx}^{{\rm{THE,diff}}}]$). The emergent electric field generated by a moving SkL is proportional to its velocity, which can be expressed as:

2 $${v}_{{\rm{Sk}}}=\frac{J\Delta {\rho }_{yx}( \, J)}{P{b}_{{\rm{em}}}},$$

in which *Pb*_em_ is the effective emergent magnetic field Δ*ρ*_*yx*_(*J*)^12^. Using Eq. (2), we estimate the SkL velocity from $\Delta {\rm{Re}}[{\rho }_{yx}^{{\rm{THE}},{\rm{diff}}}]$, where *Pb*_em_ is estimated to be  − 0.79(3) T (see Supplementary Fig. 2). Figure 2c presents the DC current density dependence of the estimated SkL velocity, highlighting three distinct regions: (1) *v*_Sk_ = 0 below ${J}_{{\rm{DC}}}^{{\rm{C}}}$; (2) a nonlinear increase in *v*_Sk_ between ${J}_{{\rm{DC}}}^{{\rm{C}}}$ and ${J}_{{\rm{DC}}}^{{\rm{F}}}$; and (3) *v*_Sk_ proportional to *J*_DC_ and approaches the electron velocity *v*_*e*_ above ${J}_{{\rm{DC}}}^{{\rm{F}}}$ (see also Supplementary Note 3). Based on previous studies^10–12^, we identify the dynamical phases as follows: (1) the pinned state below${J}_{{\rm{DC}}}^{{\rm{C}}}$, (2) the creep region between ${J}_{{\rm{DC}}}^{{\rm{C}}}$ and ${J}_{{\rm{DC}}}^{{\rm{F}}}$, and (3) the flow regime above ${J}_{{\rm{DC}}}^{{\rm{F}}}$. These boundaries (${J}_{{\rm{DC}}}^{{\rm{C}}}$ and ${J}_{{\rm{DC}}}^{{\rm{F}}}$) are typically smeared by thermal fluctuations and are regarded as crossovers. We determined them using the fitting procedure described in the ‘Methods’ and Supplementary Note 8. Estimated ${J}_{{\rm{DC}}}^{{\rm{C}}}$ and ${J}_{{\rm{DC}}}^{{\rm{F}}}$ are of the same order as those of the microfabricated sample of Gd_2_PdSi_3_^12^, but 2-3 orders of magnitude larger than those in bulk single crystals of MnSi^16^. However, the *v*_sk_ − *J*_DC_ profile of our device is qualitatively similar to that in bulk single crystals of MnSi^11,16^, and scales with *J*_DC_ nearly identically in the flow region (see Supplementary Note 3). Accordingly, the higher thresholds can be attributed to enhanced collective pinning, arising from an increased number of pinning sites introduced during the FIB fabrication process and confinement effects in microfabricated samples^12,35,36^. Therefore, aside from the higher thresholds, there is no essential difference in the current-driven properties of the SkL between our device and bulk samples. We note that, above the flow region, the re-deformation of the SkL via spin-wave emission has been theoretically predicted^37^. However, the current density used in this study is four orders of magnitude smaller than that required to induce such re-deformation.

### Emergent reactance in skyrmion lattice phase

**Fig. 3:**
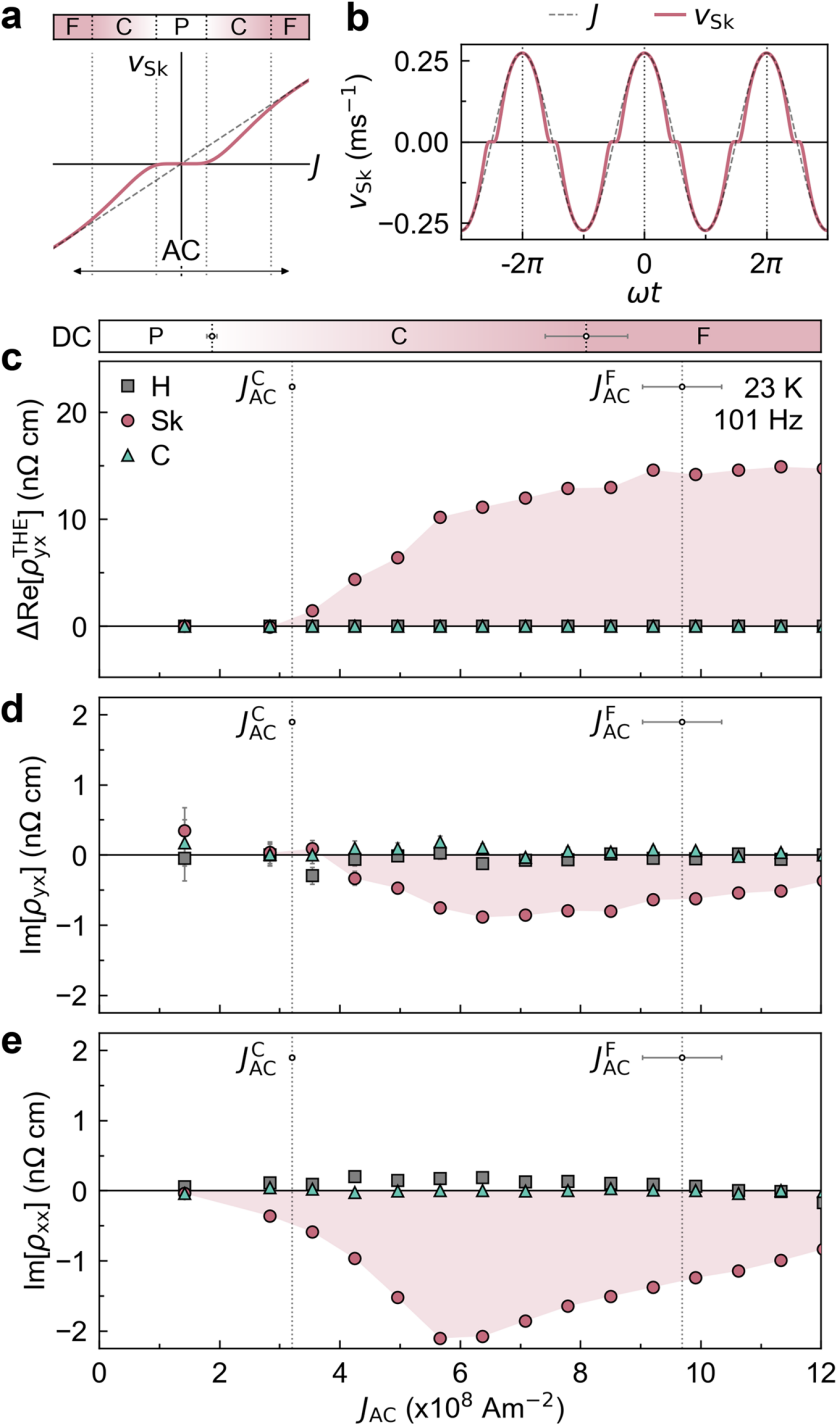
Current density dependence of emergent reactance in MnSi. **a**,** b** Schematic representation of the skyrmion velocity curve for a cycle of AC. The voltage response follows the SkL velocity, but the time-averaged response is primarily governed by the dynamical phase where the current reaches its peak. **c**–**e** AC amplitude, *J*_AC_, dependence of $\Delta {\rm{Re}}[{\rho }_{yx}^{{\rm{THE}}}]$ (**c**), Im[*ρ*_*yx*_] (**d**), and Im[*ρ*_*xx*_] (**e**) for helical (H, grey squares), skyrmion lattice (SkL, red circles), and conical phases (C, teal triangles), measured at 23 K and 101 Hz. The threshold current densities for the creep, ${J}_{{\rm{AC}}}^{{\rm{C}}}$, and flow, ${J}_{{\rm{AC}}}^{{\rm{F}}}$, determined from $\Delta {\rm{Re}}[{\rho }_{yx}^{{\rm{THE}}}]$ are indicated by the dotted vertical lines, with errors on threshold values highlighted with horizontal error bars. For the procedure of error estimation, see ‘Methods’. The threshold current densities in the DC measurement are shown at the top of **c**. Error bars in **c**–**e** correspond to the standard deviation of the signal estimated in the field polarised regime. Where not visible, error bars are smaller than the data points.

Armed with the knowledge of the dynamical phases of the SkL in our device, we measured the AC current density dependence of the reactance signals to uncover their origins. For AC current measurements without *J*_DC_, the current passes through each sequential dynamical phase (Fig. 3a, b). Consequently, the current density dependencies measured with DC and AC currents are not identical (see Supplementary Note 5). However, since the voltage response is considerably influenced by the dynamical phase at the peak of the AC current, the dependence on the AC current amplitude provides valuable insights.

In Fig. 3c, we present the change in the topological Hall resistivity $\Delta {\rm{Re}}[{\rho }_{yx}^{{\rm{THE}}}]$ measured at a frequency of *f* = 101 Hz as a function of the amplitude of the AC current *J*_AC_, demonstrating a similar profile to that of the DC bias current dependence (Fig. 2b). From $\Delta {\rm{Re}}[{\rho }_{yx}^{{\rm{THE}}}]$ in the SkL phase, we determine the creep (${J}_{{\rm{AC}}}^{{\rm{C}}}$) and flow (${J}_{{\rm{AC}}}^{{\rm{F}}}$) thresholds for AC current by using the same fitting procedure to that used to determine ${J}_{{\rm{DC}}}^{{\rm{C}}}$ and ${J}_{{\rm{DC}}}^{{\rm{F}}}$ (see ‘Methods’). At this low frequency, these values are close to ${J}_{{\rm{DC}}}^{{\rm{C}}}$ and ${J}_{{\rm{DC}}}^{{\rm{F}}}$. We note that a small difference between the threshold values for the AC and DC threshold values mainly arises from their frequency dependence (see the discussion below). In Fig. 3d, e, we show the transverse and longitudinal reactance (Im[*ρ*_*yx*_] and Im[*ρ*_*xx*_]). While no reactance is observed for the helical and conical phases, nonzero reactance appears in the SkL phase. Notably, in the SkL phase, both the transverse and longitudinal reactance remain zero in the pinned region, reach their maximum values in the creep region, and then decrease in the flow region. In addition, in the measurement using DC bias current, prominent reactance signals are also observed mainly in the creep region (see Supplementary Note 4). Therefore, these results suggest that the observed reactance is associated with the creep motion.

### Frequency and current density dependence

**Fig. 4:**
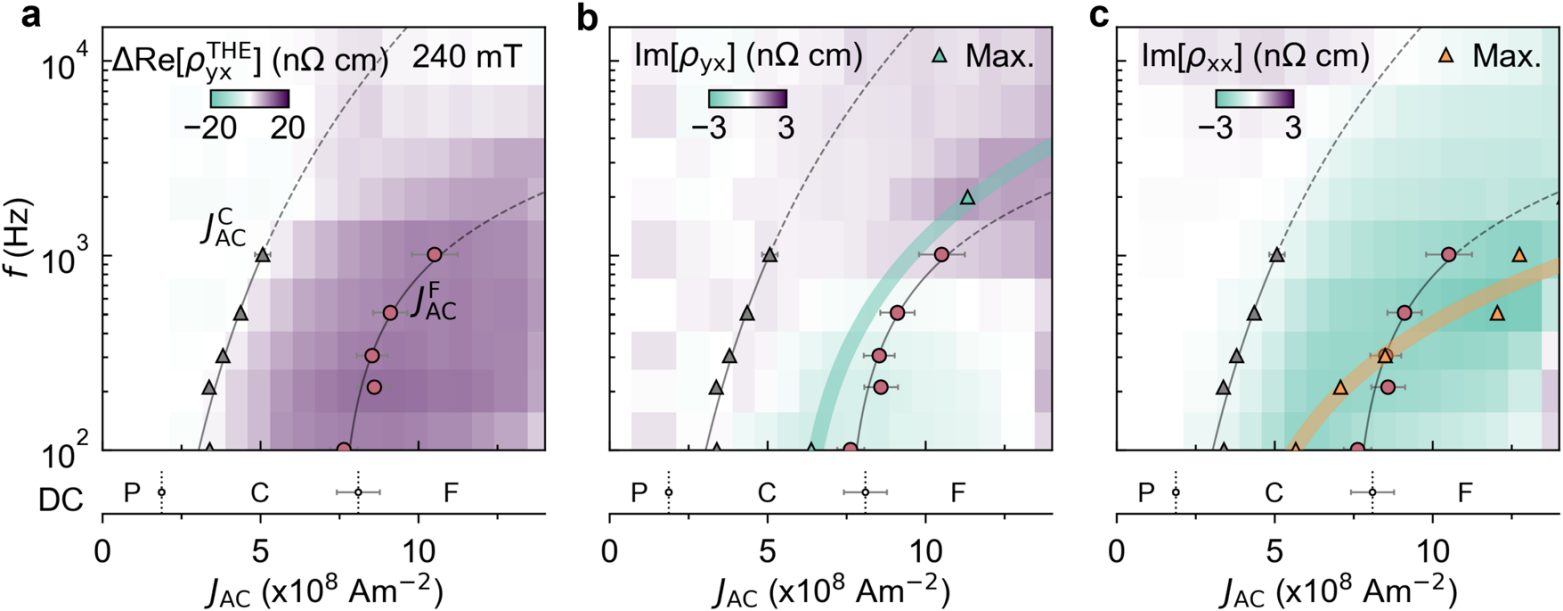
Frequency and current dependence of emergent reactance. **a**-**c** Colour maps of $\Delta {\rm{Re}}[{\rho }_{yx}^{{\rm{THE}}}]$ (**a**), Im[*ρ*_*yx*_] (**b**), and Im[*ρ*_*xx*_] (**c**) on the *J*_AC_- *f* plane, measured at 23 K for *B* = 0.24 T. The creep and flow thresholds with error bars, as characterised from $\Delta {\rm{Re}}[{\rho }_{yx}^{{\rm{THE}}}]$, are overlaid in each plot by the grey triangles and red circles, respectively. The creep and flow thresholds with error bars in the DC case are indicated at the bottom of each panels. In (**b**) and (**c**), triangular markers denote the maximum and minimum of Im[*ρ*_*yx*_] and Im[*ρ*_*xx*_] in the *J*_AC_-*f* plane. The teal and orange lines in (**b**) and (**c**), respectively, show a guide of the maximum/minimum values for increasing frequency.

To provide further insight into the origin of these signals, we repeated such AC current-dependent measurements at a range of frequencies and plotted colour maps of $\Delta {\rm{Re}}[{\rho }_{yx}^{{\rm{THE}}}]$, Im[*ρ*_*yx*_] and Im[*ρ*_*xx*_] in the frequency-current density plane in Fig. 4a–c. The creep and flow thresholds are indicated by solid lines. Extrapolating ${J}_{{\rm{AC}}}^{{\rm{C}}}$ and ${J}_{{\rm{AC}}}^{{\rm{F}}}$ to the low-frequency limit closely matches ${J}_{{\rm{DC}}}^{{\rm{C}}}$ and ${J}_{{\rm{DC}}}^{{\rm{F}}}$. In addition, with increasing frequency, ${J}_{{\rm{AC}}}^{{\rm{C}}}$ and ${J}_{{\rm{AC}}}^{{\rm{F}}}$ increase monotonically. This is because the inertia of the skyrmion causes a retardation of its motion, which particularly impedes rapid skyrmion motion at higher frequencies and raises the threshold current densities. Both Im[*ρ*_*yx*_] and Im[*ρ*_*xx*_] (Fig. 4b, c) appear above ${J}_{{\rm{AC}}}^{{\rm{C}}}$, and reach their maximum mostly within the creep region (the triangular points in Fig. 4b, c), again implying that skyrmion dynamics in the creep region play an important role in both Im[*ρ*_*yx*_] and Im[*ρ*_*xx*_]. In addition, with increasing frequency, Im[*ρ*_*yx*_] changes sign from negative to positive around *f* ≈ 500 Hz, in stark contrast to Im[*ρ*_*xx*_], which maintains its sign. This behaviour strongly suggests that, although they are both related to the SkL dynamics in the creep region, they may have fundamentally different origins. We note that the peak value of Im[*ρ*_*xx*_] shifts into the flow region at high frequencies. This originates from the AC current traversing the creep region before entering the flow region (see Supplementary Note 5 for details).

### Origin of emergent reactance

Since Im[*ρ*_*yx*_] and Im[*ρ*_*xx*_] are observed in the moving SkL phase, it is natural to consider their origin in terms of emergent electric fields. The transverse reactance Im[*ρ*_*yx*_] can be explained by the inertial translational motion of the SkL. The velocity of the translational motion of the skyrmion **v**_sk_ obeys the Thiele equation: ${m}_{{\rm{sk}}}{\dot{\mathbf{v}}_{{\rm{sk}}}}+{\mathcal{G}}\times ({{\bf{v}}}_{{\rm{e}}}-{{\bf{v}}}_{{\rm{sk}}})+{\mathcal{D}}({\beta {\bf{v}}}_{{\rm{e}}}-\alpha {{\bf{v}}}_{{\rm{sk}}})+{\boldsymbol{\nabla }}{V}_{{\rm{pin}}}=0$, where ${\mathcal{G}}$, ${\mathcal{D}}$, *β*, *α*, *V*_pin_, and *m*_sk_ are the gyro-coupling vector, dissipative force tensor, dimensionless constant characterizing the nonadiabatic electron spin dynamics, Gilbert damping constant, pinning potential, and the skyrmion mass, respectively. Here, the skyrmion mass arises because the deformation of skyrmions allows energy to be stored, which is renormalized into a mass-like term in the Thiele equation^38,39^. Experimentally, inertial dynamics arising from this mass-like term have been reported in isolated skyrmion systems^13,40^ and in SkL^12^. In the present case, since the skyrmion deformation occurs in the creep region, *m*_sk_ is nonzero in this region. Consequently, in the creep region, the time derivative of **v**_Sk_ and the nonlinear term **∇***V*_pin_ in the Thiele equation cause the phase of **v**_Sk_ to shift relative to that of the input AC current. As a result, the phase of the emergent electric field induced by the translational motion of the SkL **e**_em_ = − **v**_Sk_ × **b**_em_ also shifts with respect to the input AC current, generating the transverse reactance component given by ${\rm{Im}}[{\rho }_{yx}]=P{b}_{{\rm{em}}}{v}_{{\rm{Sk}}}^{{\prime\prime} }/{j}_{{\rm{AC}}}$, where ${v}_{{\rm{sk}}}^{{\prime\prime} }$ is the phase-shifted skyrmion velocity (see Supplementary Note 6). We note that ${v}_{{\rm{Sk}}}^{{\prime\prime} }$ can be positive or negative, depending on the damping parameters, the shape of the potential, and the frequency, which is responsible for the sign change of Im[*ρ*_*yx*_] observed in the *B*-*T* plane (Fig. 1e) and the frequency-current plane (Fig. 4b) (see Supplementary Note 6 for details). In contrast, in the flow region, since *m*_Sk_ = 0 and **∇***V*_pin_ ≈ 0 due to the absence of internal deformation and a relative reduction of the pinning force, the skyrmion velocity equals the electron velocity. Therefore, **e**_em_ = − **v**_Sk_ × **b**_em_ does not exhibit a phase shift, and thus the transverse reactance component vanishes.

In contrast, the longitudinal reactance cannot be explained by the inertial translational motion of the SkL. If the skyrmion velocity had a transverse component due to the skyrmion Hall effect, inertial translational SkL motion would generate a longitudinal reactance due to the projection of the emergent electric field along the longitudinal direction. This mechanism is proposed to explain the observed longitudinal reactance in ref. ^41^. In this case, however, Im[*ρ*_*xx*_] and Im[*ρ*_*yx*_] should have the same sign because the sign of the skyrmion Hall angle is determined by the topological charge and remains constant. Thus, this mechanism cannot explain why only Im[*ρ*_*yx*_] changes its sign in the *B*-*T* plane (Fig. 1e, g) and the frequency-current plane (Fig. 4b, c). However, since the longitudinal reactance is also observed in the creep region, where the SkL moves while deforming, the dynamics and deformation of the SkL must play an important role. One plausible explanation is that the longitudinal reactance Im[*ρ*_*xx*_] arises directly from the internal deformation of the skyrmions themselves. The magnetic moment of the deformed SkL can be described as a superposition of three helices, including its deformation as follows:

3 $${\bf{m}}={m}_{z}\widehat{{\bf{z}}}+\mathop{\sum }\limits_{i=a,b,c}{{\bf{m}}}_{i},$$

4 $${{\bf{m}}}_{i}={m}_{h}\left({\beta }_{i}{\widehat{{\bf{Q}}}}_{i}+\sqrt{1-{\beta }_{i}^{2}}{{\bf{l}}}_{i}\right),$$

5 $${{\bf{l}}}_{i}=\widehat{{\bf{z}}}\cos ({{\bf{Q}}}_{i}\cdot {\bf{r}}+{\varphi }_{i})+({\widehat{{\bf{Q}}}}_{i}\times \widehat{{\bf{z}}})\sin ({{\bf{Q}}}_{i}\cdot {\bf{r}}+{\varphi }_{i}),$$

where **Q**_*i*_ and ${\widehat{{\bf{Q}}}}_{i}$ are the *Q* vector for each helix and its unit vector. Here, *φ*_*i*_ and *β*_*i*_ are the phason and spin-tilting mode for each helix, respectively, which characterise the SkL deformation^42^. For a single-*Q* helix, the longitudinal emergent reactance is induced by the excitation of these modes^20,43^. Likewise, their excitation in the SkL by its deformation in the creep regime is expected to generate a longitudinal emergent reactance. For example, when the spin-tilting mode parallel to the current direction (*β*_*x*_) is excited, it can be shown that the longitudinal emergent electric field is proportional to its time derivative (*dβ*_*x*_/*dt*), while the transverse reactance becomes zero, similar to the helix (see Supplementary Note 6).

Although in principle the helical and conical states can induce an emergent reactance, it was not observed in these phases. For the conical states, **Q** aligns parallel to *B* in the present experimental setup, and explicitly perpendicular to *J*, forbidding excited phason and spin tilting modes^20^. For the helical state, **Q** prefers to align along the in-plane <111> directions^44^, therefore, it is possible to excite these modes and generate a longitudinal emergent reactance. However, since the threshold current density for the helical motion is much higher than that for the SkL^32^, we expect no emergent electric field to be generated under the current densities investigated in the present work.

## Discussion

Our scenario also clearly explains the temperature dependencies of Im[*ρ*_*yx*_] and Im[*ρ*_*xx*_] (Fig. 1e, g), both of which exhibit large signals at the centre of the SkL phase in the *B*-*T* phase diagram (22–25 K). The small emergent reactance signals above and below this temperature region can be explained as follows (see Supplementary Note 7 for a detailed discussion of the temperature dependence). First, in the high temperature region (25–28 K), Im[*ρ*_*yx*_] at *J*_AC_ = 6.81 × 10^8^ A m^−2^ falls below the noise level. This occurs because the SkL remains pinned at this current density due to the increase in the critical current density required for creep motion at higher temperatures, as reported in ref. ^16^. This is a natural extension of the Anderson-Kim theory^45^. Close to the fluctuation-disordered boundary, strong thermal fluctuations soften the magnetic moment of the SkL, thereby enhancing the pinning effects. In fact, the topological Hall resistivity at 26 K does not decrease even at *J*_AC_ = 6.81 × 10^8^ A m^−2^ (Supplementary Fig. 7e), supporting the conclusion that the SkL remains pinned. In contrast, while Im[*ρ*_*xx*_] is also substantially reduced in this temperature range, a small positive Im[*ρ*_*xx*_]  ≈ 0.25 n*Ω* cm is observed, which may result from an emergent reactance due to the current-induced deformation of the pinned SkL^41,46^. Secondly, Im[*ρ*_*yx*_] and Im[*ρ*_*xx*_] also become small at low temperatures below 22 K. In this temperature range, the topological Hall resistivity measured at low current density is smaller than that in the centre of the SkL phase, indicating that the skyrmion state is metastable and coexists with the conical phase, which leads to small Im[*ρ*_*yx*_] and Im[*ρ*_*xx*_].

In summary, we have demonstrated both transverse and longitudinal emergent reactance in the creep motion regime of the SkL. For both cases, the deformation of the SkL plays a crucial role, indirectly for transverse reactance and directly for longitudinal reactance. We attribute the transverse reactance to the phase shift of the emergent electric field resulting from inertial translational motion of the SkL, which arises from the renormalisation of its deformation into an effective skyrmion mass. In contrast, the longitudinal emergent reactance is attributed to the emergent electric field induced by the excitation of the phason and spin tilting modes during the deformation of the SkL. Our work highlights the importance of internal deformation degrees of freedom in skyrmions for the generation of an emergent electric field, an aspect that has been overlooked. Notably, the low critical current densities required for SkL creep motion may provide an advantage over helical and domain wall systems in generating emergent reactance for spintronic applications^22,24^. Furthermore, nanoscale topological spin textures other than conventional skyrmions, such as skyrmion bundles^47^, hedgehogs^48^, and hopfions^49^ can potentially generate emergent reactance through the same mechanisms.

## Methods

### Sample preparation and device fabrication

Single crystals of MnSi were grown by the Czochralski method and oriented using a Laue x-ray camera. A FEI Helios 5UX focused ion beam instrument was used to fabricate the thin-plate devices from the single crystal. First, a large slab of material was created by means of Ga ion milling. Then, the slab was lifted from the single crystal and attached to a Cu TEM grid using an EasyLift micromanipulator. Subsequently, the slab was thinned to below 1 *μ*m and shaped using Ga ion milling processes. Au electrical contacts were deposited onto a CaF_2_ substrate using maskless UV lithography and electron beam evaporation. The shaped MnSi thin plate was then mounted onto the CaF_2_ substrate using the micromanipulator and Pt deposition so that the sample itself directly touched the Au electrical contacts. Finally, the sample was shaped into the Hall bar geometry via Ga ion milling. In all measurements, the direction of the current *J* is oriented along the long edge of the device, while the externally applied magnetic field *B* is applied out-of-plane. Both *J* and *B* are oriented along mutually perpendicular <100> type crystal directions.

### Transport measurements

All transport measurements were performed within a Quantum Design Physical Properties Measurement System (PPMS). The MnSi devices were mounted on a standard PPMS puck using GE varnish. Cu wires were used to contact the substrate with a combination of solder and colloidal silver paste. Measurements of the longitudinal and Hall resistance and reactance were taken simultaneously using the lock-in technique (Stanford Instruments SR830). For the longitudinal resistivity Re[*ρ*_*xx*_] and the transverse resistivity Re[*ρ*_*yx*_], the data were symmetrized and anti-symmetrized in the conventional manner, i.e. ${\rm{Re}}[{\rho }_{xx}]=\{{\rm{Re}}[{\rho }_{xx}^{{\rm{measured}}}(+B)]+{\rm{Re}}[{\rho }_{xx}^{{\rm{measured}}}(-B)]\}/2$ and $\rm{Re}[{\rho }_{yx}]=\{{\rm{Re}}[{\rho }_{yx}^{{\rm{measured}}}(+B)]-{\rm{Re}}[{\rho }_{yx}^{{\rm{measured}}}(-B)]\}/2$. For the longitudinal reactance Im[*ρ*_*xx*_] and transverse reactance Im[*ρ*_*yx*_], the recorded signals were first corrected for extrinsic contributions (see Supplementary Note 1 for details). The corrected data were then symmetrized and anti-symmetrized with respect to the magnetic field. Since the longitudinal reactance is symmetric with respect to the magnetic field^21^, it was symmetrized with respect to the magnetic field. In contrast, the transverse reactance originates from the emergent electric field due to the translational motion of skyrmions, given by **e**_em_ = − **v**_sk_ × **b**_em_. In this case, when the magnetic field is reversed, the emergent magnetic field **b**_em_ is reversed, leading to the sign change of **e**_em_. Consequently, the transverse reactance is anti-symmetric with respect to the magnetic field, and thus it was anti-symmetrized.

For the AC+DC measurements, a small AC oscillation was superimposed on a large DC bias using a Keithley 6221 current source. The AC peak amplitude was set to 1.74 × 10^8^ A m^−2^ at a frequency of 509 Hz, and the output TTL pulse was used as the lock-in reference signal. In this case, we technically measure the gradient of the I-V curve according to $\frac{dV}{dI}$, which we convert into the differential resistivity following ${\rho }^{{\rm{diff}}}=t\frac{dV}{dI}$, where *t* denotes the sample thickness.

### Determination of magnetic phase boundaries

The phase boundaries are calculated for measurements taken with a low current density of *J*_AC_ = 4.26 × 10^8^ A m^−2^, such as to capture the equilibrium skyrmion phase, avoiding potential expansions due to current-induced skyrmion generation and Joule heating effects. The higher current density facilitates the extraction of the phase boundaries while being qualitatively similar to those observed at lower *J*_AC_ (see supplementary Note 2). Considering each isothermal magnetic field sweep individually, we first subtract the ordinary Hall effect by fitting a straight line to the resistivity where ∣*B*∣ > 0.7 T. After subtraction, each magnetic phase (helical, conical, SkL, field-polarised) displays a different gradient in ${\rm{Re}}[{\rho }_{yx}-{\rho }_{yx}^{{\rm{OHE}}}]$ versus *B*. Accordingly, to estimate the phase boundaries, we first fit a straight line to the approximate centre of each phase, determined by eye. We then define the estimated phase boundary as the intersection between adjacent extrapolated fits. The solid lines between helical to conical *B*_H_ (green triangles in Fig. 1c), and conical to field polarised *B*_c_ (grey squares in Fig. 1c) boundaries are fit according to the following phenomenological order parameter equation:

6 $${B}_{{\rm{H}},{\rm{c}}}=\left\{\begin{array}{ll}0 \hfill & T\ge {T}_{{\rm{C}}}\\ A{(1-\frac{T}{{T}_{{\rm{C}}}})}^{\beta } & T\le {T}_{{\rm{C}}}\end{array}\right.,$$

where *A*, *β* are fitting parameters, and *T*_C_ is a fitting parameter which refers to the transition temperature. For the H transition, we determine *T*_C_ = 28.01(2) K. The solid line for the conical to field polarised transition is determined by fitting the grey squares and fixing *T*_C_ = 28.01(2) K. Allowing *T*_C_ to vary during this transition leads to *T*_C_ = 29.9(5) K. The difference in these two transition temperatures accurately captures the fluctuation-disordered regime, closely replicating the phase boundaries in bulk MnSi^50^.

### Determination of creep and flow thresholds

The definitions of the pinned, creep, and flow regions are somewhat difficult to define, since their boundaries typically appear as smeared crossovers caused by thermally activated processes rather than sharp transitions. In this study, to systematically and precisely determine the creep (*J*^*C*^) and flow (*J*^*F*^) thresholds, we fit $\Delta {\rm{Re}}{\rho }_{yx}^{{\rm{THE}}}$] according to the following three phenomenological equations

7 $$\Delta {\rm{Re}}\left[{\rho }_{yx}^{{\rm{THE}}}\right]=a\left[\frac{1}{\sqrt{\pi }}{\int }_{0}^{\frac{J-b}{c}}{e}^{-{t}^{2}}dt+1\right],$$

8 $$\Delta {\rm{Re}}\left[{\rho }_{yx}^{{\rm{THE}}}\right]=\frac{2a}{1+{e}^{-\left(\frac{J-b}{c}\right)}}$$

9 $$\Delta {\rm{Re}}\left[{\rho }_{yx}^{{\rm{THE}}}\right]=2a{e}^{-{e}^{-}\left(\frac{J-b}{c}\right)}$$

where *a*,*b*, and *c* are fitting parameters. These equations provide a simple yet effective phenomenological approximation of the current density dependence of $\Delta {\rm{Re}}[{\rho }_{yx}^{{\rm{THE}}}]$ without complete knowledge of the underlying dynamics. In addition, the use of three different equations helps reduce the ambiguity in determining the threshold current densities that arises from the specific choice of fitting function. Each equation describes a variation in $\Delta {\rm{Re}}[{\rho }_{yx}^{{\rm{THE}}}]$ from zero in the pinned regime, through a crossover region (creep regime), to its maximal value in the flow regime. For each function, the creep threshold *J*^*C*^ is defined as the current density corresponding to the *x*-intercept of an extrapolated linear fit to the creep region. The flow threshold *J*^*F*^ is defined as the current density at which the difference between the fitted value of $\Delta {\rm{Re}}[{\rho }_{yx}^{{\rm{THE}}}]$ and its saturated value approaches a defined tolerance. Then, we calculate the average of all the values obtained from the three phenomenological equations while varying the tolerances between 0.05–1% and present them together with the standard error. More detailed discussion and an example of this procedure are provided in Supplementary Note 8 and Supplementary Figs. 8 and 9.

### Calculation of electron velocity

The electron velocity in Fig. 2c was calculated from the ordinary Hall coefficient *R*_0_ according to the classical Drude model such that

10 $${\rho }_{yx}^{{\rm{OHE}}}={R}_{H}B=\frac{B}{ne},$$

where *n* is the electron number density and *e* is the electron charge. *R*_*H*_ was obtained by fitting a straight line to the resistivity where ∣*B*∣ > 0.7 T. An example is shown in Supplementary Fig. 2.

## Supplementary information

Supplementary Information

Transparent Peer Review file

## Data Availability

All experimental data are available at Zenodo at 10.5281/zenodo.18413757 (ref. ^51^).

## Code availability

All Python code used for analysis and reproduction of all figures is available at Zenodo at 10.5281/zenodo.18413757 (ref. ^51^).
