# Supplementary material for: Emergent reactance induced by the deformation of a current-driven skyrmion lattice
Source: Nat Commun. 2026 Feb 19;17:2921. doi: 10.1038/s41467-026-69698-1 (PMC13031896; doi:10.1038/s41467-026-69698-1)
Supplement: Supplementary file 1 — Supplementary Information [file 41467_2026_69698_MOESM1_ESM.pdf]

Supplementary Information
Emergent reactance induced by the deformation of a current-driven skyrmion lattice

Matthew T. Littlehales,[1,2,7,*] Max T. Birch,[3] Akiko Kikkawa,[3] Yasujiro Taguchi,[3] Diego Alba Venero,[2] Peter D. Hatton,[1] Naoto Nagaosa,[3,4] Yoshinori Tokura,[3,5,6] and Tomoyuki Yokouchi[3,†]

[1]*Durham University, Department of Physics, South Road, Durham, DH1 3LE, United Kingdom*

[2]*ISIS Neutron and Muon Source, Rutherford Appleton Laboratory, Didcot, OX11 0QX, United Kingdom*

[3]*RIKEN Center for Emergent Matter Science (CEMS), Wako, Japan*

[4]*Fundamental Quantum Science Program (FQSP), TRIP Headquarters, RIKEN, Wako 351-0198, Japan*

[5]*Department of Applied Physics, University of Tokyo, Tokyo, Japan*

[6]*Tokyo College, University of Tokyo, Tokyo, Japan*

[7]*Present Address: Physik-Department, Technische Universität München (TUM), James-Franck-Str. 1, 85748 Garching, Germany*

[*] Corresponding author: matthew.littlehales@tum.de

[†] Corresponding author: tomoyuki.yokouchi@riken.jp

SUPPLEMENTARY NOTE 1: CORRECTION PROCEDURE FOR COMPLEX IMPEDANCE

The impedance measurements include both the resistance (R) and intrinsic inductance/capacitance ($X_{\mathrm{Intrinsic}}$) of our sample, as well as an extrinsic inductance/capacitance ($X_{\mathrm{Extrinsic}}$) arising from parasitic effects from the cables, connectors, and instruments, causing a systematic but frequency dependent rotation of the signal phase [1]. In addition, extrinsic effects such as parasitic inductance/capacitance and imperfections of the reference signal result in mixing of the true real and imaginary components of the complex impedance. Overall, the measured complex impedance can be expressed as

$$Z = e^{-i\phi}\left[R + X_{\mathrm{Intrinsic}} + X_{\mathrm{Extrinsic}}\right],$$

where R and ϕ are the sample resistance and the phase rotation, termed "trivial rotation", respectively. We correct these extrinsic effects as follows:

First, we correct for the trivial rotation. The effect of the trivial rotation can clearly be seen in Figs. S1a-d, which display the real parts of the longitudinal and Hall resistivities (Figs. S1a and b) and the raw data of the imaginary parts of the longitudinal and transverse reactance (Figs. S1c and d). It is evident that, without correction, the imaginary component takes on a similar form to the real part except for the prominent emergent reactance signals in the skyrmion phase. This similarity results from the mixing of resistance and reactance due to the trivial phase rotation. To correct for the trivial rotation, since the sample is not expected to exhibit any reactance in the field polarised (FP) region, we determine the trivial rotation from the FP region and apply the same rotation correction to the entire magnetic field range. As shown in Figs. S1e and f, after correcting for the trivial rotation, prominent signals still remain in the skyrmion phase.

Next, after correcting the trivial rotation, we separate the intrinsic contribution from the extrinsic reactance ($X_{\mathrm{Extrinsic}}$). The extrinsic reactance is symmetric against the magnetic field, and so for the Hall measurement, it is removed during the antisymmetrisation procedure. However, for the longitudinal component, the extrinsic contribution is not removed, since the signal itself is symmetric, and thus we must perform an additional extrinsic subtraction procedure to obtain the true magnitude of the signal arising from the skyrmions. Because the extrinsic reactance is independent of the magnetic field, we perform this subtraction by fitting a straight line to the field polarised region and then subtracting this from

the complete magnetic field sweep. The final corrected reactance signals are shown in Figs. S1g and h.

Our correction procedure is further validated by the following points. First, although the determination of the trivial rotation and extrinsic reactance was performed in the field polarized (FP) region, the corrected reactance is also zero in the conical and helical phases, in which the reactance is likewise expected to be zero. Second, when we perform the same correction procedure at 15 K, in which no skyrmion contribution is expected, the correction

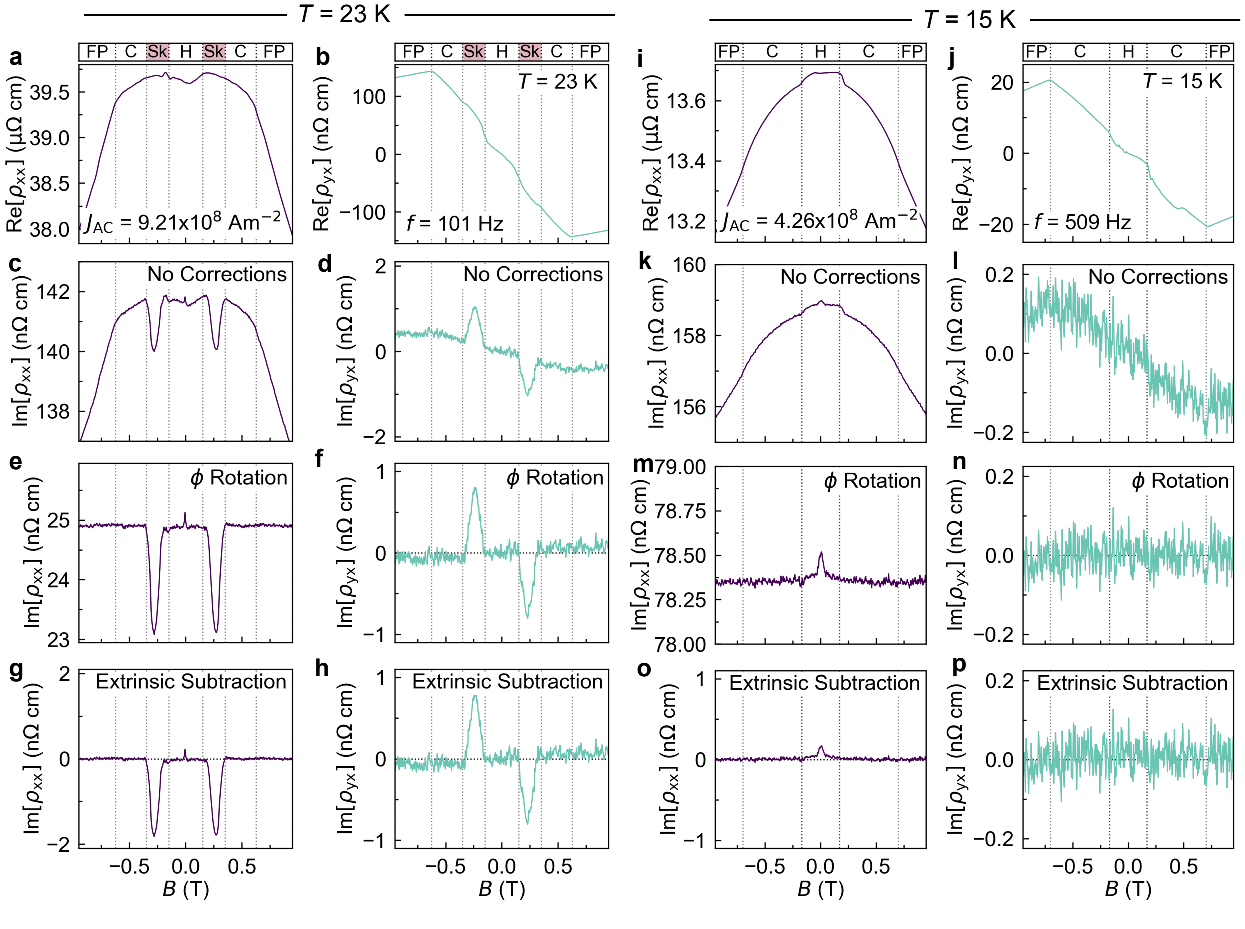

Fig. S1. **Correction Procedure for Complex impedance a-d** Magnetic field dependence of raw data of Re[ρ_{xx}] (**a**), Re[ρ_{yx}] (**b**), Im[ρ_{xx}] (**c**), and Im[ρ_{yx}] (**d**) measured at $f = 101\,\mathrm{Hz}$ and $J_{\mathrm{AC}} = 9.21\times10^{8}\,\mathrm{A\,m^{-2}}$ at $T = 23\,\mathrm{K}$. **e,f** Im[ρ_{xx}] and Im[ρ_{yx}] after correcting for the trivial phase rotation. **g,h** The final corrected Im[ρ_{xx}] and Im[ρ_{yx}], obtained by the subtraction of extrinsic reactance from the trivial-rotation-corrected reactance signals (shown in **e** and **f**). **i-p** Corresponding correction procedure at $T = 15\,\mathrm{K}$ with $f = 509\,\mathrm{Hz}$ and $J_{\mathrm{AC}} = 4.26\times10^{8}\,\mathrm{A\,m^{-2}}$.

yields no imaginary contributions (Figs. S1i-p), indicating that the finite signal in the skyrmion phase does not result from our correction procedure.

SUPPLEMENTARY NOTE 2: ESTIMATION OF TOPOLOGICAL HALL RESISTIVITY

For estimation of topological Hall resistivity, we first subtract the ordinary Hall effect (OHE) by subtracting a straight line fit to the field polarised phase as demonstrated in Fig. S2a. Next, two methods were used in estimating the THE; for varying temperatures, and constant temperatures. These methods are outlined below.

Varying temperature measurements:

Due to the differing phase boundaries and anomalous Hall effect (AHE) with changing temperature, we first estimate the THE contribution through a linear fitting process. Fig. S2b outlines the process in which we subtract fit a straight line to the conical regime. This analysis assumes that the anomalous Hall effect is proportional to the magnetic field. Strictly speaking, the anomalous Hall resistivity is proportional to the magnetization. However, since the magnetization is nearly linearly proportional to the magnetic field around the skyrmion phase in MnSi [2], this procedure provides a good approximation, serving as a useful method to estimate the THE when the magnetic phase boundaries vary with temperature, but it leads to discrepancies in the helical phase at low magnetic fields due to a different gradient, as demonstrated in Fig. S2c. This method was used to estimate the magnitude and extent of the THE contribution from the SkL in Fig. 1e of the main text.

Constant temperature measurements:

For the constant temperature measurements presented in Figures 2-4 in the main text, it is important to determine accurate changes in the THE. Consequently, we assume that both the SkL phase boundaries in magnetic field remained constant for varying current density and frequency, and that the AHE scales linearly through the SkL phase. Accordingly, we obtained the subtraction by first fitting a spline curve to the data that is linearly interpolated through the SkL phase and then subtracting this spline fit from the raw data. An example of this subtraction is presented in Fig. S2d, with the result of the subtraction shown in Fig. S2e. By definition, the helical, conical and field polarised phases therefore exhibit no THE contribution, and this method provides a more accurate determination of small changes to

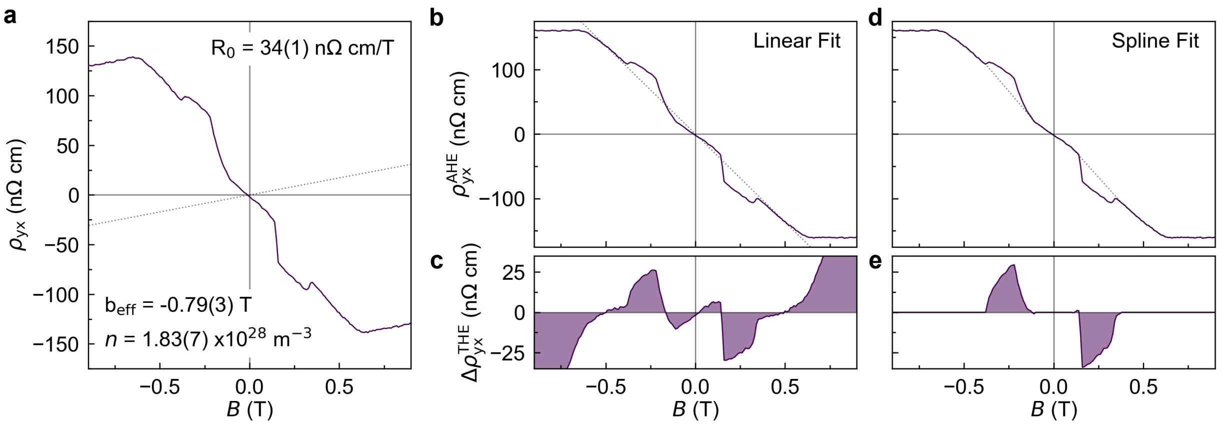

Fig. S2. **Ordinary Hall effect (OHE) measurements and anomalous Hall effect (AHE) subtractions.** **a** The Hall resistivity ρ_{yx} plotted as a function of magnetic field B measured at 23 K using J_{AC} =$1.74 \times 10^7\,\mathrm{A\,m^{-2}}$. Above saturation ($\sim$ 0.7 T), we assume that the signal is dominated by the ordinary Hall effect and fit a straight line to acquire the Hall coefficient R_{H}. **b**, **c** Linear fit of the Hall resistivity (**b**) and the difference between the fit and the measured data (**c**). **d**, **e** Spline fit of the Hall resistivity, assuming constant phase boundaries and linear interpolation through the SkL phase (**d**) and the difference between the fit and the measured data (**e**). The topological Hall resistivity at the center of the skyrmion phase $\rho_{yx}^{\mathrm{THE}}(B = 0.24\,\mathrm{T}) = -27.06\,\mathrm{n\Omega\,cm}$, from which we estimate the effective magnetic field to be $b_{\mathrm{eff}} = -0.79(3)\,\mathrm{T}$.

the THE. No appreciable differences to the interpretation of the main results are found with the two differing methods.

SUPPLEMENTARY NOTE 3: MOTION OF SKYRMION LATTICE DRIVEN BY DC CURRENT

Velocity of skyrmion lattice and electron

To investigate the skyrmion velocity in the flow regime, we have evaluated the skyrmion velocity at various magnetic fields. The ratio of the skyrmion velocity to the electron velocity (v_e) is shown in the upper panels of Figs. S3a-g. We find that the skyrmion velocity takes similar values in the range from $0.75v_\mathrm{e}$ to $0.80v_\mathrm{e}$, with an average of $0.78(3)v_\mathrm{e}$, indicating that the fitting used to estimate of the skyrmion lattice velocity is reliable. We note that under Galilean relativity, the skyrmion velocity is expected to approach the conduction electron velocity, namely $v_\mathrm{Sk} = v_\mathrm{e}$, ideally. However, because MnSi is a multi-band system, the electron velocity calculated using the Drude model only provides an estimation of the order of magnitude [3, 6].

Scaling behaviour

In Fig. S4, we show the skyrmion velocity estimated from the reduction of the topological Hall resistivity as a function of the current density for bulk MnSi [3] and our MnSi thin-plate device. While the critical current densities differ substantially, once the SkL enters the flow regime, for both datasets the skyrmion velocity follows the scaling according to $v_\mathrm{Sk} \propto J^{1.15}$ which agrees with the theoretical expectation of $v_\mathrm{Sk} \propto J$. This result indicates that the dynamical properties of our device are similar to those of MnSi single crystal, apart from the increase in the critical current densities. The higher critical current densities can be attributed to enhanced collective pinning, caused by an increased number of pinning sites introduced during the FIB fabrication process and confinement effects in microfabricated samples [4–6].

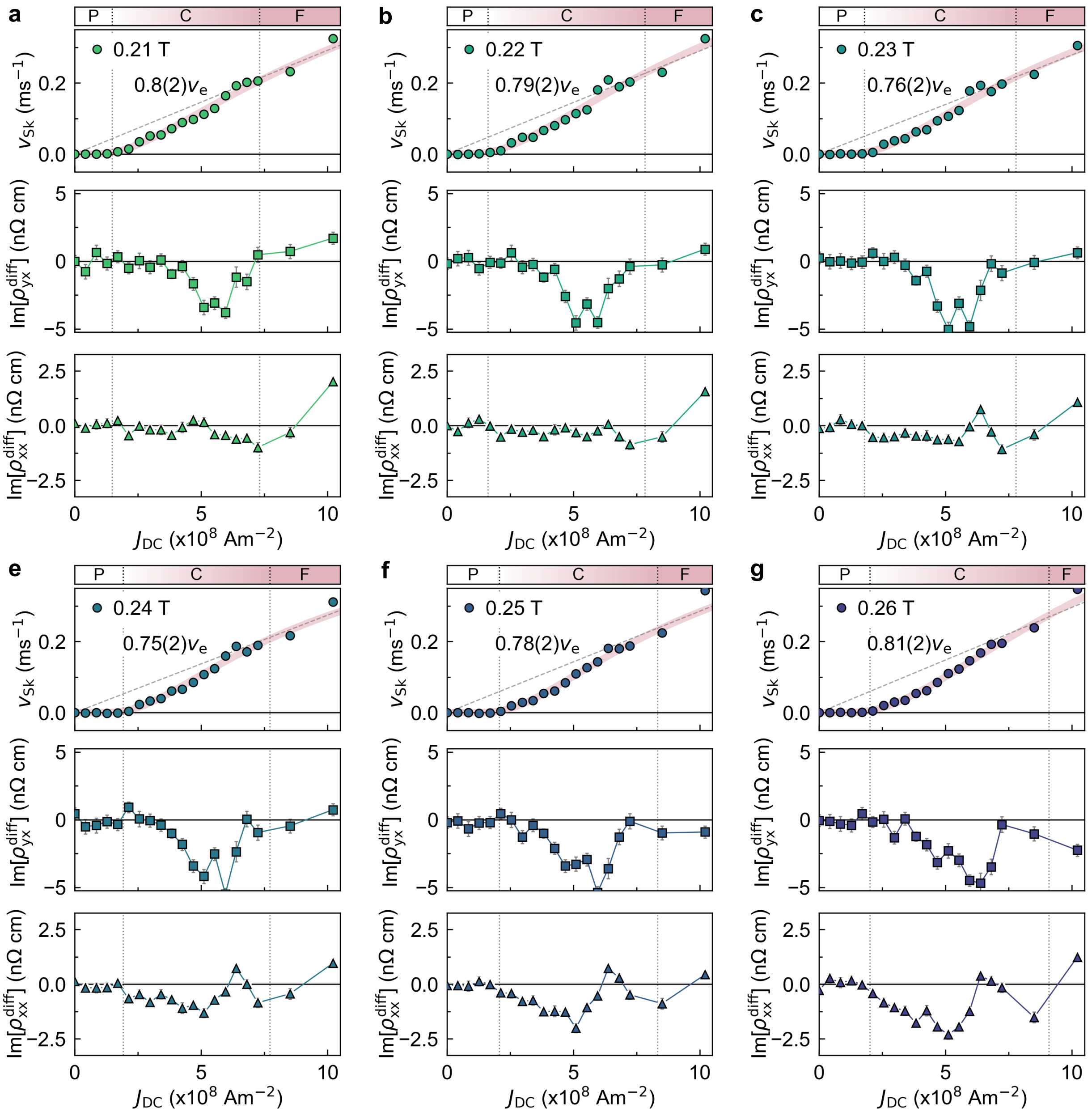

Fig. S3. J_{DC} **dependence of Im**[ρ_{yx}^{diff}] **and Im**[ρ_{xx}^{diff}] J_{DC} dependencies for v_{Sk} (top subplot) with linear fit to flow region and fraction of v_e indicated, Im[ρ_{yx}^{diff}] (middle subplot), and Im[ρ_{xx}^{diff}] (bottom subplot). Error bars correspond to the standard deviation of signal estimated in the field polarised regime, and where not visible, are smaller than the data points.

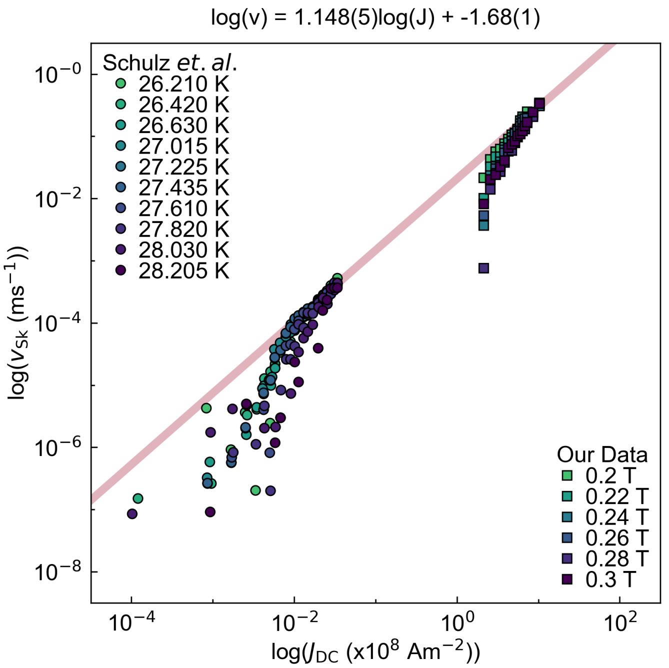

Fig. S4. **Scaling Comparison:** Scaling plot comparing the skyrmion velocity J_{DC} dependence of Schulz *et. al.,* (circles) at different temperatures [3], and our data (squares) for varying magnetic fields.

SUPPLEMENTARY NOTE 4: EMERGENT REACTANCE MEASUREMENTS USING DC BIAS CURRENT

The reactance signal is also observed in reactance measurements using a DC bias current. In Fig. S5 we show the raw data of the imaginary component of the differential complex impedance $\mathrm{Im}[\rho_{yx}^{\mathrm{diff}}]$ (Fig. S5a) and $\mathrm{Im}[\rho_{xx}^{\mathrm{diff}}]$ (Fig. S5b), as a function of the magnetic field for various DC bias current J_{DC}. It is clear for all J_{DC} that the signal is constrained to the SkL phase. In Fig. S3, we show v_{sk}, $\mathrm{Im}[\rho_{yx}^{\mathrm{diff}}]$, and $\mathrm{Im}[\rho_{xx}^{\mathrm{diff}}]$ for selected magnetic fields within the SkL phase. Qualitatively, the behavior is independent of the magnetic field, while the creep and flow thresholds vary slightly with B. In addition, the imaginary components in both the transverse and longitudinal complex impedance reach their maximum in the creep region, followed by a reduction in the flow region, consistent with the AC measurements shown in Fig. 3 in the main text. This highlights the notion that SkL dynamics in the creep region is responsible for the reactance [6].

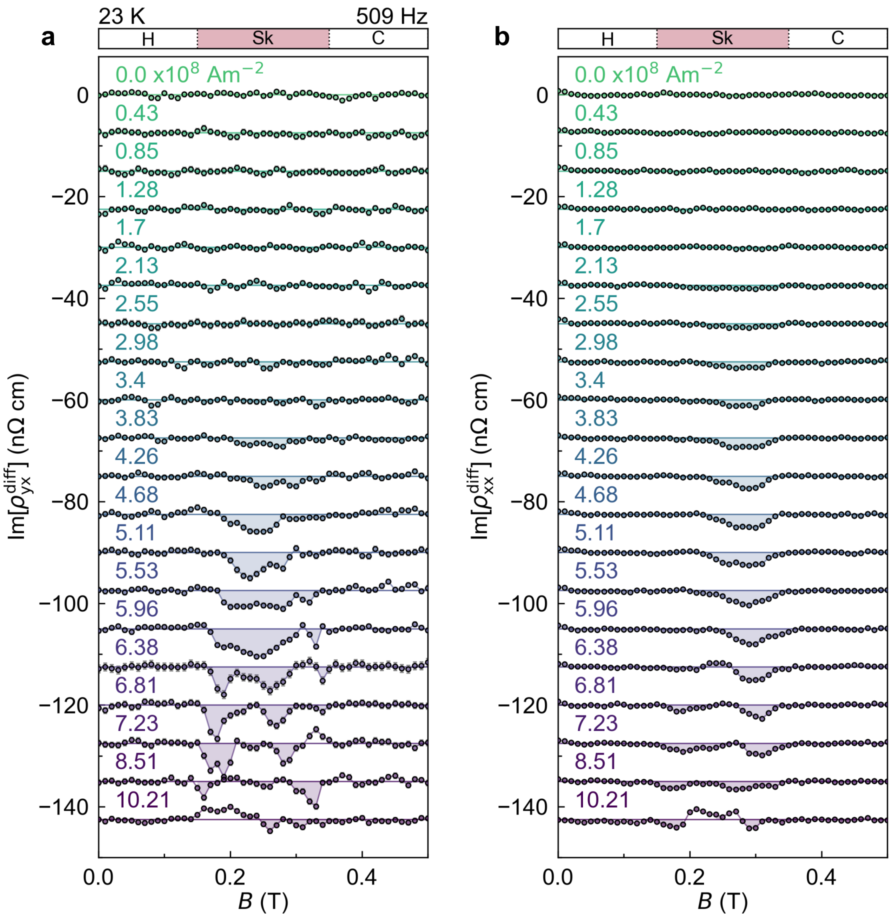

Fig. S5. **The imaginary part of the complex impedance in DC bias measurements.** Field dependence of $\mathrm{Im}[\rho_{yx}^{\mathrm{diff}}]$ (**a**) and $\mathrm{Im}[\rho_{xx}^{\mathrm{diff}}]$ (**b**) for increasing J_{DC}. The line plots are offset for clarity.

SUPPLEMENTARY NOTE 5: REACTANCE MEASUREMENT WITH AC CURRENT

In the AC+DC measurements, we apply a large DC current to drive skyrmions and superpose a small AC current for lock-in detection. In this case, only a single dynamical phase of the SkL (i.e., pin/creep/flow), determined by the magnitude of the DC current, contributes to the voltage response. In contrast, in the AC measurements, we apply a large AC current, which both drives skyrmions and is used for lock-in detection. In this case, over a full current cycle, the skyrmion velocity passes through multiple dynamical phases, all of which contribute to the measured voltage response, as discussed in Figs. 3a and b in the main text. Consequently, even when the amplitude of the AC current enters the flow region, the voltage response arising from the creep region still contributes to the measurement. This can explain the nonzero reactance signal observed in the flow regime.

To discuss this point in more detail, we calculate the current dependence of the reactance response measured with AC+DC current and with AC current using a toy model. In this calculation, we assume that the voltage is proportional to the time derivative of the current and that its coefficient depends on the current. Specifically, we adopt the following equation: $V = \left[e^{-(J+4)^2} + e^{-(J-4)^2}\right] dJ/dt$, in which the coefficient of dJ/dt peaks at $J = \pm 4$ and is nearly zero in both the low- and high-current regions (Fig. S6a). The current range where the coefficient is nonzero corresponds to the creep region in the experiment. Then, we calculate the time dependence of the voltage response for an AC+DC current $J = J_{\mathrm{DC}} + J_{\mathrm{AC}} \sin \omega t$ and an AC current $J = J_{\mathrm{AC}} \sin \omega t$ (Gray-dotted curves in Figs. S6b and c). For the AC+DC current, the voltage response is nonzero only when J_{DC} lies within the current range where the coefficient is nonzero (Fig. S6b). In contrast, for the AC current, even when the amplitude of the AC current exceeds this current range, a finite voltage signal appears (Fig.S6c) because the current passes through the low-current region where the coefficient is nonzero.

From these time dependences of the voltage, we calculate the current dependence of the real and imaginary parts of the first-harmonic voltage, which corresponds to what we measured in our experiment. As shown in Fig. S6d, for the AC+DC current, the current dependence of the imaginary component exhibits a peak structure around $J = 4$, similar to the current dependence of the coefficient of dJ/dt. In contrast, for the AC current, the peak

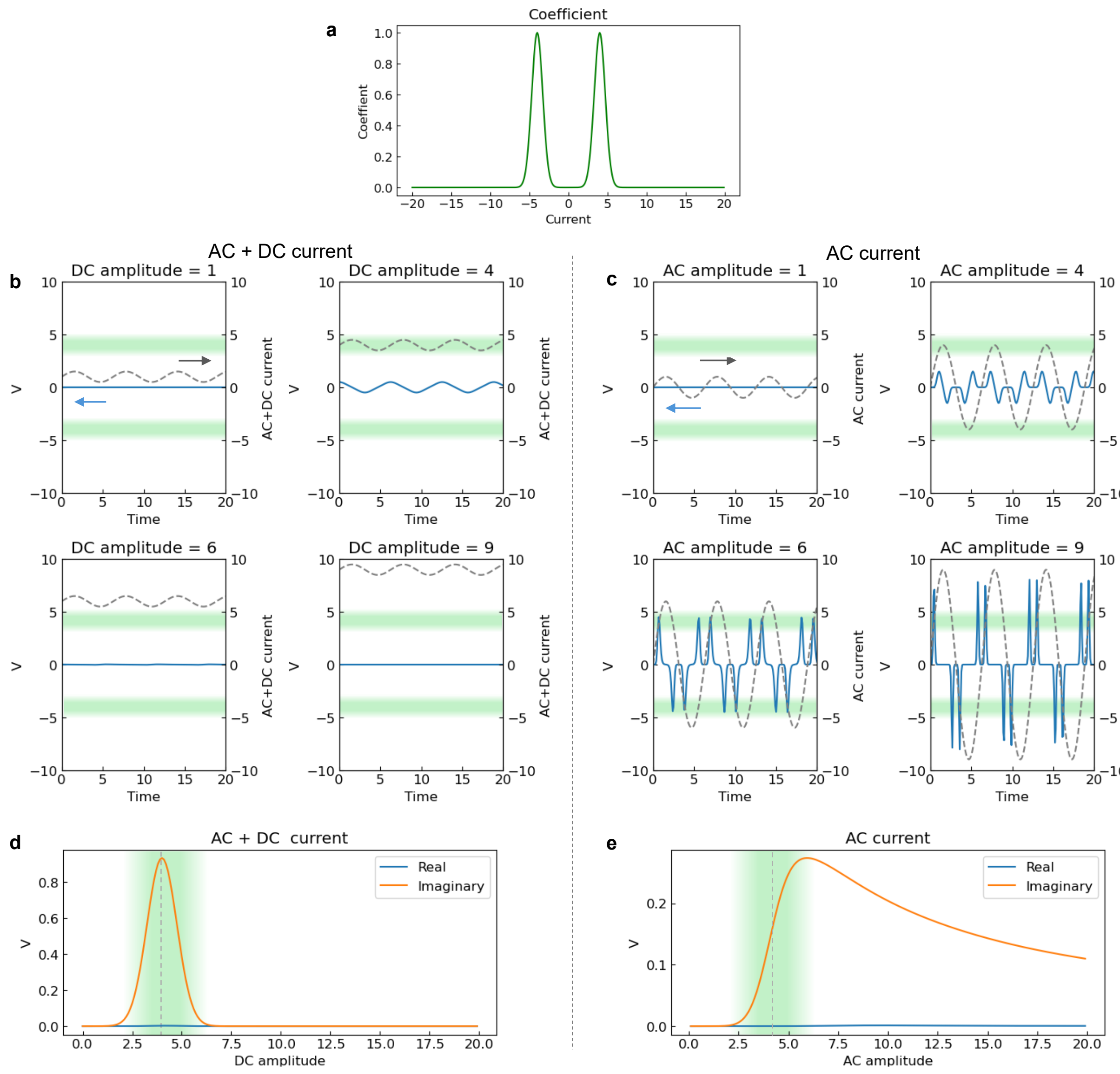

Fig. S6. **Calculation of the current dependence of the reactance signal.** **a** Current dependence of the coefficient of dJ/dt. **b-c** Time dependence of the applied currents (grey dotted curves) and the corresponding voltage responses (blue curves) for AC+DC current (**b**) and AC current (**c**). The green shaded region indicates the current range where the coefficient of dJ/dt is nonzero. **d-e** Current dependence of the real (blue) and imaginary (orange) parts of the first-harmonic voltage for AC+DC current (**d**) and AC current (**e**). The vertical grey dotted lines denotes the peak position of the coefficient of dJ/dt $(J = 4)$, and the green shaded region indicates the current range where the coefficient of dJ/dt is nonzero.

is smeared, and the signal persists even in the higher current region. This occurs because the current passes through the nonzero-coefficient region even at larger current amplitudes. The experimentally observed nonzero signal in the flow region is also explained by the same mechanism.

SUPPLEMENTARY NOTE 6: PHENOMENOLOGICAL CALCULATION OF EMERGENT REACTANCE IN SKYRMION LATTICE

Transverse reactance induced by inertial motion of skyrmion lattice

The velocity of the translational motion of the skyrmion v_{sk} obeys the Thiele equation:

$$m_{\mathrm{sk}}\dot{\boldsymbol{v}}_{\mathrm{sk}} + \mathcal{G} \times (\boldsymbol{v}_{\mathrm{e}} - \boldsymbol{v}_{\mathrm{sk}}) + \mathcal{D}(\beta \boldsymbol{v}_{\mathrm{e}} - \alpha \boldsymbol{v}_{\mathrm{sk}}) - \boldsymbol{\nabla} V_{\mathrm{pin}} = 0, \tag{S1}$$

where $\mathcal{G}$, $\mathcal{D}$, β, α, V_{pin}, and m_{sk} are the gyro-coupling vector, dissipative force tensor, dimensionless constant characterizing the nonadiabatic electron spin dynamics, Gilbert damping constant, pinning potential, and the skyrmion mass, respectively. Here, the skyrmion mass arises from a renormalization of the skyrmion deformation into a mass-like term in the Thiele equation. In the creep region, the pinning potential is anharmonic and exhibits multiple minima. Consequently, in the creep region, the time derivative of $\boldsymbol{v}_{\mathrm{Sk}}$ and the nonlinear term $\boldsymbol{\nabla} V_{\mathrm{pin}}$ in the Thiele equation cause the phase of $\boldsymbol{v}_{\mathrm{Sk}}$ to shift relative to that of the input AC current $\bar{\boldsymbol{J}}_{\mathrm{AC}} \sin(\omega t)$. Phenomenologically, the skyrmion velocity can be described by $\boldsymbol{v}_{\mathrm{sk}} = \boldsymbol{v}'_{\mathrm{sk}} \sin(\omega t) + \boldsymbol{v}''_{\mathrm{sk}} \cos(\omega t)$. In this case, the emergent electric field induced by the translational motion of SkL is given by $\boldsymbol{e}_{\mathrm{em}} = -\boldsymbol{v}_{\mathrm{Sk}} \times \boldsymbol{b}_{\mathrm{em}} = -\left[\boldsymbol{v}'_{\mathrm{sk}} \sin(\omega t) + \boldsymbol{v}''_{\mathrm{sk}} \cos(\omega t)\right] \times \boldsymbol{b}_{\mathrm{em}}$, which gives the transverse reactance component as $\mathrm{Im}[\rho_{yx}] = P b_{\mathrm{em}} v''_{\mathrm{sk}} / j_{\mathrm{AC}}$.

In contrast, in the flow region, since $m_{\mathrm{Sk}} = 0$ and $\boldsymbol{\nabla} V_{\mathrm{pin}} \sim 0$ due to the absence of the internal deformation and a relative reduction of pinning force, the skyrmion velocity is equal to the electron velocity. Therefore, the emergent electric field induced by the translational motion of SkL is given by $\boldsymbol{e}_{\mathrm{em}} = -\boldsymbol{v}_{\mathrm{Sk}} \times \boldsymbol{b}_{\mathrm{em}} = -\boldsymbol{v}_{\mathrm{e}} \times \boldsymbol{b}_{\mathrm{em}} \propto \boldsymbol{j}_{\mathrm{AC}} \sin(\omega t) \times \boldsymbol{b}_{\mathrm{em}}$, and the transverse reactance component disappears.

Next, we discuss the sign of the transverse reactance. In this Thiele equation, because V_{pin} is a nonlinear function, it is difficult to obtain a general solution for the skyrmion velocity. Nevertheless, to gain physical insight, we consider the case of $V_{\mathrm{pin}} \simeq 0$. Physically, this assumption means that the influence of the pinning potential becomes weak, although m_{sk} remains finite. This provides a good approximation of the dynamics in the creep region close to the flow threshold current. In this case, the Thiele equation can be solved, and the skyrmion velocity along the current direction (x direction) is given by

$$v^x_{\mathrm{sk}} = \frac{\alpha\beta D^2 + G^2 - i\omega m_{\mathrm{sk}} D\beta}{G^2 + (\alpha D)^2 - m_{\mathrm{sk}}^2\omega^2 - 2i\alpha D m_{\mathrm{sk}}\omega} v_e. \tag{S2}$$

This result explains the experimentally observed sign change of Imρ_{yx}: In the high frequency limit, the imaginary part of $v_{\rm sk}^x$ is given by $\mathrm{Im}[v_{\rm sk}^x] = \frac{D\beta}{\omega m_{\rm sk}} v_{\rm e}$ and is positive. In the low frequency limit $\omega \simeq 0$, the imaginary part of $v_{\rm sk}^x$ is given by

$$\mathrm{Im}[v_{\rm sk}^x] = \frac{D m_{\rm sk}}{G^2 + (\alpha D)^2} \left[\frac{2\alpha(D^2\alpha\beta + G^2)}{G^2 + (\alpha D)^2} - \beta \right] \omega v_{\rm e}. \tag{S3}$$

For example, for typical parameters in metals [$\alpha = 0.02, \beta = 0.05, D = 5.57\pi, G = 4\pi$ [4].], $\mathrm{Im}[v_{\rm sk}^x]$ is negative. In other words, in this case, the sign of $\mathrm{Im}[v_{\rm sk}^x]$ changes from negative to positive with increasing frequency, consistent with the experimentally observed sign change of $\mathrm{Im}[\rho_{yx}]$. We note that, for another parameter set, $\alpha = 0.04, \beta = 0, D = 5.57\pi, G = 4\pi$, $\mathrm{Im}[v_{\rm sk}^x]$ is positive. Thus, depending on the parameters, the low-frequency limit of $\mathrm{Im}[v_{\rm sk}^x]$ can take either positive or negative values.

Longitudinal emergent reactance induced by deformation of skyrmion lattice

Here, we discuss the relationship between the emergent electric field and phason and spin-tilting modes of SKL. The magnetic moment of the SkL can be described as a superposition of three helices including its deformation as follows:

$$\boldsymbol{m} = M_z \hat{\boldsymbol{z}} + \sum_{i=a,b,c} \boldsymbol{m}_i, \tag{S4}$$

$$\boldsymbol{m}_i = M_h(\beta_i \hat{\boldsymbol{Q}}_i + \sqrt{1-\beta_i^2}\boldsymbol{l}_i), \tag{S5}$$

$$\boldsymbol{l}_i = \hat{\boldsymbol{z}} \cos(\boldsymbol{Q}_i \cdot \boldsymbol{r} + \varphi_i) + (\hat{\boldsymbol{Q}}_i \times \hat{\boldsymbol{z}}) \sin(\boldsymbol{Q}_i \cdot \boldsymbol{r} + \varphi_i), \tag{S6}$$

where $\boldsymbol{Q}_i$ and $\hat{\boldsymbol{Q}}_i$ are the wavevector and it's unit vector for each helix. The three $\hat{\boldsymbol{Q}}_i$ vectors have a relative angle of 120 degrees. Here, φ_i and β_i are the phason and spin-tilting mode for each helix, respectively, which characterize the SkL deformation [7]. Generally, both φ_i and β_i are functions of time and space. As a simple example, we assume that one of the Q vectors is parallel to the current direction (x direction) and only the spin-tilting mode along the current direction is excited [i.e., $\beta_a = \beta_a(x, y, t)$ and $\beta_b = \beta_c = \varphi_a = \varphi_b = \varphi_c = 0$]. The emergent electric fields along x and y directions are given by

$$e_x = \frac{\hbar}{2e} \boldsymbol{n} \cdot (\partial_x \boldsymbol{n} \times \partial_t \boldsymbol{n}) \tag{S7}$$

$$e_y = \frac{\hbar}{2e} \boldsymbol{n} \cdot (\partial_y \boldsymbol{n} \times \partial_t \boldsymbol{n}), \tag{S8}$$

respectively. Here, $\boldsymbol{n}$ is the direction of the moment $\boldsymbol{n} = \boldsymbol{m}/|\boldsymbol{m}|$. Approximating $\beta_a \ll 1$ and $\boldsymbol{n} \approx \boldsymbol{m}$, we insert Eq. (3) into Eqs. (6) and (7) and obtain the following equatinos:

$$e_x = \frac{Q\hbar}{16e}\left[\cos\left(\frac{Qx}{2}\right)\cos\left(\frac{1}{2}\sqrt{3}Qy\right)\Big(\ 4M + 3\cos(Qx) + 15\ \Big) + 8M\cos(Qx) + 2\cos^3\left(\frac{Qx}{2}\right)\cos\left(\frac{1}{2}\sqrt{3}Qy\right) + 4\left(\cos\left(\sqrt{3}Qy\right) + 3\right)\right]\partial_t\beta_1(x,y,t)$$

$$e_y = \frac{\sqrt{3}Q\hbar}{4e}\left[2 - M + \cos(Qx)\right]\sin\left(\frac{Qx}{2}\right)\sin\left(\frac{\sqrt{3}Qy}{2}\right)\partial_t\beta_1(x,y,t).$$

Here, these electric fields have a spatially oscillating component and a spatially constant component. The observable electric fields are given by the spatial average as follows:

$$\langle e_x \rangle = \frac{3\hbar Q}{4e}\,\partial_t\beta_1(x,y,t) \tag{S9}$$

$$\langle e_y \rangle = 0 \tag{S10}$$

Therefore, when only the spin-tilting mode along the current direction is excited, the resulting emergent electric field appears only in the longitudinal direction and is proportional to the time derivative of the spin-tilting mode. This result is the same as in the case of the spin helix [8], apart from a constant factor. We note that, in the actual case, other phason and spin-tilting modes are also excited. In particular, in the case of helices, the spin-tilting and phason modes are responsible for the positive and negative emergent reactance, respectively [8, 9]. Since the sign of the emergent reactance in the SkL is negative, the phason mode of the SkL is expected to play an important role in the present case. However, further theoretical investigation remains a subject for future research.

For both traverse and longitudinal emergent reactance, a large signal is expected for a short period skyrmion lattice. The transverse emergent reactance depends on the emergent magnetic field, which becomes large for small skyrmions. In addition, according to Eq. (S9), the longitudinal emergent reactance is proportional to the wavevector of the skyrmion lattice (Q), indicating that smaller skyrmions yield larger longitudinal emergent reactance.

SUPPLEMENTARY NOTE 7: TEMPERATURE DEPENDENCE OF EMERGENT REACTANCE

In Fig. S7a-c we present the colour map of THE in the magnetic phase diagrams for three different current densities ($J_{\mathrm{AC}} = 0.68 \times 10^8\ \mathrm{A\,m^{-2}}$, $4.26 \times 10^8\ \mathrm{A\,m^{-2}}$, and $6.81 \times 10^8\ \mathrm{A\,m^{-2}}$).

The THE is calculated using the linear fit procedure described in Supplementary Note 2 (hence the discrepancies in the helical and conical phases), and phase boundaries are overlaid according to those calculated for Hall resistivity measured with $J_{\mathrm{AC}} = 4.26 \times 10^{8}\,\mathrm{A\,m^{-2}}$ using the method outlined in Methods. The THE exhibits distinct behaviour across three temperature ranges. As already shown in Fig. 2 of the main text, in the centre of the skyrmion phase (22-25 K), the THE decreases with increasing current density. This trend is also confirmed by the isothermal cuts at 23 K measured at each current density (Fig. S7d). The reduction of THE results from the current-induced motion of the SkL. In contrast, close to the paramagnetic transition temperature (25-28 K), THE does not depend on the current density as shown in Figs. S7a-c, and e. This behaviour suggests that the SkL remains pinned. Finally, at low temperatures (below 22 K), the THE measured at low current density is smaller than that observed at the center of the SkL phase, indicating that the skyrmion density is reduced due to its coexistence with the conical phase. Furthermore, with increasing current density, the THE increases, which can be ascribed to small Joule heating effects, and/or current induced skyrmion nucleation [10].

These results have some important implications for the interpretation of the temperature dependence of the emergent reactance. In correspondence with the THE, the emergent reactance also exhibits distinct behaviour across the three temperature ranges. In the centre of the skyrmion phase (22-25 K), both $\mathrm{Im}[\rho_{yx}]$ and $\mathrm{Im}[\rho_{xx}]$ show prominent signals. This temperature range corresponds to the range in which the SkL can move, as discussed above, supporting our main conclusion that the reactance originates from the SkL motion. In contrast, close to the paramagnetic transition temperature (25-28 K), a different behaviour is observed. First, $\mathrm{Im}[\rho_{yx}]$ falls below the noise level. As discussed above, since the SkL remains pinned in this temperature range, it is reasonable that the electric field arising from translational motion vanishes. Interestingly, in this temperature range, we find that $\mathrm{Im}[\rho_{xx}]$ is not zero but takes a small positive value. This positive $\mathrm{Im}[\rho_{xx}]$ may be interpreted as an emergent reactance arising from the current-induced deformation of pinned skyrmions, which is predicted to exhibit a positive emergent reactance [11]. Furthermore, this positive reactance decreases with increasing current density (Supplementary Figs. S7g and i), suggesting the skyrmions begin to be depinned at higher current densities, thereby reducing the positive reactance arising from the deformation of pinned skyrmions. Finally, at low temperatures (below 22 K) both $\mathrm{Im}[\rho_{yx}]$ and $\mathrm{Im}[\rho_{xx}]$ drop below the noise level. The absence

of the reactance signals at low temperatures is a consequence of the skyrmion metastability. Since metastable skyrmions are easily destroyed or created by current application as discussed above, the dynamical phase diagram differs from that in the centre of the skyrmion phase, in which emergent reactance is observed. In addition, the phason and spin-tilting modes are not well-defined for isolated metastable skyrmions. Therefore, only the equilibrium skyrmions lattice exhibits the expected dynamics discussed in the main text, and the emergent reactance.

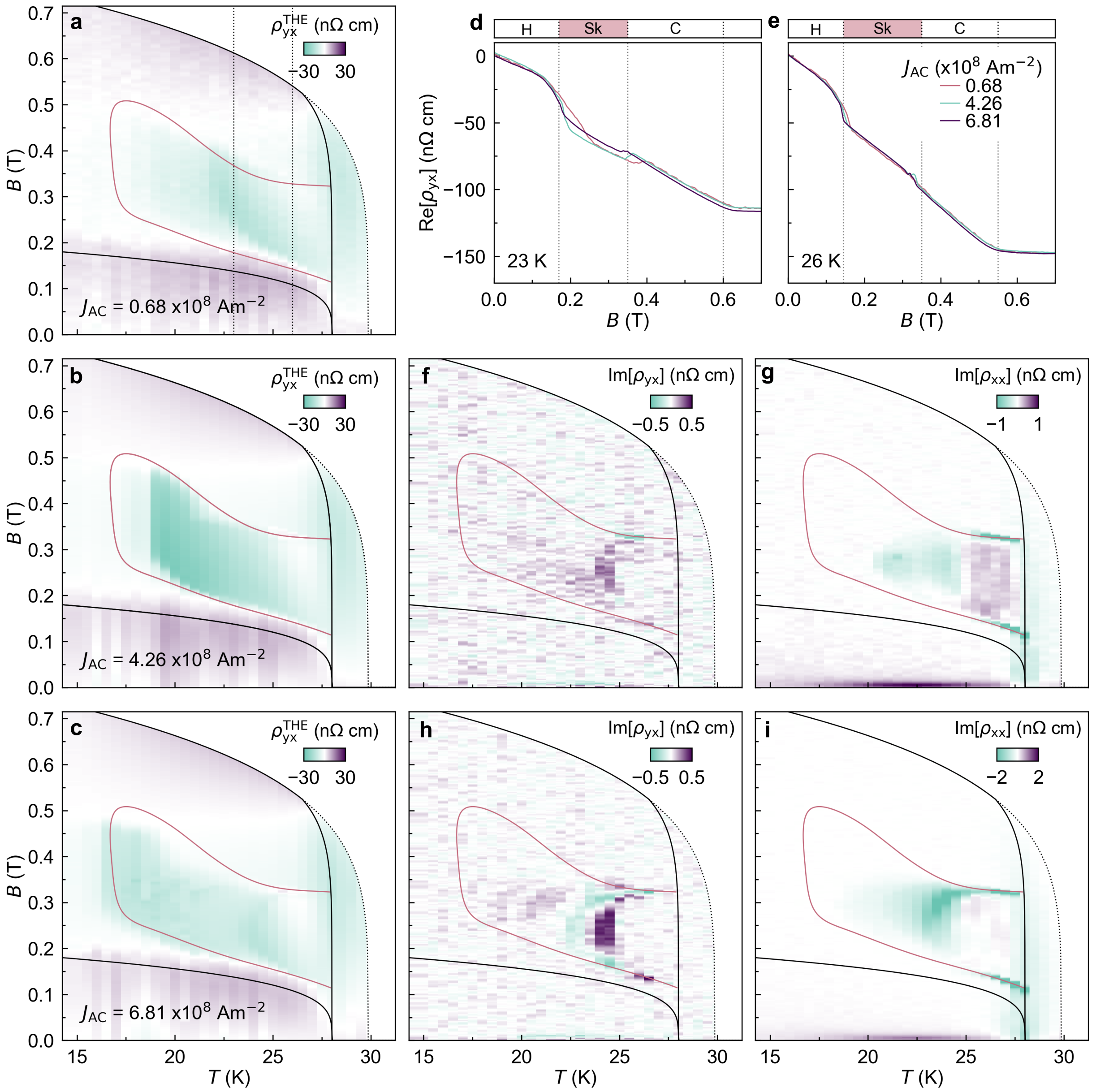

Fig. S7. **Demonstration of SkL pinning close to the transition. a-c** Re[ρ_{yx}^{THE}] for differing current densities: $J_{\mathrm{AC}} = 0.68 \times 10^8\,\mathrm{A\,m^{-2}}$, $4.26 \times 10^8\,\mathrm{A\,m^{-2}}$, and $6.81 \times 10^8\,\mathrm{A\,m^{-2}}$ respectively. **d**, **e** Magnetic field dependence of Re[ρ_{yx}^{THE}] for each of the three current densities at 23 K (**d**) and 26 K (**e**), clearly in (**e**) there is no change to the THE, indicating the skyrmions are pinned. **f**, **g** $B - T$ color maps of Im[ρ_{yx}] and Im[ρ_{xx}] measured with $4.26 \times 10^8\,\mathrm{A\,m^{-2}}$. **h**, **i** Identical plots for J_{AC} = $6.81 \times 10^8\,\mathrm{A\,m^{-2}}$. Notably, in (**g**), a positive Im[ρ_{xx}] arises close to the fluctuation disordered regime. Since the SkL is pinned in this regime, we attribute this signal to the deformation of the pinned SkL [11].

SUPPLEMENTARY NOTE 8: DETERMINATION OF THRESHOLD CURRENT DENSITIES

As described in the Methods in the main text, the definitions of the pinned, creep, and flow regions are somewhat difficult to define, since their boundaries typically appear as smeared crossovers caused by thermally activated processes rather than sharp transitions. It is therefore appropriate to define criteria based on physical considerations for the pinned-to-creep (P/C) and creep-to-flow (C/F) thresholds, which are then determined through a phenomenological fitting procedure. The threshold current densities are reasonably defined as follows: In the flow region, skyrmions catch up with the conduction electron velocity, leading to the saturation of $\Delta\mathrm{Re}[\rho_{yx}^{\mathrm{THE}}]$. Therefore, the flow region should be defined as the region where $\Delta\mathrm{Re}[\rho_{yx}^{\mathrm{THE}}]$ becomes saturated. Consequently, we define the C/F threshold as the point at which the difference between the fitted value of $\Delta\mathrm{Re}[\rho_{yx}^{\mathrm{THE}}]$ and its saturated value approaches a defined tolerance. In the pinned regime, $\Delta\mathrm{Re}[\rho_{yx}^{\mathrm{THE}}] = 0$, and so the P/C threshold should be defined as the point at which $\Delta\mathrm{Re}[\rho_{yx}^{\mathrm{THE}}]$ deviates from zero. This boundary is strongly smeared due to thermal activation, and so if we were to define the P/C threshold from the current density at which $\Delta\mathrm{Re}[\rho_{yx}^{\mathrm{THE}}]$ appears to take a finite value, similar to the definition of the C/F threshold, it would be strongly affected by the measurement accuracy of $\Delta\mathrm{Re}[\rho_{yx}^{\mathrm{THE}}]$, as discussed in [12]. To avoid such ambiguity, we define the P/C threshold in practice as the current density corresponding to the x-intercept of an extrapolated linear slope that is fit to the creep region.

To systematically and precisely determine the P/C and C/F thresholds based on the above definitions, we fit $\Delta\mathrm{Re}[\rho_{yx}^{\mathrm{THE}}]$ with phenomenological equations. Here, we use three functions: the error function, the sigmoid function, and the Gompertz function [Eqs. (7)-(9) in the main text]. The use of three different equations helps reduce the ambiguity in determining the threshold current densities that arises from the specific choice of fitting function. Each equation is an S-shaped curve, in which we can approximately define three regimes according to the criteria defined above: (1) a pinned region, where $\Delta\mathrm{Re}[\rho_{yx}^{\mathrm{THE}}]$ is zero, (2) a creep region, where $\Delta\mathrm{Re}[\rho_{yx}^{\mathrm{THE}}]$ increases with current, and (3) a flow region, where $\Delta\mathrm{Re}[\rho_{yx}^{\mathrm{THE}}]$ is saturated. In Fig. S8, we show fits to the error function (Eq. (7)) as an example, which closely reproduces the experimentally observed current dependence of $\Delta\mathrm{Re}[\rho_{yx}^{\mathrm{THE}}]$. For this representative example, the gray dashed lines indicates the P/C

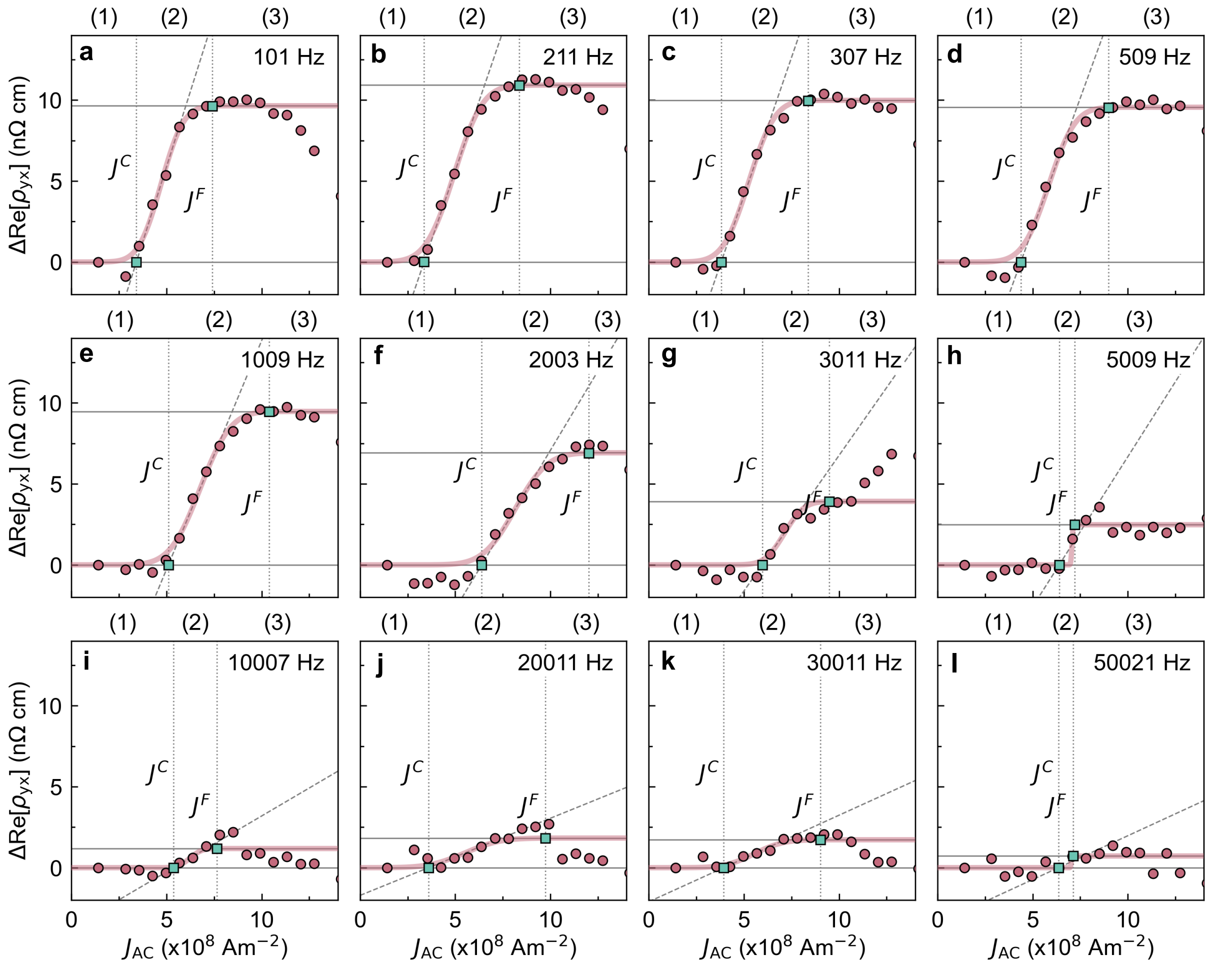

Fig. S8. **Determination of threshold current densities a-l**. Current density dependence of $\Delta\mathrm{Re}[\rho_{yx}^{\mathrm{THE}}]$ at 23 K, 0.24 T, and various frequencies. The red circles represents the experimental data, and the solid red curves are fit to Eq. (7). The green squares and vertical dotted lines denote the threshold current densities. Labels (1), (2), and (3) indicate the pinned, creep, and flow regimes, respectively. Error bars correspond to the standard deviation of signal estimated in the field polarised regime, and where not visible, are smaller than the data points.

threshold (J^C) and C/F threshold (J^F) with a tolerance of 0.1%.

We performed this analysis for each of the three functions while varying the tolerance for the C/F threshold within a range from 0.05% to 1%. These results for the data set shown in Fig. 3 of the main text are shown in Fig. S9. In Figs. S9a-c, we show the P/C thresholds with dotted lines, and C/F thresholds with dashed lines, for tolerances of 0.05%, 0.1% and 0.5%. The P/C thresholds are almost the same for all three functions, as also confirmed

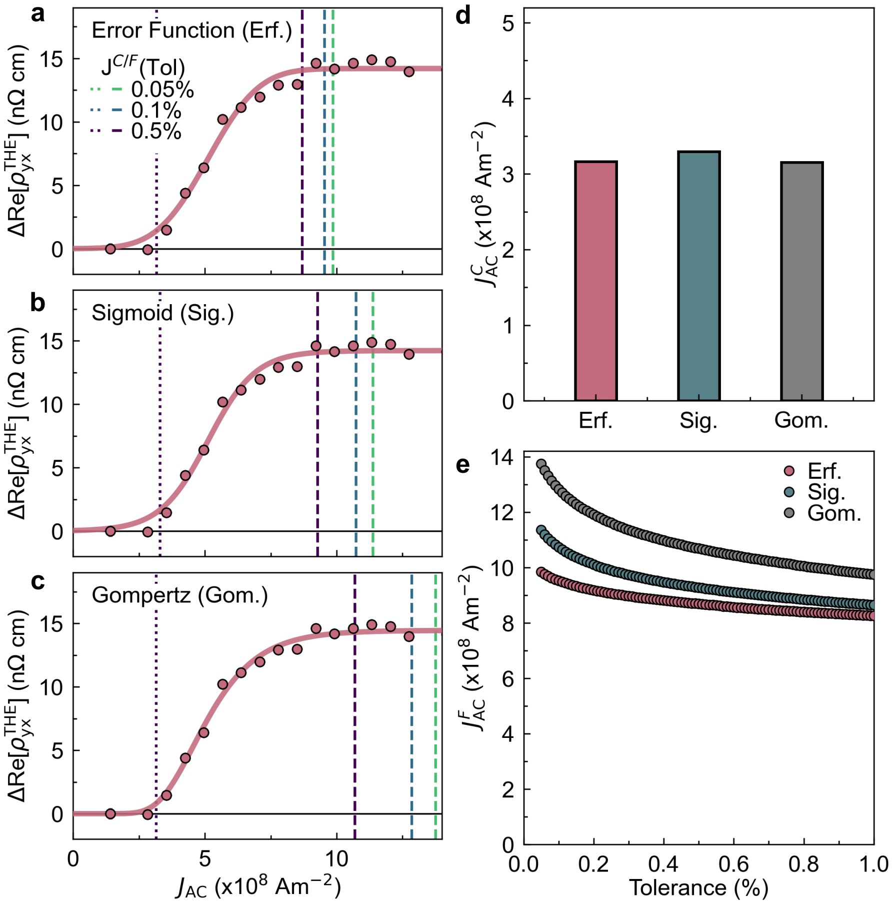

Fig. S9. **Fitting function and tolerance comparison: a-c** Data taken from Fig. 3 of main text and fit with equations (7) (Error function (Erf.)), (8) (Sigmoid (Sig.)), and (9) (Gompertz (Gom.)) as defined in the main text. **d** P/C thresholds determined by using each function. **e** Tolerance dependence on the C/F threshold for each function.

by the bar chart in Fig. S9d (Note that the P/C transition is, by definition, independent of the tolerance). For all tolerances between 0.05-1%, the C/F thresholds are plotted in Fig. S9e. Clearly, the specific choice of fitting function form and tolerance does not lead to a meaningfully different value of the of P/C threshold; the defined P/C threshold well represents the current density at which $\Delta\text{Re}[\rho_{yx}^{\text{THE}}]$ reaches its saturation value. Then, to properly indicate the ambiguity in determining the thresholds, we calculate the average of all the values obtained from the three phenomenological equations while varying the tolerances between 0.05-1% and present them together with the standard error.

SUPPLEMENTARY NOTE 9: JOULE HEATING CONSIDERATIONS

Here, we discuss the possible effects of Joule heating in our measurements and demonstrate that they cannot be responsible for the effects observed in this work.

Time-varying Joule Heating Effect

Theoretical models suggest that a time-varying resistance change due to the time-varying Joule heating effect may produce a non-linear reactance [13]. The main claim of this theory is that the imaginary part of the complex resistivity $\mathrm{Im}[\rho]$ arises from a delayed response of the time-varying resistance change as a consequence of the delay in the thermal relaxation process. In this model, $\mathrm{Im}[\rho]$ is proportional to the temperature derivative of the resistivity i.e. $\frac{d\mathrm{Re}[\rho]}{dT}$. Thus, a useful test to determine whether time-varying Joule heating is responsible is to compare the magnetic-field dependence of $\frac{d\mathrm{Re}[\rho]}{dT}$ and $\mathrm{Im}[\rho]$. We present $\frac{d\mathrm{Re}[\rho]}{dT}$ and $\mathrm{Im}[\rho]$ for both the Hall and longitudinal resistivities in Fig. S10 . Here, the temperature derivative of the resistivity is approximated by the difference in resistivity between 23 K and 23.25 K. It is clear that the magnetic-field dependence of $\mathrm{Im}[\rho]$ does not resemble that of $-\frac{d\mathrm{Re}[\rho]}{dT}$. Therefore, we conclude that time-varying Joule heating cannot account for the observed effects.

Estimation of Joule heating due to DC bias current

In the case of AC+DC measurements, the DC bias current J_{DC} may induce a constant temperature increase, which could reduce the topological Hall effect. To confirm that this temperature change cannot account for the observed reduction in THE, we estimate the temperature increase when the heat bath (i.e., the temperature of the sample holder) is set to 23 K. We assume that the resistivity change under the application of J_{DC}, relative to that measured at $J_{\mathrm{DC}} = 0\,\mathrm{A\,m^{-2}}$, is caused by the temperature increase due to the Joule heating, and we calculate the corresponding temperature increases. In Fig. S11b, we show the estimated temperature increase as a function of J_{DC}. Our estimation is consistent with a previous study of MnSi thin-plate device, which reports a temperature increase of 0.2-0.3 K for $J_{\mathrm{DC}} = 7.7 \times 10^{8}\,\mathrm{A\,m^{-2}}$ [14]. For $J_{\mathrm{DC}} = 10 \times 10^{8}\,\mathrm{A\,m^{-2}}$, the maximum current density in this study, the temperature increase is estimated to be $\Delta T \approx 0.8\,\mathrm{K}$, which would lead

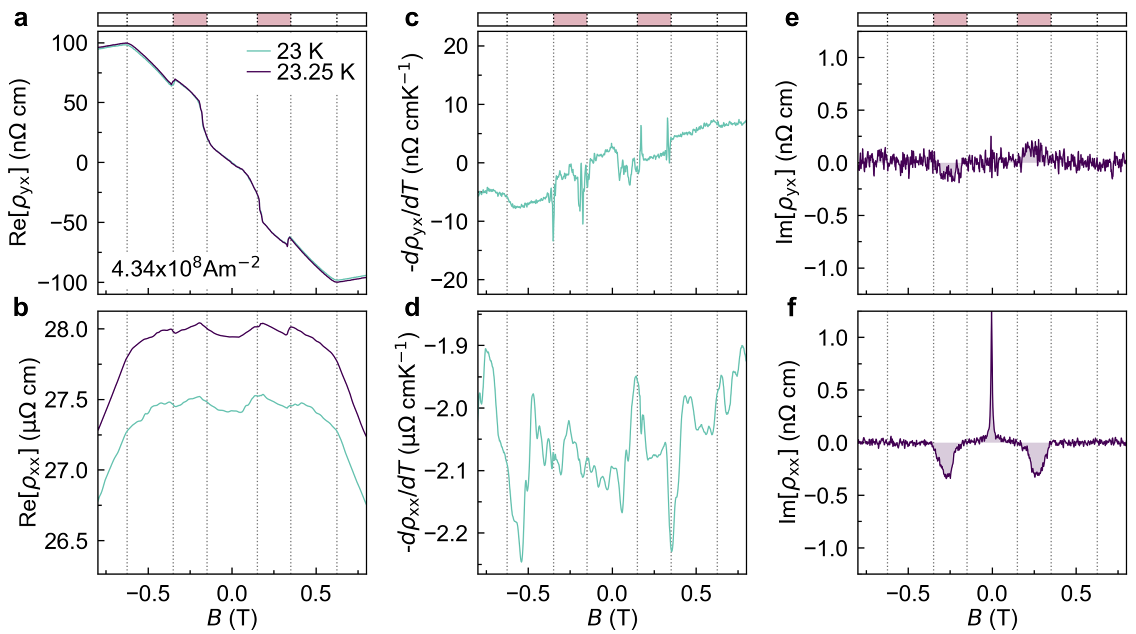

Fig. S10. **Time-varying Joule Heating Effect.** **a**, **b** Magnetic field dependence of the Hall resistivity Re[ρ_{yx}] (**a**) and magneto-resistivity Re[ρ_{xx}] (**b**) measured using PPMS at heat bath temperatures of 23 K (teal), and 23.25 K (purple). **c**, **d** $-\frac{d\rho_{yx}}{dT}$ and $-\frac{d\rho_{xx}}{dT}$ calculated from the magnetic field dependences in (**a**, **b**), respectively. **e**, **f** Magnetic field dependence of Hall reactance Im[ρ_{yx}] (**e**) and longitudinal reactance Im[ρ_{xx}] (**f**).

to a change in THE of $\Delta\rho_{yx}^{\mathrm{THE}} \approx 3.77\,\mathrm{n}\Omega\,\mathrm{cm}$, comparable to the noise level in the AC + DC measurement. Therefore, the observed reduction in THE $\Delta\rho_{yx}^{\mathrm{THE}} \approx 20\,\mathrm{n}\Omega\,\mathrm{cm}$ cannot be attributed to the Joule heating effects. We thus conclude that the change in THE observed in our AC+DC measurements originates from the emergent electric field induced by the translational motion of the SkL.

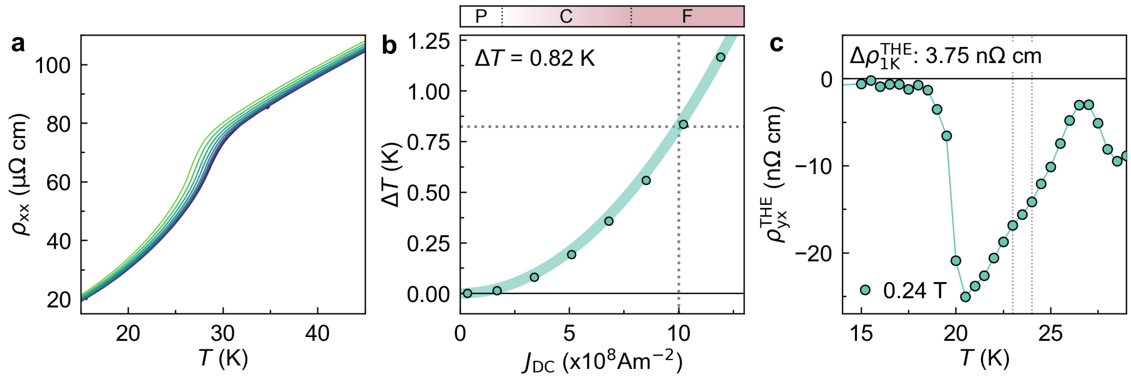

Fig. S11. **Estimation of Joule heating due to DC bias current.** **a** longitudinal resistivity ρ_{xx} as a function of temperature for various DC bias currents. **b** Temperature increase caused by the DC bias current when the heat bath of PPMS is set to $T = 23\,\mathrm{K}$. For $J_{\mathrm{DC}} = 10 \times 10^8\,\mathrm{A\,m^{-2}}$, the temperature change is estimated to be $\Delta T = 0.8\,\mathrm{K}$. **c** Topological Hall resistivity as a function of versus temperature at $B = 0.24\,\mathrm{T}$. The change in THE between 23 K and 24 K is approximately $\Delta\rho_{yx}^{\mathrm{THE}} = 3.75\,\mathrm{n\Omega\,cm}$.

[1] Choi, J., Lee, G.-H., Yang, J., Kang, J., Park, B.-G., Park, A. M. G. & Kim, K.-J. Questioning the validity of spintronic inductors: Potential artifacts in emergent inductance. *Applied Physics Letters* **125**, 192403 (2024).

[2] Bauer, A. & Pfleiderer, C. Magnetic phase diagram of MnSi inferred from magnetization and AC susceptibility. *Physical Review B* **85**, 214418 (2012).

[3] Schulz, T., Ritz, R., Bauer, A., Halder, M., Wagner, M., Franz, C., Pfleiderer, C., Everschor, K., Garst, M. & Rosch, A. Emergent electrodynamics of skyrmions in a chiral magnet. *Nature Physics* **8**, 301–304 (2012).

[4] Iwasaki, J., Mochizuki, M. & Nagaosa, N. Universal current-velocity relation of skyrmion motion in chiral magnets. *Nature Communications* **4**, 1463 (2013).

[5] Iwasaki, J., Mochizuki, M. & Nagaosa, N. Current-induced skyrmion dynamics in constricted geometries. *Nature Nanotechnology* **8**, 742–747 (2013).

[6] Birch, M. T., Belopolski, I., Fujishiro, Y., Kawamura, M., Kikkawa, A., Taguchi, Y., Hirschberger, M., Nagaosa, N. & Tokura, Y. Dynamic transition and galilean relativity of

current-driven skyrmions. *Nature* **633**, 554–559 (2024).

[7] Tatara, G. & Fukuyama, H. Phasons and excitations in skyrmion lattice. *Journal of the Physical Society of Japan* **83**, 104711 (2014).

[8] Nagaosa, N. Emergent inductor by spiral magnets. *Japanese Journal of Applied Physics* **58**, 120909 (2019).

[9] Kurebayashi, D. & Nagaosa, N. Electromagnetic response in spiral magnets and emergent inductance. *Communications Physics* **4**, 260 (2021).

[10] Lemesh, I., Litzius, K., Böttcher, M., Bassirian, P., Kerber, N., Heinze, D., Zázvorka, J., Büttner, F., Caretta, L., Mann, M., Weigand, M., Finizio, S., Raabe, J., Im, M.-Y., Stoll, H., Schütz, G., Dupé, B., Kläui, M. & Beach, G. S. D. Current-induced skyrmion generation through morphological thermal transitions in chiral ferromagnetic heterostructures. *Advanced Materials* **30**, 1805461 (2018).

[11] Furuta, S., Koshibae, W. & Kagawa, F. Symmetry of the emergent inductance tensor exhibited by magnetic textures. *npj Spintronics* **1**, 3 (2023).

[12] Kagawa, F., Oike, H., Koshibae, W., Kikkawa, A., Okamura, Y., Taguchi, Y., Nagaosa, N. & Tokura, Y. Current-induced viscoelastic topological unwinding of metastable skyrmion strings. *Nature Communications* **8**, 1332 (2017).

[13] Furuta, S., Koshibae, W., Matsuura, K., Abe, N., Wang, F., Zhou, S., Arima, T.-h. & Kagawa, F. Reconsidering nonlinear emergent inductance: Time-varying joule heating and its impact on AC electrical response. *Physical Review B* **110**, 174402 (2024).

[14] Sato, T., Koshibae, W., Kikkawa, A., Taguchi, Y., Nagaosa, N., Tokura, Y. & Kagawa, F. Nonthermal current-induced transition from skyrmion lattice to nontopological magnetic phase in spatially confined MnSi. *Physical Review B* **106**, 144425 (2022).
