# Supplementary material for: Emergent reactance induced by the deformation of a current-driven skyrmion lattice
Source: Nat Commun. 2026 Feb 19;17:2921. doi: 10.1038/s41467-026-69698-1 (PMC13031896; doi:10.1038/s41467-026-69698-1)
Supplement: Supplementary file 2 — Transparent Peer Review file [file 41467_2026_69698_MOESM2_ESM.pdf]

nature portfolio

Peer Review File

Emergent reactance induced by the deformation of a current-driven skyrmion lattice

Corresponding Author: Dr Matthew Littlehales

This file contains all reviewer reports in order by version, followed by all author rebuttals in order by version.

Version 0:

Reviewer comments:

Reviewer #1

(Remarks to the Author)
In this manuscript, the authors unveil the emergent reactance induced by current-driven skyrmion lattice dynamics. By measuring the ac electrical resistivity in a typical chiral magnet MnSi device, the authors found the existence of emergent reactance within the skyrmion crystal phase. Furthermore, it is found that the reactance is most profound within the creep regime in the dynamical phase of the skyrmion crystal. In addition to the expected transverse emergent reactance, which can be attributed to the skyrmion crystal deformation and the corresponding emergent electric field, a longitudinal emergent reactance is also identified. The origin of this longitudinal emergent reactance could be due to the phason and spin-tilting modes of the skyrmion crystal (as it is a superposition of three spin helices) as the authors discussed.
This work provides solid evidence of the emergent reactance originating from the dynamics of a skyrmion crystal. The identification of emergent reactance would not only shed light on the understanding of skyrmion crystal dynamics but could also be harnessed for potential applications. I believe this manuscript will be of interest to the physics and engineering community. Before I can make a recommendation of the manuscript, I have a few concerns that I would like the authors to address.

1. I am confused with the sign of the imaginary resistivities [\rho_xy and \rho_xx]. Why is \rho_xy odd and \rho_xx even with respect to the magnetic field? From my understanding, they are all originate from the emergent electric field [Eq. (1)], i.e., the spin distribution and the spin dynamics. The emergent electric field preserves the time reversal symmetry. The authors also give an expression of the transverse resistivity Im\rho_xy in the discussion, which also preserves the time-reversal symmetry. Then the symmetry of \rho_xy and \rho_xx with respect to the magnetic field should be the same. How to understand these quantities from a symmetry point of view?
2. In the pinned regime, the skyrmion would also deform [e.g., as shown in PNAS 119, e2200958119 (2022)]. Why cannot such deformation induce an emergent reactance? Related to this point, how do the authors rule out the possibility of a longitudinal emergent electric field generated by a deforming skyrmion crystal?
3. What determines the amplitude of the emergent reactance? I guess it may be related to the strength of the emergent magnetic field. Also do the authors expect that such emergent reactance can be further enhanced in spin textures beyond two dimensions like skyrmion bundles [e.g., Nat. Nanotechn. 16, 1086 (2021)], hedgehogs [Nat. Commun. 10, 1059 (2019)], and hopfions [e.g., Phys. Rev. Lett. 129, 267201 (2022)]? A related discussion could be given for future research directions.

Reviewer #2

(Remarks to the Author)
The manuscript reports on measurements of the complex reactance in a MnSi device, in order to determine contributions from the skyrmion phase. This is a valid goal as most information about skyrmion dynamics comes from direct imaging techniques such as LTEM. Developing complementary techniques based on transport measurements is therefore very attractive to advancing the field.

Over all the manuscript is quite well written, and the measurements themselves appear to be sound. My concern is the substantial amount of data processing necessary to bring out the features attributed to the skyrmion creep. This requires a very high degree of confidence in all the steps of the analysis, and in this regard, I am afraid the manuscript falls short (for example there is not a single error bar shown on any of the measurements or derived quantities!). I am also not sure that

attributing the results to the skyrmions is as definitive as the authors suggest. In particular I am puzzled why the signal is mostly seen in a very narrow range of temperatures of 24-25 K? While the absence of a signal at higher temperature may be due an increase in. the critical current as stated by the authors, why is it also absent a lower temperature? In brief, I am sure the authors measure *something*, I'm just not convinced by the explanation. Given this I am hesitant to recommend publication in the current form.

Some more specific comments are (in no particular order):
1) The claim that the observed reactance could be useful for applications seems far-fetched given how subtle the effect, requiring substantial subtractions of other contributions to the measured signal.
2) In discussing Fig. 2c it is stated that the skyrmion velocity shows a difference between the creep and flow states. This is not merited. Only a single data point was measured in the flow state. Also, it is not accurate to say the velocity is close to that of the electrons when the dashed lines show 0.75 v_e.
3) The discussion of Fig. 4, why the imaginary part of rho_xy changes sign and why the maxima cross the phase boundaries is handwaving and thus not very compelling.
4) What is the justification for Eq. (7)?
5) The criteria for determining the C/F transition in Fig. S7 seems arbitrary. Why not use the extrapolation to the saturations value to be consistent with the criteria for P/C?

Reviewer #3

(Remarks to the Author)
The manuscript presents an elegant and timely study of emergent reactance in a current-driven skyrmion lattice (SkL) in MnSi. By probing both the longitudinal and transverse components of the complex resistivity under AC excitation, the authors provide compelling evidence that internal skyrmion deformation plays a key role in emergent electromagnetic responses during the creep regime. The experimental results are significant for both fundamental understanding and potential device applications involving skyrmions in spintronic circuits.

The work is well-motivated and clearly written, and the data are thoughtfully analyzed. The findings will be of broad interest to the communities working on topological magnetism, emergent electrodynamics, and magneto-transport. I recommend publication after the following points are addressed:

1. The manuscript presents the skyrmion motion in terms of three regimes: pinned, creep, and flow. However, these transitions are not sharp at finite temperature and are more accurately described as crossovers. It would improve the clarity and rigor of the paper to more explicitly define the criteria used to distinguish these regimes in both DC and AC measurements. For example, in Fig. 2 and Fig. 3, how is the onset of creep (J_C) and the beginning of flow (J_F) quantitatively determined? Are these thresholds extracted from fitting procedures or based on inflection points? A discussion acknowledging the crossover nature of these regimes, particularly at finite temperature, would make the interpretation more robust.

2. In Fig. 4, both the creep threshold (J_AC^C) and the flow threshold (J_AC^F) increase with frequency. This is somewhat counterintuitive, as increasing the AC frequency often aids depinning by shaking the skyrmions, which can help them overcome the local pinning potential. This would typically lead one to expect a decrease in J_AC^C and J_AC^F with frequency. The authors should address and explain this trend more carefully. Is it possible that the increased thresholds are due to the finite inertia of the skyrmions or limitations in their response time? A more detailed explanation would greatly strengthen the discussion.

3. The authors argue that skyrmion deformation is reduced in the flow regime due to the averaging over pinning potentials. This is valid at moderate velocities, where the SkL moves smoothly and pinning is largely irrelevant. However, at higher skyrmion velocities, deformation can reappear due to spin-wave emission, as demonstrated in micromagnetic simulations (e.g., PRL 110, 207202 (2013)). Therefore, the statement that deformation is suppressed in the flow regime should be made more precise. The authors are encouraged to discuss under what conditions deformation might re-emerge, and whether such high-velocity effects could be observable in their frequency-current parameter space.

Minor Suggestions
The term “emergent reactance” is intriguing but non-standard. While it is defined via the imaginary part of the resistivity, it would be helpful to more explicitly contrast it with classical inductive or capacitive reactance for clarity.

Version 1:

Reviewer comments:

Reviewer #1

(Remarks to the Author)
The authors have addressed all my concerns. I believe this work would contribute to the understanding of skyrmion crystal dynamics from a different point of view. I would like to recommend the publication of this manuscript.

Reviewer #2

(Remarks to the Author)
The authors have addressed the comments and concerns I raised in my first review of the manuscript. Furthermore, it is my opinion that they have also answers criticisms raised by the other two referees.

I would also like to commend the authors on the thoroughness of their response and for approaching the reviews in a constructive manner (a nice change from the dismissive tone often used when responding to a peer review).

To summarize, I recommend that the paper is accepted for publication.

Reviewer #3

(Remarks to the Author)
The authors have provided detailed replies to all reviewer comments and made substantial revisions to the manuscript. While many of their responses are satisfactory, I find that their reply to Comment 1 remains inadequate and does not fully address the concern.

In the revised manuscript, the authors introduce a phenomenological fitting function to analyze their data and to define the depinning, creep, and flow regimes. However, the physical motivation for the specific choice of this fitting function remains unclear. The authors describe the function as an empirical form (an error function) that reproduces the observed current dependence but do not justify why this particular functional form should capture the underlying skyrmion dynamics.

Moreover, the definition of the thresholds for depinning, creep, and flow—such as the pinned-to-creep (P/C) and creep-to-flow (C/F) boundaries—depends sensitively on this arbitrary choice of function. Since these regimes are inherently crossovers rather than sharp transitions, the threshold values extracted in this way lack robustness and may vary with alternative fitting procedures. Therefore, the physical meaning of the defined boundaries is ambiguous.

It may not be possible to draw a clear distinction between the creep and flow regions, given their crossover nature. The authors should instead discuss how their results depend on the choice of the criteria used to separate these two regimes and evaluate the stability of their conclusions with respect to different definitions or fitting functions. Without such clarification, the claims regarding the precise identification of depinning, creep, and flow thresholds are not convincingly supported.

In summary, while the manuscript contains interesting experimental results, the central analysis and interpretation still rely on an empirically chosen fitting form without sufficient physical justification. As such, I cannot recommend publication of the current version. The authors are encouraged to reconsider the way they define and distinguish the different dynamical regimes and to test how their conclusions depend on these choices.

Version 2:

Reviewer comments:

Reviewer #3

(Remarks to the Author)
The authors have satisfactorily addressed all of my concerns regarding the phenomenological fitting used to define the pinned/creep/flow thresholds. Given these revisions and clarifications, I recommend acceptance of the current version for publication.

Open Access This Peer Review File is licensed under a Creative Commons Attribution 4.0 International License, which permits use, sharing, adaptation, distribution and reproduction in any medium or format, as long as you give appropriate credit to the original author(s) and the source, provide a link to the Creative Commons license, and indicate if changes were made.
In cases where reviewers are anonymous, credit should be given to 'Anonymous Referee' and the source.
The images or other third party material in this Peer Review File are included in the article's Creative Commons license, unless indicated otherwise in a credit line to the material. If material is not included in the article's Creative Commons license and your intended use is not permitted by statutory regulation or exceeds the permitted use, you will need to obtain permission directly from the copyright holder.
To view a copy of this license, visit https://creativecommons.org/licenses/by/4.0/

Reviewer #1:

In this manuscript, the authors unveil the emergent reactance induced by current-driven skyrmion lattice dynamics. By measuring the ac electrical resistivity in a typical chiral magnet MnSi device, the authors found the existence of emergent reactance within the skyrmion crystal phase. Furthermore, it is found that the reactance is most profound within the creep regime in the dynamical phase of the skyrmion crystal. In addition to the expected transverse emergent reactance, which can be attributed to the skyrmion crystal deformation and the corresponding emergent electric field, a longitudinal emergent reactance is also identified. The origin of this longitudinal emergent reactance could be due to the phason and spin-tilting modes of the skyrmion crystal (as it is a superposition of three spin helices) as the authors discussed.
This work provides solid evidence of the emergent reactance originating from the dynamics of a skyrmion crystal. The identification of emergent reactance would not only shed light on the understanding of skyrmion crystal dynamics but could also be harnessed for potential applications. I believe this manuscript will be of interest to the physics and engineering community. Before I can make a recommendation of the manuscript, I have a few concerns that I would like the authors to address.

We would like to sincerely thank reviewer 1 for the insightful comments and positive review of our work. We have addressed each comment below:

Comment 1:

I am confused with the sign of the imaginary resistivities [\rho_xy and \rho_xx]. Why is \rho_xy odd and \rho_xx even with respect to the magnetic field? From my understanding, they are all originate from the emergent electric field [Eq. (1)], i.e., the spin distribution and the spin dynamics. The emergent electric field preserves the time reversal symmetry. The authors also give an expression of the transverse resistivity Im\rho_xy in the discussion, which also preserves the time-reversal symmetry. Then the symmetry of \rho_xy and \rho_xx with respect to the magnetic field should be the same. How to understand these quantities from a symmetry point of view?

We thank the reviewer for this insightful comment. It is indeed correct that both $\mathrm{Im}[\rho_{yx}]$ and $\mathrm{Im}[\rho_{xx}]$ originate from the emergent electric field (EEF) [Eq. (1)]. To make this point clearer, we have modified the introduction (Page 2). It is also true that the EEF is even under the time-reversal operation. In the present case, $\mathrm{Im}[\rho_{yx}]$ and $\mathrm{Im}[\rho_{xx}]$ depend on the magnetic field as well as the skyrmion velocity or current. Because the time-reversal operation reverses not only the magnetic field but also the velocity and the current, the symmetries associated with time reversal and with magnetic-field reversal are not necessarily identical. This explains why the magnetic-field symmetries of $\mathrm{Im}[\rho_{yx}]$ and $\mathrm{Im}[\rho_{xx}]$ are different. To further clarify this point, it is useful to consider their physical origins in more detail:

As described in the manuscript, the transverse reactance $\mathrm{Im}[\rho_{yx}]$ arises from the EEF related to the translational motion of skyrmions, expressed as $\boldsymbol{e}_{\mathrm{em}} = -\boldsymbol{v}_{\mathrm{sk}} \times \boldsymbol{b}_{\mathrm{em}}$. Here, $\boldsymbol{v}_{\mathrm{sk}}$ and $\boldsymbol{b}_{\mathrm{em}}$ denote the skyrmion velocity and the emergent magnetic field, respectively. When the magnetic field is reversed, the emergent magnetic field also reverses, while the skyrmion velocity $\boldsymbol{v}_{\mathrm{sk}}$ does not because the direction of the current is fixed to define ρ_{yx}. Consequently, $\boldsymbol{e}_{\mathrm{em}}$ changes sign, and $\mathrm{Im}[\rho_{yx}]$ exhibits an antisymmetric response with respect to the magnetic field. Notably, because the time-reversal operation reverses both the skyrmion velocity and the magnetic field, the sign of $\boldsymbol{e}_{\mathrm{em}} = -\boldsymbol{v}_{\mathrm{sk}} \times \boldsymbol{b}_{\mathrm{em}}$ remains unchanged. This is consistent with the fact that the EEF is even under the time-reversal operation [Eq. (1)].

In the case of the longitudinal reactance, we conclude that $\mathrm{Im}[\rho_{xx}]$ is induced by the phason and spin-tilting modes of the skyrmion lattice as described in the main text. In a simple phenomenological calculation, the longitudinal reactance is given by $e_x = \frac{4\hbar Q}{4e}\partial_t\beta$, where Q and β are the wavevector of the skyrmion lattice and the spin-tilling mode, respectively [See Eq (9) in SI]. When the magnetic field is reversed, none of the components of e_x change sign, and thus, $\mathrm{Im}[\rho_{xx}]$ is symmetric with respect to the magnetic field. We note that the spin-tilting β is proportional to the current density and is odd under the time-reversal operation. In addition, ∂_t is also odd under the time-reversal operation. Consequently, $e_x = \frac{4\hbar Q}{4e}\partial_t\beta$ is even as a whole under the time-reversal operation, again consistent with the EEF (Eq. 1).

In the revised manuscript, we have added the discussion about the symmetry of reactance signals in Methods on Pages 14-15, as follows:

> *"**Transport Measurements***
> *All transport measurements were performed within a Quantum Design Physical Properties Measurement System (PPMS). The MnSi devices were mounted on a standard PPMS puck using GE varnish. Cu wires were used to contact the substrate with a combination of solder and colloidal silver paste. Measurements of the longitudinal and Hall resistance and reactance were taken simultaneously using the lock-in technique (Stanford Instruments SR830). For the longitudinal resistivity $Re[\rho_{xx}]$ and the transverse resistivity $Re[\rho_{yx}]$, the data were symmetrized and anti-symmetrized in the conventional manner i.e. $Re[\rho_{xx}] = \{Re[\rho_{xx}^{measured}(+B)] + Re[\rho_{xx}^{measured}(-B)]\}/2$ and $Re[\rho_{yx}] = \{Re[\rho_{yx}^{measured}(+B)] - Re[\rho_{yx}^{measured}(-B)]\}/2$. For the longitudinal reactance $Im[\rho_{xx}]$ and transverse reactance $Im[\rho_{yx}]$, the recorded signals were first corrected for extrinsic contributions (see Supplementary Note 1 for details). The corrected data were then symmetrized and anti-symmetrized with respect to the magnetic field. Since the longitudinal reactance is symmetric with respect to the magnetic field [21], it was symmetrized with respect to the magnetic field. In contrast, the transverse reactance originates from the emergent electric field due to the translational motion of skyrmions, given by $\boldsymbol{e}_{em} = -\boldsymbol{v}_{sk} \times \boldsymbol{b}_{em}$. In this case, when the magnetic field is reversed, the emergent magnetic field $\boldsymbol{b}_{em}$ is reversed, leading to the sign change of $\boldsymbol{e}_{em}$. Consequently, the transverse reactance is anti-symmetric with respect to the magnetic field, and thus it was anti-symmetrized.*
>
> *For the AC+DC measurements, a small AC oscillation was superimposed on a large DC bias using a Keithley 6221 current source. The AC peak amplitude was set to $1.74 \times 10^8 Am^{-2}$ at a frequency of 509 Hz, and the output TTL pulse was used as the lock-in reference signal. In this case we technically measure the gradient of the I-V curve according to dV/dI, which we convert into the differential resistivity following $\rho^{diff} = t\, dV/dI$, where t denotes the sample thickness."*

Comment 2:

In the pinned regime, the skyrmion would also deform [e.g., as shown in PNAS 119, e2200958119 (2022)]. Why cannot such deformation induce an emergent reactance? Related to this point, how do the authors rule out the possibility of a longitudinal emergent electric field generated by a deforming skyrmion crystal?

We thank the reviewer for these two important points. We will answer them separately as follows:

Firstly, it is indeed possible for the deformation of pinned skyrmions to induce an emergent reactance, as proposed theoretically by Furuta *et. al.* npj spintronics **1**, 3 (2023) in which the reactance is predicted to exhibit a positive sign. In fact, we observe the longitudinal emergent reactance arising from pinned skyrmions in the temperature range 25 – 28 K in addition to that arising from moving skyrmions observed in the range 22 – 25 K As shown in Fig. 1g and Figs. S7g and I, the positive reactance is clearly visible between 25 – 28 K. In MnSi, with increasing temperature, the threshold current densities increases as reported in [T. Shulz et al., Nat. Phys. **8**, 301 (2012).]. Schematically, these changes to the skyrmion velocity curve are represented in Fig. R1:

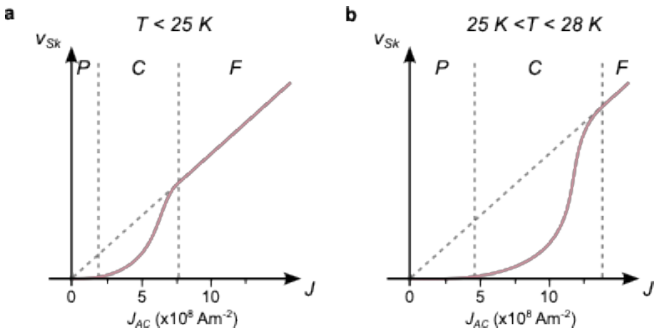

Figure R1: Schematic skyrmion velocity as a function of current density below at different temperatures

Therefore, in this temperature range (25-28 K), the skyrmion lattice remains pinned. This interpretation is consistent with measurements of the topological Hall effect which exhibits no change with increasing current density throughout this region (Fig. S7e). Consequently, the positive reactance observed between 25-28 K may arise from the deformation of a pinned skyrmion lattice. Furthermore, this positive reactance decreases with increasing current density (see Fig. 1g and Figs. S7g and i), since the skyrmions begin to depin at higher current densities, thereby reducing the positive reactance arising from the deformation of pinned skyrmions.

In response to the second question: *"how do the authors rule out the possibility of a longitudinal emergent electric field generated by a deforming skyrmion crystal?"*, First, since whether skyrmions are pinned or moving can be determined from THE, the emergent reactance signals resulting from pinned skyrmions and from moving skyrmions are distinguishable. In addition, in the present case, the signs of the emergent reactance arising from these two mechanisms are opposite, also allowing us to distinguish them. Incidentally, the excitation of phason and spin-tilting modes can be regarded as a deformation of the skyrmion lattice [G. Tatara et al, JPSJ **83**, 104711 (2014).]. In other words, we can also state that the deformation of a moving skyrmion lattice generates a negative emergent reactance, whereas the deformation of a pinned skyrmion lattice produces a positive emergent reactance.

While we have already presented the above discussion in the penultimate paragraph of the main text and in Supplementary Note 7, we have revised the description to add more detail and improve readability, as follows:

> ***"Supplementary Note 7***
> *In Fig. S7a-c we present the colour map of THE in the magnetic phase diagrams for three different current densities ($J_{AC} = 0.68 \times 10^8 Am^{-2}, 4.26 \times 10^8 Am^{-2}, and\ 6.81 \times 10^8 Am^{-2}$). The THE is calculated using the linear fit procedure described in Supplementary Note 2 (hence the discrepancies in the helical and conical phases), and phase boundaries are overlaid according to those calculated for Hall resistivity measured with $J_{AC} = 4.26 \times 10^8 Am^{-2}$ using the method outlined in Methods. The THE exhibits distinct behaviour across three temperature ranges. As already shown in Fig. 2 of the main text, in the centre of the skyrmion phase (22-25 K), the THE decreases with increasing current density. This trend is also confirmed by the isothermal cuts at 23 K measured at each current density (Fig. S7d). The reduction of THE results from the current-induced motion of the SkL. In contrast, close to the paramagnetic transition temperature (25-28 K), THE does not depend on the current density as shown in Figs. S7a-c, and e. This behaviour suggests that the SkL remains pinned. Finally, at low temperatures (below 22 K), the THE measured at low current density is smaller than that observed at the center of the SkL phase, indicating that the skyrmion density is reduced due to its coexistence with the conical phase. Furthermore, with increasing current density, the THE increases, which can be ascribed to small Joule heating effects, and/or current induced skyrmion nucleation [10].*
> *These results have some important implications for the interpretation of the temperature dependence of the emergent reactance. In correspondence with the THE, the emergent reactance also exhibits distinct behaviour across the three temperature ranges. In the centre of the skyrmion phase (22-25 K), both $Im[\rho_{yx}]$ and $Im[\rho_{xx}]$ show prominent signals. This temperature range corresponds to the range in which the SkL can move, as discussed above, supporting our main conclusion that the reactance originates from the SkL motion. In contrast, close to the paramagnetic transition temperature (25-28 K), a different behaviour is observed. First, $Im[\rho_{yx}]$ falls below the noise level. As discussed above, since the SkL remains pinned in this temperature range, it is reasonable that the electric field arising from translational motion vanishes. Interestingly, in this temperature range, we find that $Im[\rho_{xx}]$ is not zero but takes a small positive value. This positive $Im[\rho_{xx}]$ may be interpreted as an emergent reactance arising from the current-induced deformation of pinned skyrmions, which is predicted to exhibit a positive emergent reactance [11]. Furthermore, this positive reactance decreases with increasing current density (Supplementary Figs. S7g and i), suggesting the skyrmions begin to be depinned at higher current densities, thereby reducing the positive reactance arising from the deformation of pinned skyrmions. Finally, at low temperatures (below 22 K) both $Im[\rho_{yx}]$ and $Im[\rho_{xx}]$ drop below the noise level. The absence of the reactance signals at low temperatures is a consequence of the skyrmion metastability. Since metastable skyrmions are easily destroyed or created by current application as discussed above, the dynamical phase diagram differs from that in the centre of the skyrmion phase, in which emergent reactance is observed. In addition, the phason and spin-tilting modes are not well-defined for isolated*

metastable skyrmions. Therefore, only the equilibrium skyrmions lattice exhibits the expected dynamics discussed in the main text, and the emergent reactance."

Comment 3:

What determines the amplitude of the emergent reactance? I guess it may be related to the strength of the emergent magnetic field. Also do the authors expect that such emergent reactance can be further enhanced in spin textures beyond two dimensions like skyrmion bundles [e.g., Nat. Nanotechn. 16, 1086 (2021)], hedgehogs [Nat. Commun. 10, 1059 (2019)], and hopfions [e.g., Phys. Rev. Lett. 129, 267201 (2022)]? A related discussion could be given for future research directions.

Since the equation of the EEF involves the spatial derivative of the spin direction [see Eq (1)], a short-pitch spin texture gives rise to a large EEF, and consequently a large emergent reactance. Accordingly, in the case of skyrmions or the skyrmion lattice, the magnitude of the emergent reactance increases with decreasing skyrmion size (aside from a constant factor). For example, in our phenomenological calculation [Eq. (S9) in the Supplementary Information], the longitudinal emergent reactance is proportional to the wavenumber of the skyrmion lattice (Q), indicating that smaller skyrmions yield larger longitudinal emergent reactance. In addition, the transverse emergent reactance depends on the emergent magnetic field, which becomes large for small skyrmions. This then suggests that the emergent reactance can be enhanced in nanoskyrmion materials such as Gd_2PdSi_3 and $Gd_3Ru_4Al_{12}$. We have added this description to Supplementary Note 6, as follows:

> *"For both traverse and longitudinal emergent reactance, a large signal is expected for a short period skyrmion lattice. The transverse emergent reactance depends on the emergent magnetic field, which becomes large for small skyrmions. In addition, according to Eq. (S9), the longitudinal emergent reactance is proportional to the wavevector of the skyrmion lattice (Q), indicating that smaller skyrmions yield larger longitudinal emergent reactance."*

The longitudinal emergent reactance is related to the emergent electric field induced by a time-varying noncollinear spin texture, and the transverse emergent reactance is related to the dynamics of topological spin textures. Thus, other topological spin textures such as skyrmion bundles, hedgehogs, and hopfions can also induce emergent reactance. In addition, similar to the skyrmion case discussed above, the magnitude of the emergent reactance in these spin textures depends on their size, and thus we anticipate that nanometric skyrmion bundles and hedgehogs can induce a large emergent reactance. In the revised manuscript, to highlight that these other spin textures may also generate the emergent reactance and to stimulate possible future research directions, we have included a short discussion on page 13, as follows:

> *"Notably, the low critical current densities required for SkL creep motion may provide an advantage over helical and domain wall systems in generating emergent reactance for spintronic applications [22, 24]. Furthermore, nanoscale topological spin textures other than conventional skyrmions, such as skyrmion bundles [47], hedgehogs [48], and hopfions [49] can potentially generate emergent reactance through the same mechanisms."*

Reviewer #2:

The manuscript reports on measurements of the complex reactance in a MnSi device, in order to determine contributions from the skyrmion phase. This is a valid goal as most information about skyrmion dynamics comes from direct imaging techniques such as LTEM. Developing complementary techniques based on transport measurements is therefore very attractive to advancing the field.
Over all the manuscript is quite well written, and the measurements themselves appear to be sound.

We sincerely thank reviewer 2 for their in-depth and critical review of our manuscript. We believe that, in addressing each of the concerns, our manuscript and interpretation of the data is now more robust. Before addressing the specific comments, we first wish to address some of the more general comments related to the data processing, error analysis, and justification of the skyrmion contribution.

General comment 1:

My concern is the substantial amount of data processing necessary to bring out the features attributed to the skyrmion creep. This requires a very high degree of confidence in all the steps of the analysis, and in this regard, I am afraid the manuscript falls short (for example there is not a single error bar shown on any of the measurements or derived quantities!)
I am also not sure that attributing the results to the skyrmions is as definitive as the authors suggest.

Firstly, we would like to note that error bars were present in the original manuscript, with sizes often smaller than the data point size and were therefore not visible in some cases. To make this clearer, we have indicated this in the corresponding figure captions.

In addition, Reviewer 2 raises some concerns regarding the data analysis. First, we would like to emphasize that the signals observed in the skyrmion lattice phase is clearly visible even in the raw data (Fig. S1). Therefore, the observed signals are not artifacts arising from the data analysis procedure. We would also like to note that, in complex impedance measurements, corrections for contributions from signals other than the intrinsic one are commonly performed. Importantly, the correction procedure must be chosen in a reasonable manner depending on the phenomena under investigation. In the present study, we employed the following correction procedure:

The measured imaginary part can arise from both intrinsic effects from the sample $X_{\mathrm{Intrinsic}}$ and extrinsic effects $X_{\mathrm{Extrinsic}}$ such as parasitic capacitance. In addition, extrinsic effects such as parasitic capacitance and imperfections of the reference signal cause an additional phase rotation ϕ, termed *"trivial rotation"* in this manuscript, which mixes the real and imaginary components. Overall, the measured complex impedance can be expressed as

$$Z = e^{-i\phi}(R + X_{\mathrm{Intrinsic}} + X_{\mathrm{Extrinsic}}),$$

where *R* is the sample resistance.

First, we correct for the trivial rotation. The effect of the trivial rotation can clearly be seen in an updated version of Fig. S1, in which we now display the real parts of the longitudinal and Hall resistivities (Figs. S1a and b) and the raw data of the imaginary parts of the longitudinal and transverse reactance (Figs. S1c and d). It can be clearly seen that, without correction, the imaginary component takes on a similar form to the real part except for the prominent emergent reactance signals in the skyrmion phase. This similarity results from the mixing of resistance and reactance by the trivial phase rotation. To correct trivial rotation, since the sample is not expected to exhibit any reactance in the field polarised (FP) region, we determine the trivial rotation from the FP region, apply the same rotation correction to the entire magnetic field range, and find that prominent signals still remain within the skyrmion phase (Figs. S1e and f).

Next, after correcting the trivial rotation, we separate the intrinsic contribution from the extrinsic reactance arising from unavoidable parasitic capacitance or inductance from un-shielded cables, poor ground connections, or unoptimized components. The extrinsic reactance is symmetric against the magnetic field, and so for the Hall measurement, it is removed during the antisymmetrisation procedure. However, for the longitudinal component, the extrinsic component is not removed, as the signal itself is symmetric, and so we must perform an additional extrinsic subtraction procedure to obtain the intrinsic reactance. Since the extrinsic reactance is independent of

the magnetic field, we perform this subtraction by fitting a straight line to the field polarised region and then subtracting this from the complete magnetic field sweep.

Our correction procedure is further validated by the following points. First, although the determination of the trivial rotation and extrinsic reactance was performed in the field polarized (FP) region, the corrected reactance is also zero in the conical and helical phases, in which the reactance is likewise expected to be zero. Second, when we perform the same correction procedure at 15 K, in which no skyrmion contribution is expected, the correction yields no imaginary contributions (Figs. S1i-p), indicating that the finite signal in the skyrmion phase does not result from our correction procedure. Accordingly, we have adapted Supplementary Note 1 and Figure S1 to explain the correction procedure and its validity in more depth as follows:

> "*The impedance measurements include both the resistance (R) and intrinsic inductance/capacitance* ($X_{Intrinsic}$) *of our sample, as well as an extrinsic inductance/capacitance* ($X_{Extrinsic}$) *arising from parasitic effects from the cables, connectors, and instruments, causing a systematic but frequency dependent rotation of the signal phase [1]. In addition, extrinsic effects such as parasitic inductance/capacitance and imperfections of the reference signal result in mixing of the true real an imaginary components of the complex impedance. Overall, the measured complex impedance can be expressed as*
>
> $$Z = e - i\phi \left[R + X_{Intrinsic} + X_{Extrinsic}\right],$$
>
> *where R and* ϕ *are the sample resistance and the phase rotation, termed "trivial rotation", respectively. We correct these extrinsic effects as follows:*
>
> *First, we correct for the trivial rotation. The effect of the trivial rotation can clearly be seen in Figs. S1a-d, which display the real parts of the longitudinal and Hall resistivities (Figs. S1a and b) and the raw data of the imaginary parts of the longitudinal and transverse reactance (Figs. S1c and d). It is evident that, without correction, the imaginary component takes on a similar form to the real part except for the prominent emergent reactance signals in the skyrmion phase. This similarity results from the mixing of resistance and reactance due to the trivial phase rotation. To correct for the trivial rotation, since the sample is not expected to exhibit any reactance in the field polarised (FP) region, we determine the trivial rotation from the FP region and apply the same rotation correction to the entire magnetic field range. As shown in Figs. S1e and f, after correcting for the trivial rotation, prominent signals still remain in the skyrmion phase.*
>
> *Next, after correcting the trivial rotation, we separate the intrinsic contribution from the extrinsic reactance* ($X_{Extrinsic}$)*. The extrinsic reactance is symmetric against the magnetic field, and so for the Hall measurement, it is removed during the antisymmetrisation procedure. However, for the longitudinal component, the extrinsic contribution is not removed, since the signal itself is symmetric, and thus we must perform an additional extrinsic subtraction procedure to obtain the true magnitude of the signal arising from the skyrmions. Because the extrinsic reactance is independent of the magnetic field, we perform this subtraction by fitting a straight line to the field polarised region and then subtracting this from the complete magnetic field sweep. The final corrected reactance signals are shown in Figs. S1g and h.*
>
> *Our correction procedure is further validated by the following points. First, although the determination of the trivial rotation and extrinsic reactance was performed in the field polarized (FP) region, the corrected reactance is also zero in the conical and helical phases, in which the reactance is likewise expected to be zero. Second, when we perform the same correction procedure at 15 K, in which no skyrmion contribution is expected, the correction yields no imaginary contributions (Figs. S1i-p), indicating that the finite signal in the skyrmion phase does not result from our correction procedure."*

We believe that this revision now more clearly supports our conclusion that the measured reactance indeed originates from the skyrmion phase.

General comment 2:

I am also not sure that attributing the results to the skyrmions is as definitive as the authors suggest. In particular I am puzzled why the signal is mostly seen in a very narrow range of temperatures of 24-25 K? While the absence of a signal at higher temperature may be due an increase in. the critical current as stated by the authors, why is it also absent a lower temperature?

We thank the reviewer for their comment and are happy to note that this behaviour can be explained by both the temperature dependence of the creep and flow threshold current densities, and temperature dependent stability of the skyrmion lattice.

In the higher temperature regime, Reviewer 2 is correct: the softening of the magnetisation from larger thermal fluctuations effectively increases the pinning efficiency, thereby increasing the critical current required for creep motion. Therefore, in the phase diagram in Figs. 1 c, e, and g, a constant driving current probes different regions of the skyrmion velocity curve, and at higher temperatures, the applied current is insufficient to reach creep motion. This explains the small signals observed in $\mathrm{Im}[\rho_{yx}]$ and $\mathrm{Im}[\rho_{xx}]$ between 25 – 28 K.

The absence of signal in the lower-temperature regime, $T < 22$ K, arises from the metastability of the skyrmion lattice. For thin devices, the skyrmion region is often expanded to lower temperatures and higher magnetic fields. In such cases, since the energy difference between the two competing states (conical structures and skyrmions) is very small, skyrmions can form as a metastable state. In the present case, likewise, the skyrmion state is metastable and coexists with the conical structure below 22 K. This is supported by the smaller topological Hall signal in this regime compared with that at the centre of the skyrmion phase (Fig. 1c in the main text), because the smaller signal suggests a smaller volume fraction of skyrmions. Such metastable skyrmions cannot generate the emergent reactance discussed in this study for the following reason. The energy barrier between skyrmions and conical structures can be easily overcome by a small perturbation from an electric current. In fact, we observe an enhancement of the topological Hall signal with increasing current density in the lower-temperature regime of the skyrmion phase, which indicates current-induced creation of skyrmions (see Supplementary Note7 and Fig. S7). In other words, when an AC current is applied, metastable skyrmions can be easily created and destroyed, and thus the dynamical phase diagram differs from that in the centre of the skyrmion phase, in which emergent reactance is observed (23 - 25 K).

While the above discussions are already presented in the penultimate paragraph of the main text on page 12 and in Supplementary Note 7, we have revised them to provide more detail and improve readability, as follows;

> ***"Supplementary Note 7***
> *In Fig. S7a-c we present the colour map of THE in the magnetic phase diagrams for three different current densities ($J_{AC} = 0.68 \times 10^8 Am^{-2}, 4.26 \times 10^8 Am^{-2}, and\ 6.81 \times 10^8 Am^{-2}$). The THE is calculated using the linear fit procedure described in Supplementary Note 2 (hence the discrepancies in the helical and conical phases), and phase boundaries are overlaid according to those calculated for Hall resistivity measured with $J_{AC} = 4.26 \times 10^8 Am^{-2}$ using the method outlined in Methods. The THE exhibits distinct behaviour across three temperature ranges. As already shown in Fig. 2 of the main text, in the centre of the skyrmion phase (22-25 K), the THE decreases with increasing current density. This trend is also confirmed by the isothermal cuts at 23 K measured at each current density (Fig. S7d). The reduction of THE results from the current-induced motion of the SkL. In contrast, close to the paramagnetic transition temperature (25-28 K), THE does not depend on the current density as shown in Figs. S7a-c, and e. This behaviour suggests that the SkL remains pinned. Finally, at low temperatures (below 22 K), the THE measured at low current density is smaller than that observed at the center of the SkL phase, indicating that the skyrmion density is reduced due to its coexistence with the conical phase. Furthermore, with increasing current density, the THE increases, which can be ascribed to small Joule heating effects, and/or current induced skyrmion nucleation [10].*
> *These results have some important implications for the interpretation of the temperature dependence of the emergent reactance. In correspondence with the THE, the emergent reactance also exhibits distinct behaviour across the three temperature ranges. In the centre of the skyrmion phase (22-25 K), both $Im[\rho_{yx}]$ and $Im[\rho_{xx}]$ show prominent signals. This temperature range corresponds to the range in which the SkL can move, as discussed above, supporting our main conclusion that the reactance originates from the SkL motion. In contrast, close to the paramagnetic transition temperature (25-28*

K), a different behaviour is observed. First, $Im[\rho_{yx}]$ falls below the noise level. As discussed above, since the SkL remains pinned in this temperature range, it is reasonable that the electric field arising from translational motion vanishes. Interestingly, in this temperature range, we find that $Im[\rho_{xx}]$ is not zero but takes a small positive value. This positive $Im[\rho_{xx}]$ may be interpreted as an emergent reactance arising from the current-induced deformation of pinned skyrmions, which is predicted to exhibit a positive emergent reactance [11]. Furthermore, this positive reactance decreases with increasing current density (Supplementary Figs. S7g and i), suggesting the skyrmions begin to be depinned at higher current densities, thereby reducing the positive reactance arising from the deformation of pinned skyrmions. Finally, at low temperatures (below 22 K) both $Im[\rho_{yx}]$ and $Im[\rho_{xx}]$ drop below the noise level. The absence of the reactance signals at low temperatures is a consequence of the skyrmion metastability. Since metastable skyrmions are easily destroyed or created by current application as discussed above, the dynamical phase diagram differs from that in the centre of the skyrmion phase, in which emergent reactance is observed. In addition, the phason and spin-tilting modes are not well-defined for isolated metastable skyrmions. Therefore, only the equilibrium skyrmions lattice exhibits the expected dynamics discussed in the main text, and the emergent reactance."

We hope that, with these amendments to the text and supplementary, Reviewer 2 is more convinced of our interpretation of the data, principally that the reactance originates from the skyrmion phase.

We will now respond to specific comments as follows:

Specific comment 1:

The claim that the observed reactance could be useful for applications seems far-fetched given how subtle the effect, requiring substantial subtractions of other contributions to the measured signal.

With the above discussion, we hope that the concerns over the subtractions are addressed, and it is clearer now that the extrinsic contributions would not be significant when implemented on a device level. For device-level implementation of the emergent reactance, sources of extrinsic reactance would be minimised such that only the emergent reactance contributes to a change in the phase of the response. Additionally, we anticipate that the strength of the signal may be enhanced in other skyrmion systems optimised for signal magnitude i.e. those with smaller sized skyrmions or topological spin textures. However, our choice of material in this case was dictated by a system with well-studied skyrmion dynamics such that we may demonstrate the emergent reactance as a proof-of-concept. For future applications, further characterisation and optimisation of the signal magnitude and frequency response may be necessary.
In the revised manuscript, we have added a description of future perspectives to exploring systems with much larger emergent reactance to Supplementary Note 6, as follows:

"For both traverse and longitudinal emergent reactance, a large signal is expected for a short period skyrmion lattice. The transverse emergent reactance depends on the emergent magnetic field, which becomes large for small skyrmions. In addition, according to Eq. (S9), the longitudinal emergent reactance is proportional to the wavevector of the skyrmion lattice (Q), indicating that smaller skyrmions yield larger longitudinal emergent reactance."

Specific comment 2:

In discussing Fig. 2c it is stated that the skyrmion velocity shows a difference between the creep and flow states. This is not merited. Only a single data point was measured in the flow state. Also, it is not accurate to say the velocity is close to that of the electrons when the dashed lines show 0.75 v_e.

Firstly, we would like to apologize that a data point was plotted outside the range of the plot in Fig. 2b. We have corrected this error. However, the data points in the flow region remain limited. To confirm the validity of our estimation, we estimated the skyrmion lattice velocity for six magnetic field values (0.21 T– 0.26 T), in which the flow regime has at least two data points, using the same analysis procedure (Fig. S5). For all magnetic fields

examined, we find that the skyrmion lattice velocity in the flow regime takes similar values in the range from $0.75v_e$ to $0.80v_e$, with an average of $0.78(3)v_e$. Therefore, we can conclude that the estimation of the skyrmion lattice velocity is reliable, although the number of data points in the flow region is limited. Incidentally, we find that outside this magnetic field range (*B* > 0.26 T), because of the increase in the flow thresholds, we do not obtain a sufficient number of points in the flow region within the present measurement range of the current density. Therefore, we did not include them in this discussion.

With these calculations in mind, we agree with Reviewer 2 that the skyrmion velocity is not close to that of the electron velocity and have since amended the text to state that v_{sk} 'approaches' v_e (Page 6). However, we would like to point out that the difference between the electron velocity and the skyrmion lattice velocity is not very large due to the multi-band nature of MnSi [O. Nakanishi et al., J. Mag. Mag. Mat. **15**, 879 (1980)]. While we calculate the electron velocity using the single-carrier Drude model, in general, the corresponding electron velocity is not precise in a multi-band system and only provides an estimate of the order of magnitude [M. T. Birch et al, Nature **633**, 554 (2024)]. Thus, we anticipate that the differences occur from the approximations used in the single-carrier Drude model, and since v_{sk} is a well-defined quantity, the similarity in their orders of magnitude provides good agreement considering the multi-band nature of MnSi.

We have adapted Fig. S3 to highlight the consistency of the skyrmion lattice velocity in the flow regime and provided a detailed account of the procedures used to estimate the skyrmion lattice velocity with respect to the electron velocity in Supplementary Note 3, as follows:

> ***"Velocity of skyrmion lattice and electron***
> *To investigate the skyrmion velocity in the flow regime, we have evaluated the skyrmion velocity at various magnetic fields. The ratio of the skyrmion velocity to the electron velocity (v_e) is shown in the upper panels of Figs. S3a-g. We find that the skyrmion velocity takes similar values in the range from $0.75v_e$ to $0.80v_e$, with an average of $0.78(3)v_e$, indicating that the fitting used to estimate of the skyrmion lattice velocity is reliable. We note that under Galilean relativity, the skyrmion velocity is expected to approach the conduction electron velocity, namely $v_{sk} = v_e$, ideally. However, because MnSi is a multi-band system, the electron velocity calculated using the Drude model only provides an estimation of the order of magnitude [3, 6]."*

Specific comment 3:

The discussion of Fig. 4, why the imaginary part of rho_xy changes sign and why the maxima cross the phase boundaries is handwaving and thus not very compelling.

Following the reviewer's comment, we have discussed the sign of $\mathrm{Im}[\rho_{yx}]$ and the maximum position of $\mathrm{Im}[\rho_{xx}]$ in more details as follows:

-Sign change

Firstly, as discussed in the main text and Supplementary Note 6, we attribute the $\mathrm{Im}[\rho_{yx}]$ signal to the inertial dynamics of skyrmions, which can be described by the Thiele equation: $m_{\mathrm{sk}}\dot{\boldsymbol{v}}_{\mathrm{sk}} + \boldsymbol{G} \times (\boldsymbol{v}_{\mathrm{e}} - \boldsymbol{v}_{\mathrm{sk}}) + D(\beta\boldsymbol{v}_{\mathrm{e}} - \alpha\boldsymbol{v}_{\mathrm{sk}}) + \boldsymbol{\nabla}V_{\mathrm{pin}} = 0$, where $\boldsymbol{G}$, D, β, α, V_{pin}, and m_{sk} are the gyro-coupling vector, dissipative force tensor, dimensionless constant characterizing the nonadiabatic electron spin dynamics, Gilbert damping constant, pinning potential, and the skyrmion mass, respectively. The imaginary part of the skyrmion velocity $\mathrm{Im}[v_{\mathrm{sk}}]$ is related to $\mathrm{Im}[\rho_{yx}]$as follows: $\mathrm{Im}[\rho_{yx}] = Pb_{em}\,\mathrm{Im}[v_{\mathrm{sk}}]/j_{\mathrm{AC}}$. In this Thiele equation, because V_{pin} is a nonlinear function of the skyrmion position, it is difficult to obtain a general solution for the skyrmion velocity. Nevertheless, to gain physical insight, we discuss the case of $V_{\mathrm{pin}} \cong 0$. Physically, this assumption means that the influence of the pinning potential becomes weak, although the skyrmion mass m_{sk} remains finite. This provides a good approximation of the dynamics in the creep region close to the flow threshold current. In this case, the Thiele equation can be solved, and the skyrmion velocity along the current direction (*x* direction) is given by

$$v_{sk}^{x} = \frac{\alpha\beta D^2 + G^2 - i\omega m_{\mathrm{sk}} D\beta}{G^2 + (\alpha D)^2 - m_{\mathrm{sk}}^2\omega^2 - 2i\alpha D m_{\mathrm{sk}}\omega} v_e.$$

This result explains the sign change as a function of the frequency: In the high frequency limit, the imaginary part of v_{sk}^{x} is given by $\mathrm{Im}[v_{sk}^{x}] = \frac{D\beta}{\omega m_{\mathrm{sk}}} v_{\mathrm{e}}$ and is positive. In the low frequency limit $\omega \sim 0$, the imaginary part of v_{sk}^{x} is given by

$$\mathrm{Im}[v_{sk}^{x}] = \frac{D m_{\mathrm{sk}}}{G^2 + (\alpha D)^2} \left[\frac{2\alpha(D^2\alpha\beta + G^2)}{G^2 + (\alpha D)^2} - \beta \right] \omega v_e.$$

For example, for typical parameters in metals [$\alpha = 0.02$ $\beta = 0.05$, $D = 5.57\pi$, $G = 4\pi$, J. Iwasaki, et al, Nat. Commun. 4, 1463 (2013).], $\mathrm{Im}[v_{sk}^{x}]$ is negative. In other words, in this case, the sign of $\mathrm{Im}\,[v_{\mathrm{sk}}^{x}]$ changes from negative to positive with increasing frequency, consistent with experimentally observed sign change. We note that, for another parameter set, [$\alpha = 0.04$ $\beta = 0$, $D = 5.57\pi$, $G = 4\pi$, J. Iwasaki, et al, Nat. Commun. 4, 1463 (2013).], $\mathrm{Im}\,[v_{sk}^{x}]$ is positive at small ω. Thus, depending on the damping parameters, the low-frequency limit of $\mathrm{Im}\,[v_{\mathrm{sk}}^{x}]$ can take either positive or negative values. In the revised manuscript, we have added this discussion to Supplementary Note 6, as follows:

"…In contrast, in the flow region, since $m_{Sk} = 0$ and $\nabla V_{pin} \sim 0$ due to the absence of the internal deformation and a relative reduction of pinning force, the skyrmion velocity is equal to the electron velocity. Therefore, the emergent electric field induced by the translational motion of SkL is given by $e_{em} = -v_{Sk} \times b_{em} = -v_e \times b_{em} \propto j_{AC} \sin(\omega t) \times b_{em}$, and the transverse reactance component disappears.

Next, we discuss the sign of the transverse reactance. In this Thiele equation, because V_{pin} is a nonlinear function, it is difficult to obtain a general solution for the skyrmion velocity. Nevertheless, to gain physical insight, we consider the case of $V_{pin} \simeq 0$. Physically, this assumption means that the influence of the pinning potential becomes weak, although m_{sk} remains finite. This provides a good approximation of the dynamics in the creep region close to the flow threshold current. In this case, the Thiele equation can be solved, and the skyrmion velocity along the current direction (x direction) is given by

$$v_{sk}^{x} = \frac{\alpha\beta D^2 + G^2 - i\omega m_{\mathrm{sk}} D\beta}{G^2 + (\alpha D)^2 - m_{\mathrm{sk}}^2\omega^2 - 2i\alpha D m_{\mathrm{sk}}\omega} v_e.$$

This result explains the experimentally observed sign change of $Im[\rho_{yx}]$: In the high frequency limit, the imaginary part of v_{sk}^{x} is given by $Im[v_{sk}^{x}] = \frac{D\beta}{\omega m_{sk}} v_e$ and is positive. In the low frequency limit $\omega \simeq 0$, the imaginary part of v_{sk}^{x} is given by

$$\mathrm{Im}[v_{sk}^{x}] = \frac{D m_{\mathrm{sk}}}{G^2 + (\alpha D)^2} \left[\frac{2\alpha(D^2\alpha\beta + G^2)}{G^2 + (\alpha D)^2} - \beta \right] \omega v_e.$$

For example, for typical parameters in metals [$\alpha = 0.02, \beta = 0.05, D = 5.57\pi, G = 4\pi$ [4].], $Im[v_{sk}^{x}]$ is negative. In other words, in this case, the sign of $Im[v_{sk}^{x}]$ changes from negative to positive with increasing frequency, consistent with the experimentally observed sign change of $Im[\rho_{yx}]$. We note that, for another parameter set, $\alpha = 0.04, \beta = 0, D = 5.57\pi, G = 4\pi$, $Im[v_{sk}^{x}]$ is positive. Thus, depending on the parameters, the low-frequency limit of $Im[v_{sk}^{x}]$ can take either positive or negative values."

-Maximum value

In this paper, we employee two methods to measure the reactance: AC+DC measurement (Fig. 2) and AC measurement (Figs. 1, 3, and 4). In the AC+DC measurements, we apply a large DC current to drive skyrmions and superpose a small AC current for lock-in detection. In this case, only a single dynamical phase of the SkL (i.e., pin/creep/flow), determined by the magnitude of the DC current, contributes to the voltage response. In contrast, in the AC measurements, we apply a large AC current, which both drives skyrmions and is used for lock-in detection. In this case, over a full current cycle, the skyrmion passes through multiple dynamical phases, all of which contribute to the measured voltage response, as discussed in Figs. 3a and b. As a consequence, the peaks are smeared and their positions shift to the high current density region.

To discuss this point in more detail, we calculate the current dependence of the reactance response measured with AC+DC current and with AC current by adopting a toy model. In this calculation, we assume that the voltage is proportional to the time derivative of the current and that its coefficient depends on the current. Specifically, we assume the following equation: $V = (e^{-(J+4)^2} + e^{-(J-4)^2})dJ/dt$, in which the coefficient of dJ/dt peaks at J = ±4 and is nearly zero in both the low- and high-current regions (Fig. R2a). The current region where the coefficient is nonzero corresponds to the creep region in the experiment. Then, we calculate the time dependence of the voltage response when applying AC+DC current $J = J_{\mathrm{DC}} + J_{\mathrm{AC}}\sin\omega t$ and AC current $J = J_{\mathrm{AC}}\sin\omega t$ (Gray-dotted curves in Figs R1b and c). For the AC+DC current, the voltage response is nonzero only when J_{DC} lies within the current range where the coefficient is nonzero (Fig. R2b). In contrast, for the AC current, even when the amplitude of the AC current exceeds the current range where the coefficient is nonzero, a finite voltage signal appears because the current passes through the low current region with a nonzero coefficient (Fig. R2c).

From these time dependences of the voltage, we calculate the current dependence of the real and imaginary parts of the first-harmonic voltage, which corresponds to what we measured in our experiment. As shown in Fig. R2d, for the AC+DC current, the current dependence of the imaginary component exhibits a peak structure around J = 4, similar to the current dependence of the coefficient of dJ/dt. In contrast, for the AC current, the peak is smeared, and their positions shift to the high current region. Again, this occurs because the current transits the nonzero-coefficient region even at higher current amplitudes. The experimentally observed shift of the maximum position of $\mathrm{Im}[\rho_{xx}]$ is also caused by the same mechanism. In addition, in our experiment, the longitudinal reactance measured with AD+DC current peaks in the creep region (Fig. S4), which is also supports this mechanism. In the revised manuscript, we have added this discussion to Supplementary Note 5, as follows:

*"**SUPPLEMENTARY NOTE 5: REACTANCE MEASUREMENT WITH AC CURRENT***
In the AC+DC measurements, we apply a large DC current to drive skyrmions and superpose a small AC current for lock-in detection. In this case, only a single dynamical phase of the SkL (i.e., pin/creep/ow), determined by the magnitude of the DC current, contributes to the voltage response. In contrast, in the AC measurements, we apply a large AC current, which both drives skyrmions and is used for lock-in detection. In this case, over a full current cycle, the skyrmion velocity passes through multiple dynamical phases, all of which contribute to the measured voltage response, as discussed in Figs. 3a and b in the main text. Consequently, even when the amplitude of the AC current enters the ow region, the voltage response arising from the creep region still contributes to the measurement. This can explain the nonzero reactance signal observed in the ow regime.

To discuss this point in more detail, we calculate the current dependence of the reactance response measured with AC+DC current and with AC current using a toy model. In this calculation, we assume that the voltage is proportional to the time derivative of the current and that its coefficient depends on the current. Specifically, we adopt the following equation: $V = \left[e^{-(J+4)^2} + e^{-(J-4)^2}\right]dJ/dt$, in which in which the coefficient of dJ/dt peaks at $J = \pm 4$ and is nearly zero in both the low- and high-current regions (Fig. S6a). The current range where the coefficient is nonzero corresponds to the creep region in the experiment. Then, we calculate the time dependence of the voltage response for an AC+DC current $J = J_{DC} + J_{AC}\sin\omega t$ and an AC current $J = J_{AC}\sin\omega t$ (Gray-dotted curves in Figs. S6b and c). For the AC+DC current, the voltage response is nonzero only when J_{DC} lies within the current range where the coefficient is nonzero (Fig. S6b). In contrast, for the AC current, even when the amplitude of the AC current exceeds this current range, a finite voltage signal appears (Fig.S6c) because the current passes through the low-current region where the coefficient is nonzero.

From these time dependences of the voltage, we calculate the current dependence of the real and imaginary parts of the first-harmonic voltage, which corresponds to what we measured in our experiment. As shown in Fig. S6d, for the AC+DC current, the current dependence of the imaginary component exhibits a peak structure around J = 4, similar to the current dependence of the coefficient of dJ=dt. In contrast, for the AC current, the peak is smeared, and the signal persists even in the higher current region. This occurs because the current passes through the nonzero-coefficient region even at larger current amplitudes. The experimentally observed nonzero signal in the flow region is also explained by the same mechanism."

And made reference to this comment within the main text on Page 10, as follows:

> *"We note that the peak value of $Im[\rho_{xx}]$ shifts into the flow region at high frequencies. This originates from the AC current traversing the creep region before entering into the flow region (see Supplementary Note 5 for details)."*

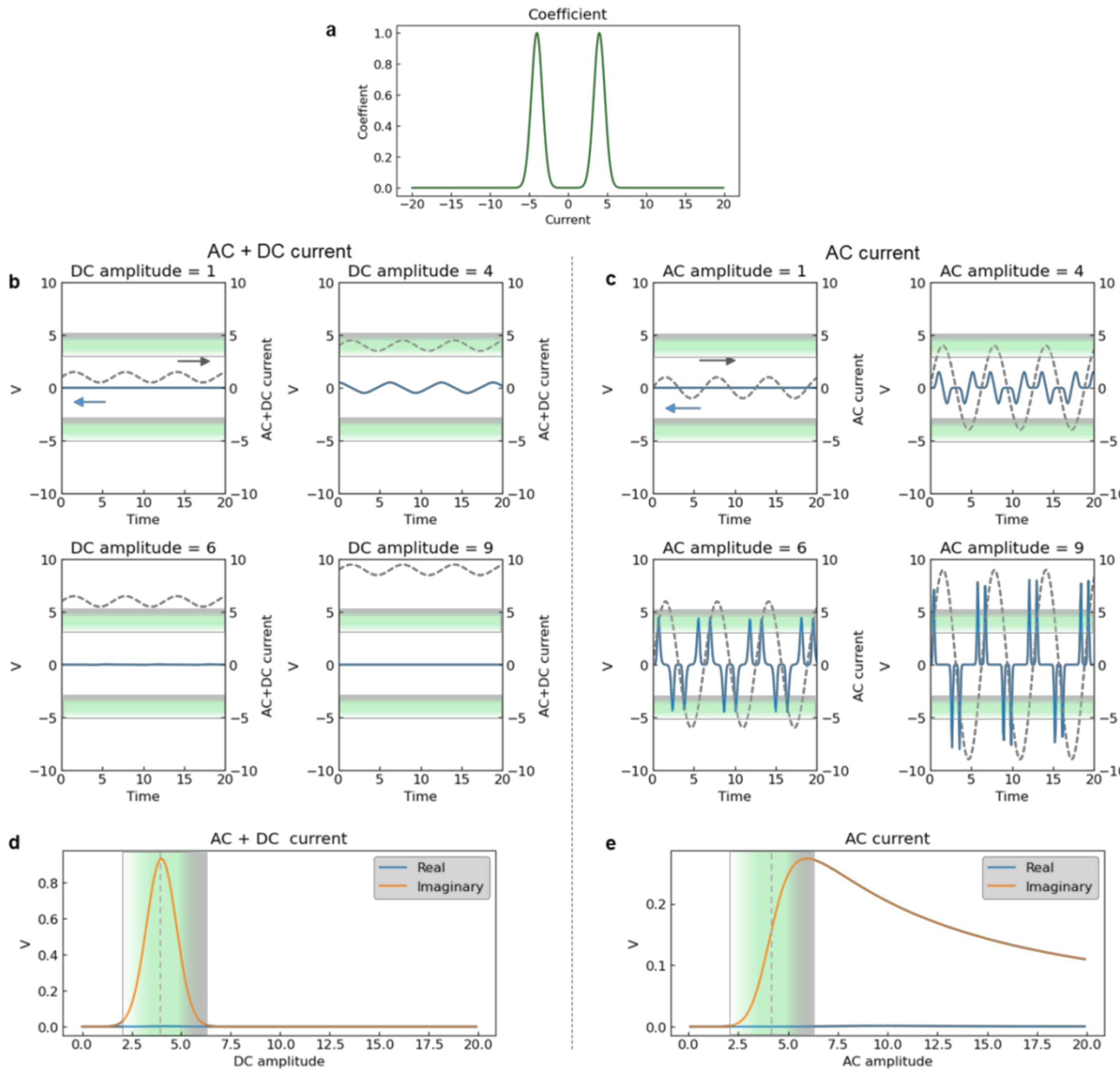

Figure R2: Calculation of the current dependence of the reactance signal. a Current dependence of the coefficient of dJ/dt. **b-c** Time dependence of the applied currents (grey dotted curves) and the corresponding voltage responses (blue curves) for AC+DC current (**b**) and AC current (**c**). The green shaded region indicates the current range where the coefficient of dJ/dt is nonzero. **d-e** Current dependence of the real (blue) and imaginary (orange) parts of the first-harmonic voltage for AC+DC current (**d**) and AC current (**e**). The vertical grey dotted lines denote the peak position of the coefficient of dJ/dt (J = 4), and the green shaded region indicates the current range where the coefficient of dJ/dt is nonzero.

Specific comment 4:

What is the justification for Eq. (7)?

The definitions of the pinned, creep and flow regions are somewhat difficult to define, since their boundaries typically appear as smeared crossovers caused by thermally activated processes rather than as sharp transitions. It is preferable to determine these regimes from minimally processed data and in a systematic manner following a set algorithm to maintain consistency in interpretation. To this end, we first fit $\Delta\rho_{yx}^{\mathrm{THE}}$ data using a specific equation. At present, there is no established theoretical formula for $\Delta\rho_{yx}^{\mathrm{THE}}$ that allows practical fitting of its

experimentally observed profile over the entire current range. However, $\Delta\rho_{yx}^{\mathrm{THE}}$ is known to exhibit the following behaviour: nearly zero in the pinned region, a linear increase in the creep region, and saturation in the flow region. In addition, between these regions, $\Delta\rho_{yx}^{\mathrm{THE}}$ varies smoothly due to thermal fluctuations [T. Schulz et al., **8**, 301 (2012) and M. T. Birch et al, **633**, 554 (2024)]. Equation (7), known as the error function, is one of the well-known functions that exhibit this type of profile. Indeed, we heuristically found that the current-density dependence of $\Delta\rho_{yx}^{\mathrm{THE}}$ can be well fitted by Eq. (7), although there is currently no theoretical justification of this equation based on the microscopic behaviour of skyrmion dynamics. Nevertheless, this empirical approach enables us to define the creep and flow thresholds in a consistent and reproducible manner (see also response #5). Therefore, we believe that the use of Eq. (7) is justified on empirical and heuristic grounds. In the revised manuscript, we have added this discussion in Supplementary Note 8 (shown after Specific comment 5) and have shown all the fitting results to demonstrate the quality of the fitting in Supplementary Fig. 8.

Specific comment 5:

The criteria for determining the C/F transition in Fig. S7 seems arbitrary. Why not use the extrapolation to the saturations value to be consistent with the criteria for P/C?

We determined both threshold currents in the most reasonable way based on the physical properties of each region. In the flow region, the skyrmions approach the conduction electron velocity, leading to the saturation of $\Delta\rho_{yx}^{\mathrm{THE}}$. Therefore, the flow region should be defined as the region where $\Delta\rho_{yx}^{\mathrm{THE}}$ becomes saturated. Consequently, we define the creep-to-flow (C/F) threshold as the point at which the difference between the fit to Eq. (7) and the saturated value of $\Delta\rho_{yx}^{\mathrm{THE}}$ is 0.1%.

For the pinned-to-creep (P/C) threshold, this boundary is strongly smeared due to thermal activation. Furthermore, it is also generally understood that creep motion can be induced by an infinitesimal current density with a finite probability at finite temperature [F. Kagawa, et al. Nat. Commun 8, 1332 (2017) and P. Chauve et al., PRB 62, 6241 (2000)]. Thus, if we were to define the P/C threshold from the current density at which $\Delta\rho_{yx}^{\mathrm{THE}}$ appears to take a finite value, similar to the definition of the C/F threshold, it would be strongly affected by the measurement accuracy of $\Delta\rho_{yx}^{\mathrm{THE}}$, as discussed in F. Kagawa, et al. Nat. Commun 8, 1332 (2017). To avoid such ambiguity, we define the P/C threshold in practice as the current density corresponding to the x-intercept of the extrapolated linear slope (gray dashed line in Fig. S8).

To understand how these boundaries change with variable frequency, a consistent approach in defining the C/F and P/C boundaries is necessary. We believe that the approach define above provides a good comparison to understand the correlation between each threshold and frequency when applied consistently.

In response to Specific Comments 4 and 5, we have provided a more explicit description of the boundary definitions, to make their demarcation clearer, in the Methods, and added a discussion of the validity of the threshold determination in Supplementary Note 8, as follows:

> **Methods:**
> ***"Determination of creep and flow thresholds***
> *The definitions of the pinned, creep, and flow regions are somewhat difficult to define, since their boundaries typically appear as smeared crossovers caused by thermally activated processes rather than as sharp transitions. In this study, to systematically and precisely determine the creep (J^C) and flow (J^F) thresholds, we first fit $\Delta Re[\rho_{yx}]$ according to the equation*
>
> $$\Delta Re[\rho_{yx}^{THE}] = a\left[\frac{1}{\sqrt{\pi}}\int_0^{\frac{J-b}{c}} e^{-t^2}dt + 1\right],$$
>
> *where a, b , and c are fitting parameters. This equation provides a simple yet effective phenomenological approximation of the current density dependence of $\Delta Re[\rho_{yx}^{THE}]$. The creep threshold J^C is defined as the current density corresponding to the x-intercept of the extrapolated linear slope. The flow threshold JF is defined as the current density at which the difference between the fit to Eq. 7 and the saturated value of $\Delta Re[\rho_{yx}^{THE}]$is 0.1%. More detailed discussion and an example of this procedure are provided in Supplementary Note 8 and Supplementary Fig. 8."*

“**Supplementary Note 8:**

As described in the Methods in the main text, the definitions of the pinned, creep, and flow regions are somewhat difficult to define, since their boundaries typically appear as smeared crossovers caused by thermally activated processes rather than as sharp transitions. In this study, to systematically and precisely determine the threshold current densities, we use Eq. (7) in the main text, known as the error function, and phenomenologically fit it to $\Delta Re[\rho_{yx}^{THE}]$. Equation (7) can approximately define three regimes: (1) a pinned region, where $\Delta Re[\rho_{yx}^{THE}]$ is zero, (2) a creep region, where $\Delta Re[\rho_{yx}^{THE}]$ increases linearly with current, and (3) a flow region, where $\Delta Re[\rho_{yx}^{THE}]$ is saturated. As shown in Fig. S8, this equation reproduces well the experimentally observed current dependence of $\Delta Re[\rho_{yx}^{THE}]$.

The threshold current densities are reasonably defined as follows: In the flow region, skyrmions catch up with the conduction electron velocity, leading to the saturation of $\Delta Re[\rho_{yx}^{THE}]$. Therefore, the flow region should be defined as the region where $\Delta Re[\rho_{yx}^{THE}]$ becomes saturated. Consequently, we define the creep-to-flow (C/F) threshold as the point at which the difference between the fit to Eq. (7) and the saturated value of $\Delta Re[\rho_{yx}^{THE}]$ is 0.1 %. For the pinned-to-creep (P/C) threshold, this boundary is strongly smeared due to thermal activation. Thus, if we were to define the P/C threshold from the current density at which $\Delta Re[\rho_{yx}^{THE}]$ appears to take a finite value, similar to the definition of the C/F threshold, it would be strongly affected by the measurement accuracy of $\Delta Re[\rho_{yx}^{THE}]$, as discussed in [12]. To avoid such ambiguity, we define the P/C threshold in practice as the current density corresponding to the x-intercept of the extrapolated linear slope (gray dashed line in Supplementary Fig. S8).”

Reviewer #3:

The manuscript presents an elegant and timely study of emergent reactance in a current-driven skyrmion lattice (SkL) in MnSi. By probing both the longitudinal and transverse components of the complex resistivity under AC excitation, the authors provide compelling evidence that internal skyrmion deformation plays a key role in emergent electromagnetic responses during the creep regime. The experimental results are significant for both fundamental understanding and potential device applications involving skyrmions in spintronic circuits.
The work is well-motivated and clearly written, and the data are thoughtfully analyzed. The findings will be of broad interest to the communities working on topological magnetism, emergent electrodynamics, and magneto-transport. I recommend publication after the following points are addressed:

We sincerely thank reviewer 3 for their insightful comments and positive review of our work. We address each point in order below:

Comment 1:

The manuscript presents the skyrmion motion in terms of three regimes: pinned, creep, and flow. However, these transitions are not sharp at finite temperature and are more accurately described as crossovers. It would improve the clarity and rigor of the paper to more explicitly define the criteria used to distinguish these regimes in both DC and AC measurements. For example, in Fig. 2 and Fig. 3, how is the onset of creep (J_C) and the beginning of flow (J_F) quantitatively determined? Are these thresholds extracted from fitting procedures or based on inflection points? A discussion acknowledging the crossover nature of these regimes, particularly at finite temperature, would make the interpretation more robust.

We thank Reviewer 3 for raising this important point. It is true that thermal fluctuations smear the boundaries between dynamical regimes such that they are better described as crossovers rather than phase transitions. We agree that explicitly discussing this notion indeed makes our interpretation of the results more robust.

In similarity to the points addressed by reviewer 2, the criteria used to distinguish these boundaries are based on fitting the change in the topological Hall resistivity, $\Delta\rho_{yx}^{\mathrm{THE}}$ (which is related to the skyrmion velocity). We will discuss the main points here (see also our responses to comments 4 and 5 from reviewer 2):

The dynamical phases of skyrmions exhibits three regions: (1) a pinned region, in which $\Delta\rho_{yx}^{\mathrm{THE}}$ = 0, (2) a creep region, in which $\Delta\rho_{yx}^{\mathrm{THE}}$ steadily changes, and (3) a flow region, in which $\Delta\rho_{yx}^{\mathrm{THE}}$ saturates. We approximate this transition using Eq. (7), known as the error function, which consists of three regions: nearly zero in the pinned region, a linear increase in the creep region, and saturation in the flow region. As there is no accepted equation describing this current profile, Eq. (7) provides a useful approximation to determine the creep and flow thresholds.

In the flow region, the skyrmions catch up with the conduction electron velocity, leading to saturation of $\Delta\rho_{yx}^{\mathrm{THE}}$. We therefore define the creep-to-flow (C/F) threshold as the point at which the difference between the fit to Eq. (7) and the saturated value of $\Delta\rho_{yx}^{\mathrm{THE}}$ is 0.1%. For the pinned-to-creep (P/C) threshold, this threshold is strongly smeared due to thermal activation, and thus applying a criterion similar to that for the C/F threshold would be considerably affected by the measurement accuracy of $\Delta\rho_{yx}^{\mathrm{THE}}$. Consequently, to avoid such ambiguity, we define the P/C threshold in practical as the current density corresponding to the *x*-intercept of the extrapolated linear slope (gray dashed line in Fig. S8).

In the revised manuscript, we have corrected the description of the dynamical transition and added the description of the crossover in the main text (page 7), as follows:

> *"the flow regime above J^F_{DC}. These boundaries (J^C_{DC} and J^F_{DC}) are typically smeared by thermal fluctuations and are regarded as crossovers. We determined them using the fitting procedure described in the Methods and Supplementary Note 8. Estimated J^C_{DC} and J^F_{DC} are of…"*

In addition, we have added more detailed discussions of the determination of the threshold current in Methods, Supplementary Note 8, and Supplementary Fig. S8, as reproduced here:

Methods:

"Determination of creep and flow thresholds

The definitions of the pinned, creep, and flow regions are somewhat difficult to define, since their boundaries typically appear as smeared crossovers caused by thermally activated processes rather than as sharp transitions. In this study, to systematically and precisely determine the creep (J^C) and flow (J^F) thresholds, we first fit $\Delta Re[\rho_{yx}]$ according to the equation

$$\Delta Re[\rho_{yx}^{THE}] = a\left[\frac{1}{\sqrt{\pi}}\int_0^{\frac{J-b}{c}} e^{-t^2}dt + 1\right],$$

where a, b , and c are fitting parameters. This equation provides a simple yet effective phenomenological approximation of the current density dependence of $\Delta Re[\rho_{yx}^{THE}]$. The creep threshold J^C is defined as the current density corresponding to the x-intercept of the extrapolated linear slope. The flow threshold JF is defined as the current density at which the difference between the fit to Eq. 7 and the saturated value of $\Delta Re[\rho_{yx}^{THE}]$is 0.1%. More detailed discussion and an example of this procedure are provided in Supplementary Note 8 and Supplementary Fig. 8."

Supplementary Note 8:

"As described in the Methods in the main text, the definitions of the pinned, creep, and flow regions are somewhat difficult to define, since their boundaries typically appear as smeared crossovers caused by thermally activated processes rather than as sharp transitions. In this study, to systematically and precisely determine the threshold current densities, we use Eq. (7) in the main text, known as the error function, and phenomenologically fit it to $\Delta Re[\rho_{yx}^{THE}]$. Equation (7) can approximately define three regimes: (1) a pinned region, where $\Delta Re[\rho_{yx}^{THE}]$ is zero, (2) a creep region, where $\Delta Re[\rho_{yx}^{THE}]$ increases linearly with current, and (3) a flow region, where $\Delta Re[\rho_{yx}^{THE}]$ is saturated. As shown in Fig. S8, this equation reproduces well the experimentally observed current dependence of $\Delta Re[\rho_{yx}^{THE}]$.

The threshold current densities are reasonably defined as follows: In the flow region, skyrmions catch up with the conduction electron velocity, leading to the saturation of $\Delta Re[\rho_{yx}^{THE}]$. Therefore, the flow region should be defined as the region where $\Delta Re[\rho_{yx}^{THE}]$ becomes saturated. Consequently, we define the creep-to-flow (C/F) threshold as the point at which the difference between the fit to Eq. (7) and the saturated value of $\Delta Re[\rho_{yx}^{THE}]$ is 0.1 %. For the pinned-to-creep (P/C) threshold, this boundary is strongly smeared due to thermal activation. Thus, if we were to define the P/C threshold from the current density at which $\Delta Re[\rho_{yx}^{THE}]$ appears to take a finite value, similar to the definition of the C/F threshold, it would be strongly affected by the measurement accuracy of $\Delta Re[\rho_{yx}^{THE}]$, as discussed in [12]. To avoid such ambiguity, we define the P/C threshold in practice as the current density corresponding to the x-intercept of the extrapolated linear slope (gray dashed line in Supplementary Fig. S8)."

Comment 2:

In Fig. 4, both the creep threshold (J_AC^C) and the flow threshold (J_AC^F) increase with frequency. This is somewhat counterintuitive, as increasing the AC frequency often aids depinning by shaking the skyrmions, which can help them overcome the local pinning potential. This would typically lead one to expect a decrease in J_AC^C and J_AC^F with frequency. The authors should address and explain this trend more carefully. Is it possible that the increased thresholds are due to the finite inertia of the skyrmions or limitations in their response time? A more detailed explanation would greatly strengthen the discussion.

The increase in the threshold current density is related to the inertia and consequent retardation of the skyrmions and can be explained intuitively as follows. Due to inertia, it takes a finite time for skyrmions to start moving, when a current is applied. In the present case, we apply an AC current, in which the polarity of the current is alternately reversed at a certain frequency. For depinning of the skyrmions, they must begin moving before the polarity of the current is reversed. At low-frequencies, skyrmions can move before the polarity of the AC current is reversed because the time interval between current-polarity changes is relatively long. However, at

higher frequencies, the polarity of the AC current is reversed more quickly, making it more difficult for skyrmions to start moving. Therefore, a larger current density is required for skyrmions to move, and the threshold current density increases at high frequencies. In the revised manuscript, we have added this discussion to the main text (pages 10), as reproduced in the following:

> *"In addition, with increasing frequency, $J^C{}_{AC}$ and $J^F{}_{AC}$ increase monotonically. This is because the inertia of the skyrmion causes a retardation of its motion, which particularly impedes rapid skyrmion motion at higher frequencies and raises the threshold current densities. Both $Im[\rho_{yx}]$ and $Im[\rho_{xx}]$…".*

Comment 3:

The authors argue that skyrmion deformation is reduced in the flow regime due to the averaging over pinning potentials. This is valid at moderate velocities, where the SkL moves smoothly and pinning is largely irrelevant. However, at higher skyrmion velocities, deformation can reappear due to spin-wave emission, as demonstrated in micromagnetic simulations (e.g., PRL 110, 207202 (2013)). Therefore, the statement that deformation is suppressed in the flow regime should be made more precise. The authors are encouraged to discuss under what conditions deformation might re-emerge, and whether such high-velocity effects could be observable in their frequency-current parameter space.

We thank Reviewer 3 for this insightful question. As Reviewer 3 pointed out, at larger driving currents, the spin-wave emission can indeed cause skyrmions to deform into a chiral liquid. In principle, the deformation of the skyrmion lattice into a chiral liquid may induce an emergent electric field. However, using typical parameters for a bulk skyrmion system, the authors of the referenced paper [PRL 110, 207202 (2013)] found that this transition to the chiral liquid phase requires a current density on the order of 10^{12} Am^{-2}, four orders of magnitude larger than the current densities used in our work. In our present setup, it is difficult to apply such a large current density due to the output limitation of a current source and the expected significant Joule heating. Thus, we regard this as a task for future investigation. In the revised manuscript, we have added discussion about these points in the main text (page 7), as follows:

> *"Therefore, aside from the higher thresholds, there is no essential difference in the current-driven properties of the SkL between our device and bulk samples. We note that, above the flow region, the re-deformation of the SkL via spin-wave emission has been theoretically predicted [37]. However, the current density used in this study is four orders of magnitude smaller than that required to induce such re-deformation."*

Minor Suggestions

The term "emergent reactance" is intriguing but non-standard. While it is defined via the imaginary part of the resistivity, it would be helpful to more explicitly contrast it with classical inductive or capacitive reactance for clarity.

We understand that the term "emergent reactance" is non-standard. However, we deem this to be the most appropriate term to describe the observed behaviour. This is based on the fact that reactance refers to any mechanism that may change the phase of a voltage response given an AC oscillation. To be more explicit, it is typical to refer to the positive reactance as inductance and the negative one as capacitance. However, in the present case, the sign of reactance is both positive and negative depending on the conditions such as magnetic field and temperature. It indicates that the observed phenomena can be both capacitive and inductive by tuning parameters. This is one of the novelties and advantages of our observation. Hence, referring to the mechanism as a reactance provides the most general description of the observed effect. Furthermore, we maintain that this effect is "emergent" as it originates from the collective dynamics of the spins constituting the skyrmions and skyrmion lattice in the form of emergent electric fields, hence referring to the phenomenon as "emergent reactance".

In the revised manuscript, we have explicitly described the definition of "emergent reactance" (Page 3), as reproduced in the following.

> *"This deformation can be described by spin-tilting and phason modes, whose excitation notably induces an out-of-phase emergent electric field relative to the input AC current, equivalent to a reactance [20, 21]. Hereafter, we refer to the out-of-phase response arising from the emergent electric field as "emergent reactance". This emergent reactance contrasts…"*

We hope that this explanation of our decision to term this effect "emergent reactance" convinces reviewer 3 that this term is the most general and appropriate description of the observed phenomenon.

Reviewer #1:

The authors have addressed all my concerns. I believe this work would contribute to the understanding of skyrmion crystal dynamics from a different point of view. I would like to recommend the publication of this manuscript.

We sincerely thank the reviewer for their time in reviewing our manuscript and are happy to see that all concerns have been addressed.

Reviewer #2:

The authors have addressed the comments and concerns I raised in my first review of the manuscript. Furthermore, it is my opinion that they have also answers criticisms raised by the other two referees.

I would also like to commend the authors on the thoroughness of their response and for approaching the reviews in a constructive manner (a nice change from the dismissive tone often used when responding to a peer review).

To summarize, I recommend that the paper is accepted for publication.

We sincerely thank the reviewer for their time in reviewing our manuscript and are happy to see that the reviewer believes all their comments and concerns have been addressed.

Reviewer #3:

The authors have provided detailed replies to all reviewer comments and made substantial revisions to the manuscript. While many of their responses are satisfactory, I find that their reply to Comment 1 remains inadequate and does not fully address the concern.

We sincerely thank the reviewer for their time in reviewing our manuscript and are happy to see that the reviewer is satisfied with nearly all of the comments and concerns. Below, we have addressed the final concern related to Comment 1 of the initial review.

In the revised manuscript, the authors introduce a phenomenological fitting function to analyze their data and to define the depinning, creep, and flow regimes. However, the physical motivation for the specific choice of this fitting function remains unclear. The authors describe the function as an empirical form (an error function) that reproduces the observed current dependence but do not justify why this particular functional form should capture the underlying skyrmion dynamics.

We sincerely thank the reviewer for this comment and would like to make some brief comments here before discussing the details. It is true that we use an empirical fitting function to determine the threshold current densities. However, as detailed below, our definition is justified based on previous studies of current-induced skyrmion dynamics [e.g., T. Schulz et al., Nat. Phys. **8**, 301 (2012) and M. T. Birch et al, Nature **633**, 554 (2024)]. In addition, we confirmed that our definition is not significantly affected by the specific choice of fitting function or criterion, demonstrating that other functions with S-shaped profiles and different criteria also give similar threshold values. Moreover, the reactance signals are observed well inside the creep regime rather than at the phase boundaries. Therefore, the small ambiguity in determining the thresholds does not affect our main conclusion.

Justification of our definition

The pinned, creep, and flow regions have been determined from the response of the topological Hall resistivity [T. Schulz et al, Nat. Phys. **8**, 301 (2012) and M. T. Birch et al, Nature **633**, 554 (2024)]. In the pinned regime, as the skyrmion velocity $v_{Sk} = 0$, there is no change in the topological Hall resistivity i.e. $\Delta\mathrm{Re}[\rho_{yx}^{\mathrm{THE}}] = 0$. In contrast, in the flow regime, the skyrmion velocity with respect to the electron velocity reaches a saturation value, and thus the change in the topological Hall resistivity is saturated. The creep regime is then defined as the

current range between the pinned and the flow regimes. Therefore, to denote boundaries between each phase, we should (1) find the point at which $\Delta\mathrm{Re}[\rho_{yx}^{\mathrm{THE}}]$ deviates from zero, and (2) determine the current density at which $\Delta\mathrm{Re}[\rho_{yx}^{\mathrm{THE}}]$ reaches its saturation value, which correspond to the pinned-to-creep (P/C) and creep-to-flow (C/F) thresholds, respectively. Notably, in the above previous studies, these two points were identified by hand or by using phenomenological fitting. Therefore, our procedure, namely the determination of these points from empirical fitting, is not unusual compared with the procedures used in the previous studies.

Moreover, the definition of the thresholds for depinning, creep, and flow—such as the pinned-to-creep (P/C) and creep-to-flow (C/F) boundaries—depends sensitively on this arbitrary choice of function. Since these regimes are inherently crossovers rather than sharp transitions, the threshold values extracted in this way lack robustness and may vary with alternative fitting procedures. Therefore, the physical meaning of the defined boundaries is ambiguous.

It may not be possible to draw a clear distinction between the creep and flow regions, given their crossover nature. The authors should instead discuss how their results depend on the choice of the criteria used to separate these two regimes and evaluate the stability of their conclusions with respect to different definitions or fitting functions. Without such clarification, the claims regarding the precise identification of depinning, creep, and flow thresholds are not convincingly supported.

Ambiguity in determining the threshold current densities

In the previous version of the manuscript, to systematically and precisely determine the P/C and C/F thresholds based on the above definitions, we used an error function. However, our choice of the error function is not crucial for determining the threshold values, and other functions with similar forms are also expected to provide similar threshold values.

As pointed out by the reviewer, it is important to discuss how our threshold values depend on the fitting function forms and the criterion. Thus, we have performed further analysis of the determination of the P/C and C/F thresholds using functional forms defined as follows:

Function 1 (Error Function (Erf.)):

$$\Delta\mathrm{Re}[\rho_{yx}^{\mathrm{THE}}] = a\left[\frac{1}{\sqrt{\pi}}\int_0^{\frac{J-b}{c}} e^{-t^2}dt + 1\right]$$

Function 2 (Sigmoid (Sig.)):

$$\Delta\mathrm{Re}[\rho_{yx}^{\mathrm{THE}}] = \frac{2a}{1 + e^{-\left(\frac{J-b}{c}\right)}}$$

Function 3 (Gompertz (Gom.) – asymmetric sigmoid):

$$\Delta\mathrm{Re}[\rho_{yx}^{\mathrm{THE}}] = 2ae^{-e^{-\left(\frac{J-b}{c}\right)}}$$

in which a, b and c are fitting parameters. All functions exhibit an S-shaped profile, in which $\Delta\mathrm{Re}[\rho_{yx}^{\mathrm{THE}}]$ varies from 0 at low J to its constant value at large J, but the detailed profiles between these two limits are different. In determining the threshold values, we use the same protocol to that used in our prior response: (1) fit the functional form to $\Delta\mathrm{Re}[\rho_{yx}^{\mathrm{THE}}]$, (2) fit a linear slope to the creep region, (3) determine the P/C threshold from the intersection of this linear slope with $\Delta\mathrm{Re}[\rho_{yx}^{\mathrm{THE}}] = 0$, and (4) determine the C/F threshold by the point at which $\Delta\mathrm{Re}[\rho_{yx}^{\mathrm{THE}}]$ saturates within a defined tolerance.

To test the robustness of our protocol, we performed this analysis on the dataset shown in Fig. 3 of the main text, using each function defined above while changing the tolerance for the C/F threshold within a range from 0.05% to 1%. In Figs. R1a-c, we show the P/C thresholds with dotted lines. The P/C thresholds are almost the same for all three functions, as also confirmed by the bar chart in Fig. R1d. We also present the C/F thresholds for tolerances of 0.05%, 0.1% and 0.5% in Figs. R1a-c with dashed lines. For all tolerances within the tested range, the C/F thresholds are plotted in Fig. R1e. Clearly, the obtained P/C threshold well represents the current density

at which $\Delta\rho_{yx}$ reaches its saturation value, indicating that the specific choice of fitting function form and tolerance does not lead to a meaningfully different value of the P/C threshold.

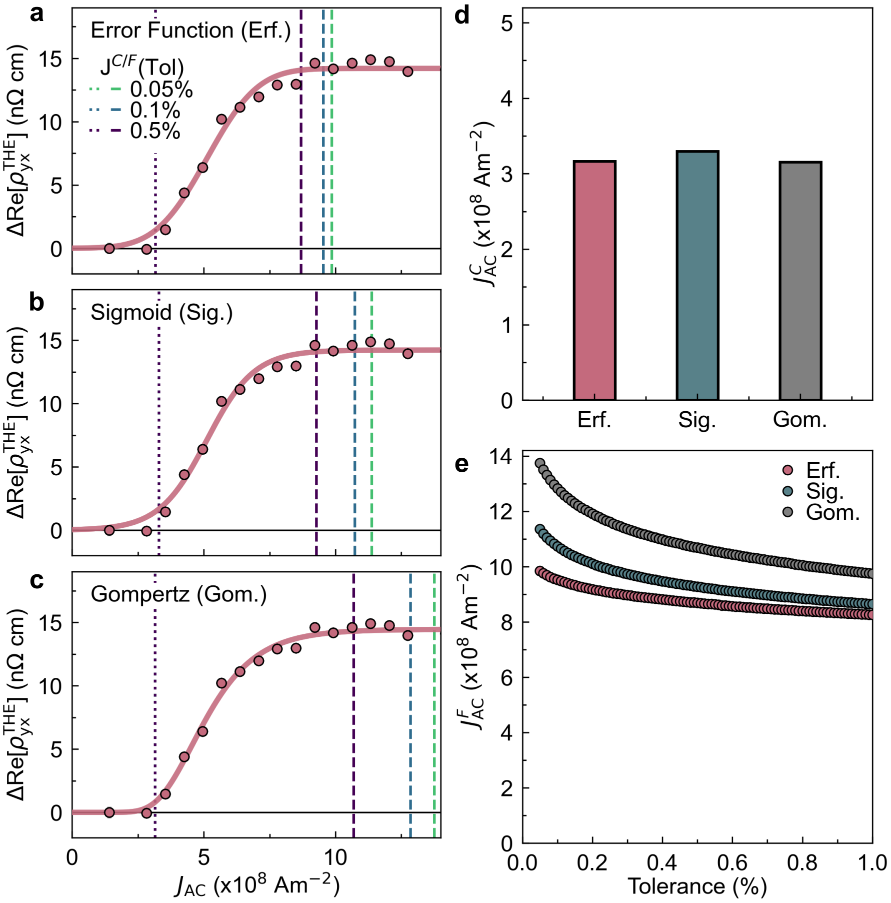

Fig. R1: (a-c) Data taken from Fig.3 of main text and fit with functions 1 (Error function (Erf.)), 2 (Sigmoid (Sig.)), and 3 (Gompertz (Gom.)) as defined in the text. (d) P/C thresholds determined by using each function. (e) Tolerance dependence on the C/F threshold for each function.

Then, we calculate the mean and standard error for the three functions across the full range of tolerances (0.05% to 1%), which allows us to evaluate the ambiguity in determining the threshold values. In Fig. R2, we show the $\Delta\mathrm{Re}[\rho_{yx}^{\mathrm{THE}}]$, $\mathrm{Im}[\rho_{yx}]$ and $\mathrm{Im}[\rho_{xx}]$ as a function of the current density together with the threshold current densities and their error bars. The averaged threshold values (i.e., the positions of the dashed lines) are almost the same to those shown in Fig. 3 of the previous manuscript. In addition, the error bar for the P/C thresholds are comparable to the point size, and the error bar for the C/F thresholds are also moderate. Consequently, even when considering the error bars of the threshold values, it is clear that the emergent reactance signals are observed mainly in the creep region (Figs R2 d and e). We note that the remaining emergent reactance signals in the flow region result from the measurement using AC current as already discussed in Supplementary Note 5.

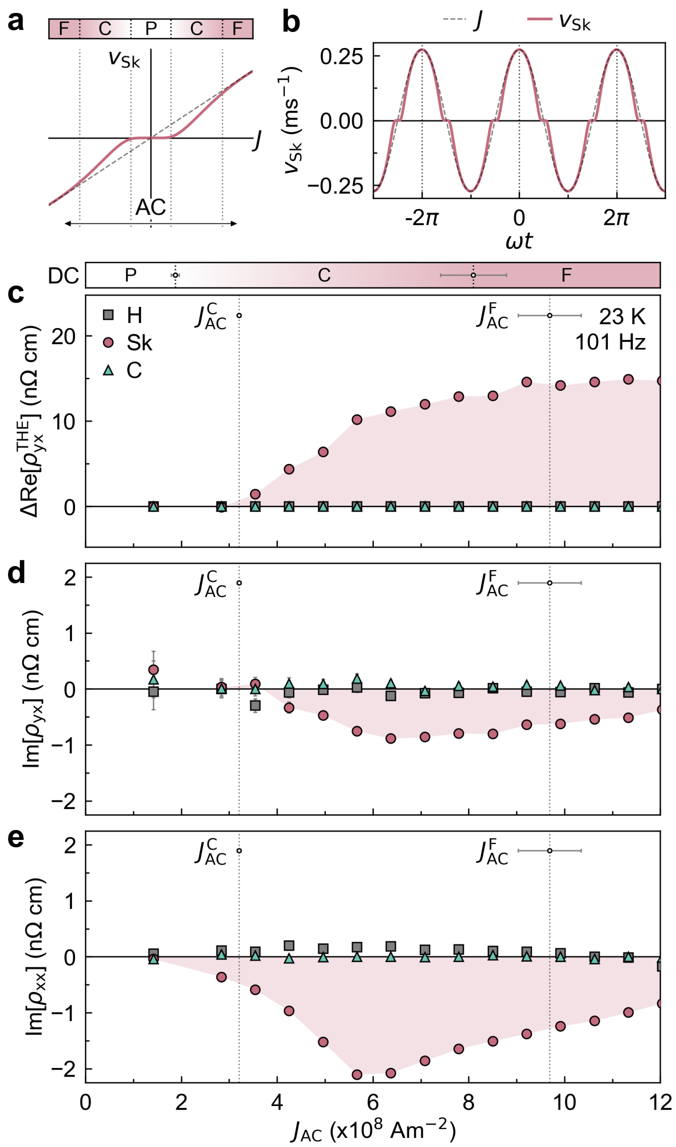

*Fig. R2: Figure 3 reproduced and adapted from the main text. Current density dependence of emergent reactance in MnSi **a,b** Schematic representation of the skyrmion velocity curve for a cycle of AC. The voltage response follows the SkL velocity, but the time-averaged response is primarily governed by the dynamical phase where the current reaches its peak. **c-e** AC amplitude, J_{AC} , dependence of $\Delta Re[\rho_{yx}^{THE}]$ (**c**), $Im[\rho_{yx}]$ (**d**), and $Im[\rho_{xx}]$ (**e**) for helical (H, gray squares), skyrmion lattice (SkL, red circles), and conical phases (C, teal triangles), measured at 23 K and 101 Hz. The threshold current densities for the creep, J_{AC}^{C}, and flow, J_{AC}^{F}, determined from $\Delta Re\left[\rho_{yx}^{THE}\right]$ are indicated by the dotted vertical lines, with errors on threshold values highlighted with horizontal error bars. For procedure of error estimation, see Methods. The threshold current densities in the DC measurement are shown at the top of **c**. Error bars in **c**-**e** correspond to the standard deviation of signal estimated in the field polarised regime. Where not visible, error bars are smaller than the data points.*

Repeating this analysis across all frequencies, we also show the threshold current densities with error bars together with colour maps of $\Delta\mathrm{Re}[\rho_{yx}^{\mathrm{THE}}]$, $\mathrm{Im}[\rho_{yx}]$ and $\mathrm{Im}[\rho_{xx}]$ in the frequency-current density plane in Fig. R3. Again, the averaged threshold values (i.e., the positions of the markers) are almost the same to those shown in Fig. 4 of the previous manuscript. Regarding the size of the error bars, for the P/C thresholds, the error bars are much smaller (typically smaller than the point size) for all frequencies, clearly indicating that the functional form does not significantly affect the P/C threshold values. For the C/F thresholds, although the error bars are larger than those for the P/C thresholds, they are also still moderate for all frequencies, indicating that our protocol accurately determines the threshold current densities with small ambiguity, irrespective of a specific choice of functional form and tolerance.

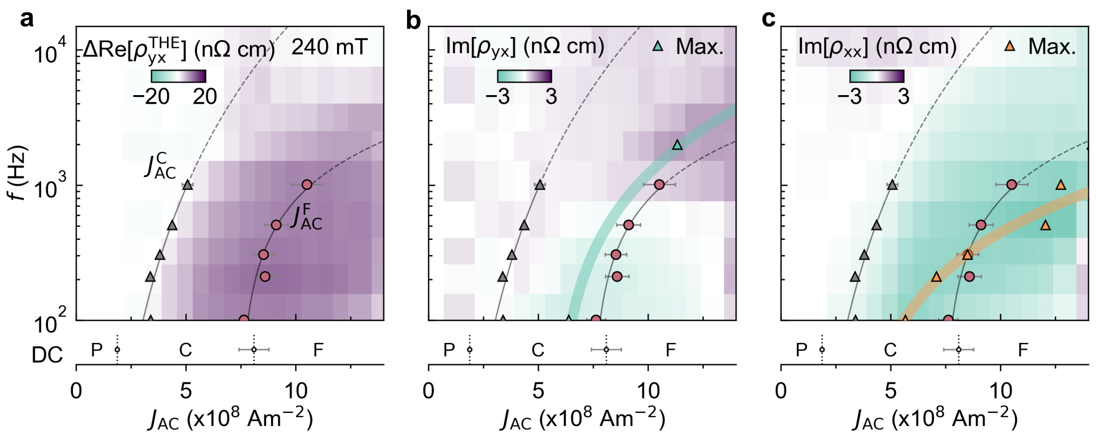

Fig. R3: Updated main text Fig 4. Error bars on J_{AC}^{C} and J_{AC}^{F} are estimated across varying tolerances and using different empirical function forms to fit $\Delta Re\rho_{yx}^{THE}$.

Effect of ambiguity on our main conclusion

Finally, we discuss the influence of ambiguity in determining the thresholds on our main conclusion in this work. We argue that the deformation of skyrmions in the creep regime leads to the emergent reactance signals ($\mathrm{Im}[\rho_{yx}]$ and $\mathrm{Im}[\rho_{xx}]$). These signals appear inside the creep regime rather than at the phase boundaries. In fact, as shown in Figs. R2 and R3, even when considering the error bars of the threshold values, it is clear that the peaks of the signals are observed mainly in the creep region. Note that the shifts of the peak values of $\mathrm{Im}[\rho_{xx}]$ into the flow region at high frequencies (Fig. 3c) arise from the AC current measurement as already discussed in Supplementary Note 5. Therefore, small ambiguity in determining the threshold current densities does not affect our main conclusion.

In light of these discussions, we have included the following analysis and discussion within the supplementary material and amended the Methods section outlining our updated protocol. Furthermore, we have revised Figures 2, 3 and 4 of the main text (Pages 6, 8, and 9, respectively) to include error bars to highlight the effects of alternative fitting procedures.

Amended Methods Section – Page 16, Main Text

"The definitions of the pinned, creep, and flow regions are somewhat difficult to define, since their boundaries typically appear as smeared crossovers caused by thermally activated processes rather than sharp transitions. In this study, to systematically and precisely determine the creep (J^C) and flow (J^F) thresholds, we fit $\Delta Re[\rho_{yx}^{THE}]$ according to the following three phenomenological equations

$$\Delta\mathrm{Re}[\rho_{yx}^{\mathrm{THE}}] = a\left[\frac{1}{\sqrt{\pi}}\int_0^{\frac{J-b}{c}} e^{-t^2}dt + 1\right]$$

$$\Delta\mathrm{Re}[\rho_{yx}^{\mathrm{THE}}] = \frac{2a}{1+e^{-\left(\frac{J-b}{c}\right)}}$$

$$\Delta\mathrm{Re}[\rho_{yx}^{\mathrm{THE}}] = 2ae^{-e^{-\left(\frac{J-b}{c}\right)}}$$

where a, b, and c are fitting parameters. These equations provide a simple yet effective phenomenological approximation of the current density dependence of $\Delta Re[\rho_{yx}^{THE}]$ without complete knowledge of the underlying dynamics. In addition, the use of three different equations helps reduce the ambiguity in determining the threshold current densities that arises from the specific choice of fitting function. Each equation describes a variation in $\Delta Re[\rho_{yx}^{THE}]$ from zero in the pinned regime, through a creep region, to its maximal value in the flow regime. For each function, the creep threshold J^C is defined as the current density corresponding to the x-intercept of an extrapolated linear fit to the

crossover region. The flow threshold J^F is defined as the current density at which the difference between the fitted value of $\Delta Re[\rho_{yx}^{THE}]$ and its saturated value approaches a defined tolerance. Then, we calculate the average of all the values obtained from the three phenomenological equations while varying the tolerances between 0.05-1% and present them together with the standard error. More detailed discussion and an example of this procedure are provided in Supplementary Note 8 and Supplementary Figs. 8. and 9."

Amended Supplementary Note 8 – Pages 20-22, Supplementary Information

"As described in the Methods in the main text, the definitions of the pinned, creep, and flow regions are somewhat difficult to define, since their boundaries typically appear as smeared crossovers caused by thermally activated processes rather than sharp transitions. It is therefore appropriate to define criteria based on physical considerations for the pinned-to-creep (P/C) and creep-to-flow (C/F) thresholds, which are then determined through a phenomenological fitting procedure. The threshold current densities are reasonably defined as follows: In the flow region, skyrmions catch up with the conduction electron velocity, leading to the saturation of $\Delta Re[\rho_{yx}^{THE}]$. Therefore, the flow region should be defined as the region where $\Delta Re[\rho_{yx}^{THE}]$ becomes saturated. Consequently, we define the C/F threshold as the point at which the difference between the fitted value of $\Delta Re\left[\rho_{yx}^{THE}\right]$ and its saturated value approaches a defined tolerance. In the pinned regime $\Delta Re\left[\rho_{yx}^{THE}\right] = 0$, and so the P/C threshold should be defined as the point at which $\Delta Re[\rho_{yx}^{THE}]$ deviates from zero. This boundary is strongly smeared due to thermal activation, and so if we were to define the P/C threshold from the current density at which $\Delta Re[\rho_{yx}^{THE}]$ appears to take a finite value, similar to the definition of the C/F threshold, it would be strongly affected by the measurement accuracy of $\Delta Re[\rho_{yx}^{THE}]$, as discussed in [12]. To avoid such ambiguity, we define the P/C threshold in practice as the current density corresponding to the *x*-intercept of an extrapolated linear slope that is fit to the creep region.

To systematically and precisely determine the P/C and C/F thresholds based on the above definitions, we fit $\Delta Re[\rho_{yx}^{THE}]$ with phenomenological equations. Here, we use three functions: the error function, the sigmoid function, and the Gompertz function [Eqs. (7)-(9) in the main text]. The use of three different equations helps reduce the ambiguity in determining the threshold current densities that arises from the specific choice of fitting function. Each equation is an S-shaped curve, in which we can approximately define three regimes according to the criteria defined above: (1) a pinned region, where $\Delta Re[\rho_{yx}^{THE}]$ is zero, (2) a creep region, where $\Delta Re[\rho_{yx}^{THE}]$ increases with current, and (3) a flow region, where $\Delta Re[\rho_{yx}^{THE}]$ is saturated. In Fig. S8, we show fits to the error function (Eq. (7)) as an example, which closely reproduces the experimentally observed current dependence of $\Delta Re[\rho_{yx}^{THE}]$. For this representative example, the gray dashed lines indicates the P/C threshold (J^C) and C/F threshold (J^F) with a tolerance of 0.1%.

We performed this analysis for each of the three functions while varying the tolerance for the C/F threshold within a range from 0.05% to 1%. These results for the data set shown in Fig. 3 of the main text are shown in Fig. S9. In Figs. S9a-c, we show the P/C thresholds with dotted lines, and C/F thresholds with dashed lines, for tolerances of 0.05%, 0.1% and 0.5%. The P/C thresholds are almost the same for all three functions, as also confirmed by the bar chart in Fig. S9d (Note that the P/C transition is, by definition, independent of the tolerance). For all tolerances between 0.05-1%, the C/F thresholds are plotted in Fig. S9e. Clearly, the specific choice of fitting function form and tolerance does not lead to a meaningfully different value of the of P/C threshold; the defined P/C threshold well represents the current density at which $\Delta Re[\rho_{yx}^{THE}]$ reaches its saturation value. Then, to properly indicate the ambiguity in determining the thresholds, we calculate the average of all the values obtained from the three phenomenological equations while varying the tolerances between 0.05-1% and present them together with the standard error."

Reviewer #3:

The authors have satisfactorily addressed all of my concerns regarding the phenomenological fitting used to define the pinned/creep/flow thresholds. Given these revisions and clarifications, I recommend acceptance of the current version for publication.

We sincerely thank the reviewer for their time in reviewing our manuscript and are happy to see that all concerns have been addressed.
